# Supplementary material for: Fractionated degradation and valorization of polypropylene waste into sulfonate surfactants
Source: Nat Commun. 2025 Nov 25;16:11611. doi: 10.1038/s41467-025-66592-0 (PMC12748851; doi:10.1038/s41467-025-66592-0)
Supplement: Supplementary file 1 — Supplementary Information [file 41467_2025_66592_MOESM1_ESM.pdf]

Supplementary Information

for

Fractionated degradation and valorization of polypropylene waste into sulfonate surfactants

Zhen Xu,[1,#,*] Yang Zhang,[1,#] Tao Wang,[2,#] Rong Yang,[1] Hao Sun,[3] Meiling Chen,[4] Feng Liu,[5] Jianjun Xu,6 Kai-Jie Chen,[1] Qikun Zhang,[4,*] Fuping Pan[1,*]

[1] School of Chemistry and Chemical Engineering, Northwestern Polytechnical University, Xi'an 710072, China

[2] Institute for Advanced Study, Tongji University, Shanghai 200092, China

[3] School of Fintech, Dongbei University of Finance and Economics, Dalian 116025, China

[4] Department of Chemistry, Chemical Engineering and Materials Science, Ministry of Education Key Laboratory of Molecular and Nano Probes, Shandong Normal University, Jinan 250014, China

[5] School of Management Science and Engineering, Dongbei University of Finance and Economics, Dalian, China

[6] Institute of Supply Chain Analytics, Dongbei University of Finance and Economics, Dalian 116025, China

[#] These authors contributed equally to this work.

* Corresponding author emails: zhen1@nwpu.edu.cn; zhangqk@sdnu.edu.cn; fupingpan@nwpu.edu.cn

Table of Contents

Experiments....4
Instrumentations....6
Characterization of degradation products....8
Characterization of PP upcycling products....10
Fractionated degradation modelling....11
Fractionated degradation simulation by ASPEN....13
Technoeconomic analysis (TEA) and life cycle analysis (LCA)....15
Supplementary discussion and overview....18
Field research of PP waste supply and processing work flow....18
Disadvantage of the non-fraction reactors....18
Fractionated degradation process and advantages....19
β-Scission, side reactions, and impact of contamination....20
Discussion of non-ideality....23
Distribution assessment....26
Characteristic of real-life plastic waste....26
Applicability for mixed and contaminated polyolefin wastes....28
Potential biodegradation pathway....29
Outlook....29
Supplementary Figure 1....31
Supplementary Figure 2....32
Supplementary Figure 3....33
Supplementary Figure 4....34
Supplementary Figure 5....35
Supplementary Figure 6....36
Supplementary Figure 7....37
Supplementary Figure 8....38
Supplementary Figure 9....40
Supplementary Figure 10....41
Supplementary Figure 11....42
Supplementary Figure 12....43
Supplementary Figure 13....44
Supplementary Figure 14....45
Supplementary Figure 15....46
Supplementary Figure 16....47
Supplementary Figure 17....48
Supplementary Figure 18....49
Supplementary Figure 19....50
Supplementary Figure 20....51
Supplementary Figure 21....52
Supplementary Figure 22....53
Supplementary Figure 23....54

Supplementary Figure 24 ... 55
Supplementary Figure 25 ... 56
Supplementary Figure 26 ... 57
Supplementary Figure 27 ... 58
Supplementary Figure 28 ... 59
Supplementary Figure 29 ... 60
Supplementary Figure 30 ... 61
Supplementary Figure 31 ... 62
Supplementary Figure 32 ... 63
Supplementary Figure 33 ... 64
Supplementary Table 1 ... 65
Supplementary Table 2 ... 66
Supplementary Table 3 ... 67
Supplementary Table 4 ... 68
Supplementary Table 5 ... 69
Supplementary Table 6 ... 70
Supplementary Table 7 ... 71
Supplementary Table 8 ... 71
Supplementary Table 9 ... 72
Supplementary Table 10 ... 73
Supplementary Table 11 ... 74
Supplementary Table 12 ... 75
Supplementary Table 13 ... 76
Supplementary Table 14 ... 77
Supplementary Table 15 ... 78
Supplementary Table 16 ... 79
Supplementary Table 17 ... 80
Supplementary References ... 81

Experiments

Materials

PP (M_n: 81.9 kDa, M_w: 387 kDa) and other reagents were purchased from Sigma-Aldrich with purity >99%, unless otherwise stated. SO_3 was obtained from Xingzhilian Ltd. Zibo, China), synthesized by SO_2 oxidation over V_2O_5 at 500°C. Real-life PP wastes were purchased from a municipal recycling depot (W-heze, Heze City, Yuncheng, China, Supplementary Figure 19) and Jieshou (W1, W2, W3) without cleaning. These real-life PP wastes were contaminated with complicated impurities (Supplementary Figures 19 and 22).

Experimental procedure

1. Reaction temperature calibration and pressure monitoring.

The reaction temperature of reactors with three-, single-, and none-fraction were calibrated using a thermocouple thermometer. Specifically, the thermocouple probe was placed at the bottom of the reactor (Supplementary Figures 4 and 5). The heating mantle temperature was gradually increased, and the thermocouple readings were monitored until the temperature stabilized at the target value for 30 minutes. The reactor pressure during the reaction was directly measured using a digital pressure meter equipped with needle probes.

2. PP degradation in three-fraction reactors.

Laboratory-grade PP (10 g) or real-life waste PP (W1, W2, W3) was loaded into a three-fraction reactor consisting of a degradation unit (~100 mL) and trays (~20 mL each, Supplementary Figure 5). The reactor was seamlessly molded to ensure airtightness and was set up in an Ar-filled glovebox to avoid oxygen contamination. Inside the glovebox, the top tray was connected to a total condenser via a frosted neck and sealed with vacuum grease. The condenser was plugged with a fluorinated stopper and connected to a gas bag. The setup was moved out of the glovebox and placed in a heat mantle. To optimize thermal insulation and concentrate heat within the degradation unit, an thick asbestos layer was installed atop the electric heating jacket. The system maintained a heating rate of ~50 °C min^{-1}. Temperature monitoring was achieved through a thermocouple integrated into the heating assembly, providing continuous verification that operational parameters remained within the prescribed range throughout the reaction duration. After a few hours, the reactor was cooled to room temperature for characterizations. After reaction, the setup was cleaned in a muffle furnace and then washed with aqua regia. The cleaned reactor was rinsed with DI water and stored in a desiccator for next use.

3. PP degradation in single-fraction reactors.

The ST reactor consists of three main components: a heating unit (150 mL volume), a tray, and a total condenser (Supplementary Figure 4). The reactor body was precision-molded to ensure complete airtightness. Approximately 10 g of polymer sample was loaded into the reactor, which was then transferred to an argon-filled glovebox to prevent oxygen contamination. Inside the glovebox, the condenser was connected to a grease-sealed frosted glass flask for liquid product collection. The assembled system was placed in a heating mantle and gradually heated to 400 °C (heating rate: ~50 °C min^{-1}; pressure: ~1 atm). After 5 hours, the reactor was cooled to ambient temperature, and the condensed liquid products were collected for subsequent analyses. For reactor maintenance, the setup was first calcined in a muffle furnace, followed by aqua regia washing. Finally, the reactor was thoroughly rinsed with deionized (DI) water and stored in a desiccator for next use.

4. PP degradation in none-fraction reactor.

The experimental setup replicated our previously reported system,[1] with similar reactor geometry and

operational parameters. A minor difference is inclusion of bay structures on the condenser for larger scale of reactions (Supplementary Figure 4). Approximately 10 g of polymer was loaded into the reactor, which was subsequently transferred into an argon-filled glovebox. The reactor assembly was sealed using a quartz glass plate secured by stainless steel clamps and a fluorinated O-ring for enhanced pressure resistance. Positioned in a heating mantle, the reactor was heated to 400 °C (heating rate: ~50 °C min^{-1}; pressure: ~1 atm). During pyrolysis, evolved vapors condensed on the water-cooled inner surfaces of the attached condenser. A specially designed bay structure effectively captured heavier condensates through gravitational draining. After 5 hours of continuous operation, condensed products were recovered via either (i) mechanical scraping or (ii) solvent extraction using refluxing hexanes, chloroform, or tetrahydrofuran.

5. Upcycling scale-up using real-life plastic wastes.

The fractional degradation process was scaled up to 10 kg using W-heze. Approximately 10 kg of pulverized waste was loaded into a single-fraction reactor through a flange structure. The flange was then sealed with a steel cover and secured with a hoop. The setup was purged with high-purity nitrogen (~ 100 mL s^{-1}) for 15 minutes. The heating and stirring units were activated (~3 °C min^{-1} and ~50 rpm) to initiate the reaction. After ~2-4 h, the reaction chamber reached 400°C. Entrainment was observed in the reactor chamber, while no entrainment was detected in the collector. During the reaction, the tray temperature rapidly rose from r.t. to ~ 220°C. The hydrocarbon flowed into the collecting chamber, exhibiting a light yellowish color. After 6 hours, the reaction was halted, and the products (PP-STW-6h) was drained into a 10-L sample bottle. The hoop on the flange was removed, and any solid residue adhering to the inner wall was scraped off using a shovel. The bottom flange was opened to collect the residue. The yields were measured using a platform balance with an accuracy of 0.1 kg.

6. Sulfonation by $ClSO_3H$/Diox.

A flat-bottom flask and a constant-pressure separation funnel were dried at 120°C for at least 12 h. The hot glassware was instantly transferred into an Ar-filled glovebox and sealed with fluorinated stoppers. After cooling to room temperature, PP-T2-5h (1.0 g, $n_{C=C}$ = 3.97 mmol g^{-1}), dissolved in 10 mL chloroform, was transferred to the flask using a 10 mL Teflon® syringe. Simultaneously, $ClSO_3H$/Diox (Diox 0.70 g, 7.94 mmol, chlorosulfonic acid, 0.56 g, 4.76 mmol) and 10 mL chloroform, were transferred to the separation funnel using a Teflon® syringe equipped with a ceramic needle. The flask was then cooled with an ice-salt bath. The sulfonation reagent was added dropwise into the flask. The pressure of the sulfonation setup was released through an outlet in the separation funnel. The gas was absorbed using an $AgNO_3$ aqueous solution (0.1 mol L^{-1}). The precipitate was characterized by XRD.

a. Aging: The sulfonation was stopped after 30 min, and the mixture was allowed to age at room temperature for another 30 min.

b. Hydrolysis: An aqueous base solution (NaOH, ~ 1 M) was added dropwise into the reaction mixture under vigorous stirring, forming a yellowish emulsion. Subsequently, the hydrolysis of the sulfonated mixture was conducted in a 100 mL hydrothermal reactor (170°C, ~ 1 MPa) for 30 min. After cooling to room temperature, the sulfonated products were harvested by cooking-off water using a rotary evaporator (60°C under reduced pressure) and then extracted using $CHCl_3$ (50 mL), assisted by an ultrasonic bath. Insoluble salts were removed by filtration or centrifugation. The $CHCl_3$ solution was evaporated under reduced pressure, leaving light yellow solid surfactants. The product was scraped off and stored in a desiccator for later experiments.

7. Sulfonation by SO_3.

The sulfonation setup (Supplementary Figure 3) was dried at 120°C for 12 h. After drying, the setup was rapidly purged with an Ar and allowed to cool in an ice bath. Under an Ar atmosphere, hydrocarbon (~10 g, PP-NT-5h or PP-T2-5h) was added to the setup and diluted with chloroform at a 1:2 volume ratio. The gas

inlet was connected to a buffer bottle, which was in turn linked to an SO_3 tank and an air tank. The SO_3/air mixture (2 mL s^{-1}) was introduced into the chloroform solution under vigorous stirring. The SO_3 concentration in the SO_3/air was controlled by adjusting the gauge pressure, with a preferred volume ratio of 2-10 vol.%.

8. DNA extraction.

Total genomic DNA samples were extracted using the OMEGA Soil DNA Kit (M5635-02; Omega Bio-Tek, Norcross, GA, USA), following the standard protocol in the official illustration, and stored at −20°C prior to further analyses.

9. 16S rRNA gene amplicon sequencing and sequence analysis.

PCR amplification of the bacterial 16S rRNA genes V3−V4 region was performed using the forward primer 338F and the reverse primer 806R. Sample-specific 7-bp barcodes were incorporated into the primers for multiplex sequencing. The PCR components contained 5 μL of buffer, 0.25 μL of fast Pfu DNA polymerase (5 U/μL), 2 μL (2.5 mM) of dNTPs, 1 μL (10 uM) of each forward and reverse primer, 1 μL of DNA template, and 14.75 μL of ddH_2O. Thermal cycling consisted of initial denaturation at 98°C for 5 min, followed by 25 cycles consisting of denaturation at 98°C for 30 s, annealing at 55°C for 30 s, and extension at 72°C for 45 s, with a final extension of 5 min at 72°C. PCR amplicons were purified with Vazyme VAHTSTM DNA Clean Beads (Vazyme, Nanjing, China) and quantified using the Quant-iT PicoGreen dsDNA Assay Kit (Invitrogen, Carlsbad, CA, USA). After the individual quantification step, amplicons were pooled in equal amounts, and paired-end 2x250 bp sequencing was performed using the Illumina NovaSeq platform with the NovaSeq 6000 SP Reagent Kit (500 cycles). Microbiome bioinformatics were performed with QIIME2 2019.4 with slight modification according to the official tutorials (https://docs.qiime2.org/2019.4/tutorials/).[3]

Instrumentations

1. Mathmatic distribution descriptors.

Polydispersity index (PDI), entropy (S), standard deviation (σ), and calibrated variance (CV) were chosen to describe the product distribution. These descriptors were defined as follows.

$$\mathrm{PDI}=\frac{\mathrm{C\#_w}}{\mathrm{C\#_n}}=\frac{M_n}{M_w} \tag{1-a}$$

$$\mathrm{S}=-\sum \mathrm{P_i}\times\log_2(\mathrm{P_i}) \tag{1-b}$$

$$\sigma=\sqrt{\sum \mathrm{P_i}\cdot(C\#_i-C\#_w)^2} \tag{1-c}$$

$$\mathrm{CV}=\frac{\sigma^2}{C\#_n} \tag{1-d}$$

where $C\#_W$ and $C\#_n$ are the weight and number average carbon numbers; M_n and M_w are number and weight average molecualr weights; P_i is weight fraction of component *i*; $C\#_i$ is the carbon number of component *i*, as revealed by GCMS and APCI-MS.

2.Nuclear Magnetic Resonance (NMR) Spectroscopy.

Quantitative NMR analysis (q-NMR) was performed on a Bruker Avance 400 spectrometer in a deuterated solvent with a relaxation time of 2 s, 64 scans for ^{1}H NMR, and 1024 scans for ^{13}C NMR. Heteronuclear multiple bond correlation (HMBC) experiments were performed on a Bruker Avance II 500 spectrometer in a deuterated solvent with a relaxation time of 2 s, 16 scans, and a digital increment of 400. High-temperature HMBC-NMR (HT-HMBC-NMR) was performed at 50°C to improve surfactant solvation and signal quality.

3. Fourier Transform infrared spectroscopy (FTIR).

FTIR was performed on a Thermo Scientific FTIR Spectrometer (Nicolet iS50) using KBr method with a scan number of 32 and a resolution of 4 cm^{-1}. The KBr background was substrated from the spectra.

4. X-ray Photoelectron Spectroscopy (XPS).

XPS was performed on a Kratos AXIS Ultra DLD spectrometer using a monochromatic Al Kα X-ray source (1486.6 eV) with a 200 μm X-ray beam.

5. Power X-ray Diffraction (PXRD).

PXRD was performed on a Rigaku-Miniflex-600 diffractometer with Cu-Kα radiation (λ = 1.54059 Å) at a working voltage of 40 kV and current of 15 mA. The scanning rate was 5 ° min^{-1} with a range from 5 to 90°.

6. Thermal Gravimetric Analyzer (TGA) and Differential Scanning Calorimetry (DSC).

TGA and DSC were performed on an STA 449 F1 Jupiter thermogravimetric analyzer. Isotherm and ramp experiments were performed under a nitrogen stream of 25 mL min^{-1}. DSC was performed by ramping from 40 to 250℃ at 10 ℃ min^{-1} under a nitrogen stream of 50 mL min^{-1}.

7. Gas chromatography (GC).

GC analysis was performed on a Fuli GC80 equipped with a mass spectrometry detector (Thermo Scientific^{TM}, ISQ 7000, Weily MS library; GC-MS) and a flame ionization detector (GC-FID), using a WAXMS capillary column (30 m long x 250 μm I.D.; film thickness = 0.25 μm). The MSD transfer line temperature was 250℃, and the following operating parameters were used for sample analysis:

Injection Port Temp.	280℃
Purge Valve	5 mL min^{-1}
Injection Volume	1 μL, split 1:20
Column Oven Initial Temp.	50℃
Column Oven Initial Time	0 min
Column Oven Ramp Rate	10 ℃ min^{-1} to 300℃
Column Oven Final Temp.	300℃
Column Oven Final Time	15 min

8. Atmospheric Pressure Chemical Ionization-Mass Spectrometry (APCI-MS).

APCI-MS was performed at a Thermo Scientific Q Exactive system. The mobile phase was MeOH at a flow rate of 200 uL min^{-1}. Samples were dissolved in methanol, and then injected in positive ion mode at 1 uL per dose using a liquid chromatography injector. Data were recorded in profile mode with mass ranges of 50-3000 m/z. ESI-MS performed ionization with the following operating parameters:

Instrument	Thermo scientific Q Exactive
Spray Voltage	5000 V
Capillary Temperature	320.00
Sheath Gas	45.00 Arb
Aux Gas	15.00/20 Arb
Max Spray Current	80.00 μA
Probe Heater Temp	300.00
Ion Source	APCI+ms
Mass range	50-750 and 200-3000 m/z

9. High-temperature gel permeation chromatography (HT-GPC).

HT-GPC was performed using a Agilent PL-GPC50 High Temperature GPC System at 160℃ with sample flow rate of 1.0 mL min^{-1} and reference flow rate of 0.5 mL min^{-1}, equipped with a viscometer detector. The detector was calibrated using standard PP reference. mobile phase was 1,2,4-Trichlorobenzene (TCB,

Fischer Scientific-HPLC Grade). Polymer separation was performed using PL1E10-3120EPA columns 7.5 mm I.D. x 30 cm.

10. Inductively coupled plasma and plasma optical emission spectrometry (ICP-MS and ICP-OES). The digested samples were analyzed using an ICP-MS (Agilent 7800) for comprehensive elemental composition. The instrumental parameters were set as follows.

RF Power	1550 W
Nebulizer Gas Flow Rate	1 L min^{-1}
Cooling Gas Flow Rate	1 L min^{-1}
Auxiliary Gas Flow Rate	1 L min^{-1}

For the analysis of P, S, Br, and I, an ICP-OES (Agilent 5110) was employed and set as follows.

RF Power	1250 W
Nebulizer Gas Flow Rate	0.7 L min^{-1}
Cooling Gas Flow Rate	1 L min^{-1}
Auxiliary Gas Flow Rate	1 L min^{-1}
Plasma Gas Flow Rate	12 L min^{-1}

Characteristic emission wavelengths were selected according to the target elements to avoid spectral interference among multiple elements. To ensure signal stability and reduce interference, acquisition modes and quality analysis strategies were employed as follows.

Acquisition Mode	Collision Cell Mode
Collision Gas	Helium (He), 5 mL min^{-1}
Internal Standard Calibration	Rhodium (Rh) or Rhenium (Re)

Characterization of degradation products

1. Molecular weight and distribution determination by GC and APCI-MS.

The number-average molecular weight (M_n) and weight-average molecular weight (M_w) of the resulting products were estimated using GC (Supplementary Equation 2) and APCI (Supplementary Equation 3) curves.

$$M_n=\frac{\sum m_i}{\sum n_i}=\frac{\sum_1^n KA_i}{\sum_1^n \frac{KA_i}{MW_i}}=\frac{\sum_1^n A_i}{\sum_1^n \frac{A_i}{MW_i}}$$
$$M_w=\frac{\sum m_i \times MW_i}{\sum m_i}=\frac{\sum_1^n KA_i \times MW_i}{\sum_1^n KA_i}=\frac{\sum_1^n A_i \times MW_i}{\sum_1^n A_i} \quad (2)$$

where m_i and n_i are the mass and molar number of a compound; MW_i is the molecular weight of a compound determined by GC-MS or APCI-MS; K represents the mass response factor, which can be assumed to be a constant for alkanes and alkenes according to literature;[4,5] A_i is the peak area from GC-FID.

$$M_n=\frac{\sum m_i}{\sum n_i}=\frac{\sum_1^n C\times I_i \times MW_i}{\sum_1^n C\times I_i}$$
$$M_w=\frac{\sum m_i \times MW_i}{\sum m_i}=\frac{\sum_1^n C\times I_i \times MW_i \times MW_i}{\sum_1^n C\times I_i \times MW_i} \quad (3)$$

where I_i is ion intensity of an ionized species; C represents the MS molar response factor and is assumed to be a constant, due to the minor change of value for long-chain hydrocarbons with different carbon number (0.8-1.1).[6]

The GC-MS column operated at 300°C can accurately detect hydrocarbons below C_{19}. We, therefore,

reconstructed mass distribution profiles of PP-T0-5h and PP-T1-5h directly from GC peak integration areas. For hydrocarbons above C_{20}, APCI-MS delivered better accuracy than GC. Therefore, mass distribution profiles of PP-T2-5h and -ST-5h combined GC-MS (C_{15}-C_{19}; Supplementary Figure 8) and APCI-MS (above C_{20}; Supplementary Figure 9). As APCI-MS functions as a molar detector, mass contributions were calculated by multiplying peak intensities by molar masses. PP-NT-5h was dominated by components above C_{19} and was analyzed only via APCI-MS.

2. Alkenyl concentration characterization.

The concentration of alkenyl groups in the PP degradation products was evaluated using external reference method by the following procedure.

a. ***Calibration of deuterated solvents:*** A GC glass vail was pre-weighted (m_0). $CDCl_3$ (~ 2 mL) was carefully injected into the pre-weighted GC glass vail (m_1). The mass of $CDCl_3$ was determined as m_1-m_0. Acetonitrile (~ 20 mg), serving as the external reference, was then injected into the vail and weighted (m_2). The amount of acetonitrile was determined as m_2-m_1. The mixture was swirled to ensure thorough mixing. The mixture was immediately characterized using NMR spectroscopy. The determined concentration of $CHCl_3$ in the $CDCl_3$ was 2.333 mg g^{-1}.

b. Alkenyl concentration determination: Precise masses of both the degraded substances and $CDCl_3$ were measured and combined in clean GC vials. The vials were sealed with PTFE caps and swirled for dissolution. To prevent potential loss from evaporation, the mixtures were promptly analyzed using NMR. The alkenyl group concentrations were then calculated through Supplementary Equation 4 based on the NMR data

$$c_{C=C}=\frac{m_{solvent}f}{M_{solvent}}\times\frac{n_{ref-H}}{n_{ene-H}}\times r\times\frac{1}{m} \quad (4)$$

where $c_{C=C}$ is the concentration of alkenyl groups in the products (mol g^{-1}); $m_{solvent}$ is the mass of the solvent; M_{CHCl3} is the molar mass of chloroform; f is the fraction of $CHCl_3$ in $CDCl_3$ and equal to 2.333 mg g^{-1}; n_{ref-H} is the number of reference H on a solvent molecule; r is the NMR peak ratio of alkenyl to $CHCl_3$; n_{ene-H} is the number of H on alkenyl groups (n_{ene-H} = 2); m is the mass of the sample.

3. Yield determination.

The yields of gas and liquid were measured directly using analytical balances. For laboratory-scale reactions in fractionated reactors, the products in T0, T1, T2, and ST were collected using pre-weighted containers (gas bag, syringes, vials; m_3). The final masses (m_4) were recorded and the yields of each samples were determined by the difference (m_4-m_3). Any remaining material was attributed to residues. The yield of PP-NT-5h was determined using the hexanes refluxing method described in previous work.[1] The yield of PP-STW-6h was similarly determined using a platform balance. For scale-up experiments, the solid residues were collected from the reactor and measured using the platform balance. Due to their large volume, gas products were not collected, and were categorized as uncollected content.

4. Elemental analysis.

The elemental composition of the degradation products was analyzed using ICP-MS and ICP-OES. **Digestion:** microwave digestion was conducted using a TANK40 model microwave digestion instruments (Xinyi) in a PTFE vessel at 180°C for 30 min. Specifically, for comprehensive elements analysis and Ca, Mg, P, and S analysis, a 0.5 mL aliquot of the sample was placed in Teflon vessel, followed by the addition of 10 mL of aqua regia to promote oxidative decomposition. For Br and I analysis, 0.5 mL of the sample was placed in a PTFE microwave digestion vessel, and a solution of NaOH (10 mL, 1 M) was added to promote decomposition. After digestion, the sample vessel was allowed to cool naturally to prevent sample loss. **Analysis**: the digested sample was dissolved and homogenized in ultrapure water (25 mL) and delivered to ICP-MS for analysis of comprehensive elements, Ca, and Mg, or to ICP-OES for the analysis of P, S, Br, and

I. The concentration of Ca, Mg, P, S, Br, and I were quantified using standard materials (Tanmo Quality Control: S, Ca, Mg, P, and Br; National Nonferrous Metals: I) and the standard curve method (Supplementary Figure 24 and Supplementary Table 12). Other elements were quantified by parallel comprehensive analysis. Background signals and polyatomic ion interference were minimized using the Agilent ICP analysis interface.

5. Purity estimation of real-life PP wastes.

Following China's national and ASTM standards,[7,8] we manually sorted real-life plastic waste to determine their compositions. Waste plastics were evenly spread on a 1-meter diameter circular zone (sorting area), in a clean 1 m^2 platform to prevent contamination. Operators categorized materials by appearance, texture, plastic number, and other properties to separate plastics from impurities. Separated components were classified by type, weighed using a platform balance (±1 g accuracy), and purity of plastic waste was calculated as the plastic mass fraction.

Characterization of PP upcycling products

1. Structural evolution by ^{1}H NMR.

To monitor structural evolution, a reaction was conducted in $CDCl_3$ within an acetone/dry ice bath at −78°C. After sulfonation, the reaction mixture was transferred into an NMR tube using a cold syringe. The tube was then placed in a Dewar flask containing acetone/dry ice for transport to the NMR spectrometer. A ^{1}H NMR spectrum was recorded immediately, followed by subsequent analysis after 1 minute and 1 h to observe the structural evolution.

2. Detergency performance of surfactants.

The detergency was measured by referencing ISO 4312:1989 standard with modifications for practicality. In a typical test, 1 g of the surfactant was dissolved in 500 mL hard water (250 mg kg^{-1}) to prepare the washing solution. Testing cloths with sebum and protein stains (2 × 2 cm^2) were prepared according to the method in the standard. Rather than using a washing machine, the washing solution (250 mL) and testing cloth were put in a beaker and stirred for two hours (300 rpm, 3 cm PTFE stirrer) at 35°C. Then, the testing cloth was collected and dried at 100°C on a hot plate for 1 h, and the whiteness was characterized by a whiteness meter.

3. Foaming height measurements.

The foaming height was measured using the Ross-Miles method.[9] Specifically, 2.5 g of the sample was dissolved in hard water (1 L, 150 mg kg^{-1}) to prepare the experimental solution. This solution (~50 mL) was warmed up to 40±0.5°C and poured into a foam-meter, a jacketed glass tube. The solution level was calibrated to 0 cm. The testing environment was maintained at 40±0.5°C by pumping warm water (~ 40°C) into the jacket. Another 200.0 mL of testing solution was transferred to a pipette and placed at the top of the foam-meter. At the midpoint of the foam-meter, 200.0 mL of surfactant solution was released, inducing foaming. After 5 min, the height of the foam was recorded.

4. Wettability Measurement.

The wettability of surfactants was determined using the cloth disc method, following a modified procedure of ISO 8022:1990. The aqueous surfactant solutions were prepared by dissolving 0.1 g surfactants in deionized water (100 mL), and then poured into graduated cylinders. Canvas cloth of uniform thickness was cut into uniform discs (0.5 cm × 0.5 cm). A stopwatch was used to record the time taken for each disc to sink to the bottom of the cylinder.

5. Biodegradation.

The biodegradation test was conducted using a modified JIS K 3363-1990 method.[10] The active sludge dispersion was replaced with natural water, collected from Gaoguan River (Supplementary Figure 30).

Specifically, nutrient base solution (1 L), including ammonium chloride (3.0 g), dipotassium hydrogen phosphate (1.0 g), magnesium sulfate (0.25 g), potassium chloride (0.25 g), iron (II) sulfate (0.002 g), and yeast extract (0.3 g) in saline water (1 L) was mixed with natural water (4 L). Surfactant sample (7.5 mg) was dissolved in nutrient base solution (125 mL) and mixed with 125 mL of the natural water. The mixture was stored in a conical flask and placed on a shaker (25 ± 3°C, 100 rpm). The degradation solution was sampled at 30 minutes (initial reference point), 3 days, 5 days, and 7 days.

To determine the surfactant concentration, a degradation solution (3.00 mL) was transferred into a separatory funnel and diluted to 100 mL with DI water. Methylene blue solution (0.003 wt.%, 25.0 mL) and chloroform (15.0 mL) were added to the water solution. The mixture was shaken to ensure efficient extraction and then allowed to stand for phase separation. The chloroform layer (bottom layer) was collected and transferred to a flask. The extraction was repeated by adding fresh chloroform until the chloroform layer became colorless. The chloroform extracts were then combined with sodium dihydrogen phosphate solution (50 mL, 0.4 M). The chloroform layer was transferred to a 100 mL volumetric flask and the extraction was repeated until the chloroform layer was colorless. Finally, the chloroform extract was diluted to 100 mL with fresh chloroform. The surfactant concentration was then determined using UV-vis spectrophotometry, measuring absorbance at 650 nm (Supplementary Figure 29). The absorbance of the samples was compared to the initial absorbance, and the ratio of the two was used to calculate the extent of surfactant consumption

6. Net surfactant content.

To evaluate the product net content, the contents of water and other volatiles in the PP-STW-6h-S was determined using stepwise isothermic experiment. The floating solid was collected by skimming and loaded into a Pt pan. Under stepwise heating (30, 40, and 50°C, 5 h each), the sample weight eventually stabilized near 92 wt.% (Supplementary Figure 16c).

Fractionated degradation modelling

1. $C\#_n$ profile modeling.

The temperature profile of fractions under steady-state conditions is typically approximated as linear, based on the classical McCabe-Thiele theory. The simplification also holds regionally in a column for some practical distillation of non-ideal binary mixtures (Supplementary Equation 5).[11] In our study, we found that the linear assumption is valid, as demonstrated by the IR camera image of the reactor's external surface (Figure 2a and Supplementary Figure 13). Internal liquid temperatures, measured by thermocouples, also showcased good linearity (Figure 2a). Thus, we adopted the linear temperature profile as follows:

$$T=T_b+\frac{T_\Delta}{H}\times h \tag{5}$$

where T is the temperature at height h; T_b is the temperature at the bottom; T_Δ is the temperature difference between the tower top and bottom; H is the total height of a tower; h is the height at which the temperature was being measured.

With the application of VLE and heat transfer principles, the plate temperature approaches the boiling point at equilibrium and ideal thermal insulation, as described by the Clausius-Clapeyron equation. Given this, we established a relationship between plate temperature and average molecular weight by making the following assumptions:

a. **Size-dependence:** the boiling point of the feedstock depends on the molecular weight;
b. **Uniform feedstock:** assumed to consist of hydrocarbons with similar molecular structures;
c. **Even heat supply:** uniform temperature distribution across the column's cross-section.

Given these assumptions, the term T can be replaced by the boiling point T_{bp}, yielding Supplementary Equation 6.

$$T_{bp}=T_b+\frac{T_\Delta}{H}\times h \tag{6}$$

This was then incorporated into the empirical relationship, resulting in:

$$T_{\mathrm{bp}}=\mathrm{A}\times e^{-\frac{M_\mathrm{n}}{\mathrm{B}}}+\mathrm{C} \tag{7}$$

$$\mathrm{A}\times e^{-\frac{M_\mathrm{n}}{\mathrm{B}}}=\mathrm{T_b}+\frac{T_\Delta}{\mathrm{H}}\times \mathrm{h}-\mathrm{C} \tag{8}$$

where A, B, and C are empirical constants from nonlinear fitting, and equal to -692.9, 219.5, and 536, respectively.

With a linear temperature profile, the height h can be redefined as a function of temperature T (Supplementary Equation 9).

$$\mathrm{h}=(1-\frac{T}{T_\Delta})H \tag{9}$$

Reorganizing this relationship, we derived the M_n profile as follows.

$$e^{-\frac{M_\mathrm{n}}{\mathrm{B}}}=\frac{T}{\mathrm{A}}+\frac{T_\mathrm{b}-T_\Delta-\mathrm{C}}{\mathrm{A}} \tag{10}$$

Similarly, for the C$\#_\mathrm{n}$ profile:

$$e^{-\frac{M_\mathrm{n}}{14\mathrm{B}}}=\frac{T}{\mathrm{A}}+\frac{T_\mathrm{b}-T_\Delta-\mathrm{C}}{\mathrm{A}} \tag{11}$$

As discussed earlier, heat loss was neglected. In practice, heat loss is inevitable. Hence, an empirical correction factor k (k = 1.947) was introduced to account for real-world heat losses in fractionated reactor, resulting in practical correlations for M_n or C$\#_\mathrm{n}$ with T (Supplementary Equations 12 - 14).

$$e^{-\frac{M_\mathrm{n}}{\mathrm{B}}}=\frac{T}{\mathrm{A}k}+\frac{T_\mathrm{b}-T_\Delta-\mathrm{C}}{\mathrm{A}k} \tag{12}$$

$$e^{-\frac{M_\mathrm{n}}{14\mathrm{B}}}=\frac{T}{\mathrm{A}k}+\frac{T_\mathrm{b}-T_\Delta-\mathrm{C}}{\mathrm{A}k} \tag{13}$$

The empirical correction factor k was used to correct the ASPEN simulation which was conducted with ideal thermal insulation assumption.

2. Calculation of separation efficiency.

The theoretical separation efficiency (η) of the fractionated reactors was estimated using the corrected O'Connell method.[12]

$$\mu=0.503\times\alpha^{-0.08}\times\mu_\mathrm{L}^{-0.226} \tag{14}$$

where α denotes relative volatility and μ_L is dynamic viscosity (mPa-s; Supplementary Table 7).

The equilibrium vapor pressure of hydrocarbons at different temperatures was calculated using group additive method (Supplementary Equation 15):[13]

$$\ln(P)=\sum \mathrm{a_i}+\sum \mathrm{b_i}/\mathrm{T}+\sum \mathrm{c_i}\times\ln T-\sum \mathrm{d_i}\times T-\ln(M_\mathrm{n}) \tag{15}$$

where P represents the vapor pressure, a_i, b_i, c_i, and d_i are group additive values. For simplicity, all hydrocarbons were assumed to be linear α-alkenes with varying carbon numbers. The required group additive values were tabulated in Supplementary Table 8, while additional values can be found in the Table 2 of the referenced source.[13]

For simplicity, the hydrocarbon vapor was assumed to be an ideal gas. The relative volatility (α) was estimated as follows.

$$\alpha=\frac{P_A}{P_B} \tag{16}$$

where P_A and P_B were vapor pressure of the light and heavy components, respectively.

Since PP-T0-5h, PP-T1-5h, PP-T2-5h, and PP-ST-5h represent mixtures of alkenes with varying chain

lengths, a weighted average method was used to calculate P_A. For PP-T0-5h and PP-T1-5h, P_B was chosen as the vapor pressure of the lightest hydrocarbon in the lower tray (C6 for PP-T0-5h and C15 for PP-T1-5h) and was taken as the heaviest component in the mixture (C30 for PP-T2-5h and C60 for PP-ST-5h at 400°C). The results were tabulated in Supplementary Tables 9-10.

3. Chain scission kinetics.

The initial molecular weight of laboratory-grade PP was determined by HT-GPC (Supplementary Figure 15a). To determine the chain scission rates, laboratory-grade PP powder (< 5 mg) was added into a small thin Schlenk flask (78 mL, diameter 5.3 cm, thickness 1.02 mm). The flask was degassed and refilled with Ar three times to evacuate oxygen. Constant Ar flow was supplied to the reactor from the side-tube through a flexible long needle when heating the reactor to the target temperature (360 or 400°C; ~50 °C min^{-1}). The temperature was monitored with a thermocouple, placed in the center of the reactor. After ~10 minutes, the temperature reached and stabilized near the target temperature. The long needle and thermocouple was removed and the Teflon cap was closed. After a few hours, the reaction was halted by immersing the flask into water. Deuterated solvent (10 mL) was injected into the flask to dissolve the products for APCI-MS characterization (Supplementary Figures 15b, 15c, and Supplementary Table 3). To avoid interruption by polymer melting and side reactions, linear regions (0.5 - 4 h) were fitted by random scission model (Supplementary Equation 17 and Supplementary Figure 15d).[14] Using Arrhenius equation, ln(A) and activation energy (E_a) were predicted to be 19.7 h^{-1} and 134.4 kJ mol^{-1}.

$$\ln(1-\frac{1}{P_{\mathrm{n}}(t)})=\ln(1-\frac{1}{P_n(0)})-kt \tag{17}$$

4. Biodegradation kinetics of surfactants.

Due to the low concentration of substrate in experiments, validating assumptions of no microbial growth and low substrate concentrations were made for the biodegradation of surfactants (Supplementary Equation 18).[15] The concentrations were determined using UV-*vis* spectrometer (Supplementary Figure 31).

$$-\frac{dS}{dt}=k_1S$$
$$S=S_0exp(-k_1t) \tag{18}$$

where S represents the substrate concentration at time t; S_0 is the initial substrate concentration; k_1 is the first-order rate constant.

Fractionated degradation simulation by ASPEN

1.ASPEN HYSYS simulation general flow and procedure.

ASPEN HYSYS is a widely used process simulation software developed by ASPEN Technology, primarily for the design, optimization, and analysis of chemical processes. In this work, we used ASPEN HYSYS to simulate the fractionated degradation and upcycling of polymers. To account for the absence of standardized molecular formulas for the polymer components in the ASPEN, the Polymer Degradation Database was employed and virtual components were established. Missing kinetic parameters were evaluated experimentally (Supplementary Figures 15 - 16).

In general, the process flow diagram (PFD) should be defined to perform ASPEN simulations, by selecting the appropriate components from the software database, including reactors, distillation columns, heat exchangers, and other pre-built unit operation modules (Supplementary Figure 17). The operating conditions and feedstock properties should be specified (Supplementary Tables 4-6). Once the PFD has been defined, the simulation should be initiated to obtain the results.

The simulation followed the procedure below.

Step 1: Define components and calculation methods. In ASPEN HYSYS, the simulation began by

defining the components involved in the fractionated degradation, including the raw material (polyolefin) and products. The polymer properties were either defined manually (Supplementary Table 6) or retrieved from the ASPEN polymer database.

Step 2: Define the process flow diagram. The PFD was defined by selecting the relevant unit operations from the ASPEN HYSYS database, including reactors, feed streams, and product streams (Supplementary Figure 17). The operating conditions, such as temperature, pressure, and flow rate, were specified for each unit operation (Supplementary Tables 4-6).

Step 3: Specify the reaction kinetics. Due to incomplete physical property data for polyolefins and their degradation reactions, virtual components were defined for simulation. The necessary properties were either experimentally determined or referenced from the literature (Supplementary Table 4 and Supplementary Figure 16). Next, the PP degradation reaction (PP→C3(alkene)+C4(alkene)+⋯ ⋯) and the corresponding rate law (Supplementary Equation 17) were entered into the simulation.

Step 4: Define the reactor properties and feed streams. The reactor properties, including reactor type, size, and configuration, were specified. ASPEN HYSYS offerred several reactor models, including batch reactor (BatchR), plug flow reactor, and fluidized bed reactor. Since the fractionated reactor was not a standard module, it was modeled as a BatchR in series with a column. The volume (equal length, width, and height) of the reactor and the column dimensions were specified (Supplementary Table 4). The kinetic parameters of PP under fractionated degradation conditions (isothermic heating and ambient pressure at ~ 1 atm) were determined using random scission model and utilized for ASPEN reactor setup (E_a, A, and reaction order). Feed streams were defined by their composition, flow rate, and temperature, as shown in Supplementary Tables 4-6. Particularly, given the absence of industrial referential efficiency data for fractionated degradation, we assumed η = 100%, aligning with near-optimal industrial operation of typical oil refinery (70 - 90%).

Step 5: Run the Simulation. The simulation was run once the process flow sheet and reactor properties were defined. ASPEN HYSYS solved the mass and heat balances and applied the specified reaction kinetics. The reactor performance was calculated, including product composition and yield. These results were used to evaluate and optimize the reactor design by adjusting operating conditions or changing the reactor configuration as needed.

Step 6: Analyze the Results. After running the simulation, the results were analyzed using the built-in tools in ASPEN HYSYS. These included graphical representations, tables, and detailed reports summarizing key performance indicators, such as product yield, temperature profiles, and pressure drops.

2. Simulation of feedstock and degradation.

The reactor was modeled as a BatchR. The reactor and separation units were configured sequentially: the BatchR module was used for the reaction step, while the Refinery System Distillation Tower module was used for the separation step. The physical properties (cutting temperature, density, kinematic viscosity) and cutting distribution of degradation products were experimentally measured through a cutting experiment of PP-ST-5h (Supplementary Table 6), with four cutting points, 10%, 30%, 50%, and 60%. Physical properties of each fraction were measured.

A virtual component of PP plastic was set and assigned the following properties:

- Molecular weight: 40,000 g mol^{-1};
- Density: 920 kg m^{-3};
- Diameter: 10 mm;
- Sphericity: 1.00.

Using the known experimental conditions and properties, the “Estimate Unknown Properties” function was applied, with Peng-Robinson as the selected equation of state (EOS), providing the minimum and

maximum temperatures corresponding to the Antoine vapor pressure.

In the Oil Manager Interface, the following data setup was configured:

- Select “Used” for overall physical properties;
- Select True Boiling Point (TBP) distillation and Engler Distillation, ASTM D86
- Choose “TBP distillation curve” for the oil data type;
- Select “Dependent Variables” for the density and viscosity curves;
- Set “Normal pressure” for the TBP distillation conditions.

The degradation product distributions were entered as percentages (10%, 30%, 50%, and 60%), with the corresponding physical properties input as detailed in Supplementary Figure 6.

In the oil blending interface, the following parameters were configured:

- Flow unit: Mass;
- Cutting range: User-defined.

The simulation experiment for PP is conducted with user-defined cutting-end temperatures. The number of cuts was set to specified values (*e.g.*, 1, 3, 5, 7, etc., as shown in Supplementary Table 7). The simulation generates molecular weights for each specified number of cuts, as well as the temperature profile of column (Supplementary Table 7). As the simulation assumed perfect thermal insulation, a heat loss factor k (Supplementary Figure 14) was applied for plotting C# with temperature.

Technoeconomic analysis (TEA) and life cycle analysis (LCA)

1. Water evaporation estimation.

Water evaporation was calculated using solar drying and spray drying. Solar drying incorporated both natural evaporation ($E_{m,emp}$) and solar-driven evaporation (E_{solar}), which were estimated using empirical equations (Supplementary Equation 19), and calculated as $E_{m,emp}+E_{m,emp}$.[16]

$$E_{m,emp}=10^{-5}(28+78.4v_G)\frac{(p_{v,sat,f}-p_{v,G})}{r_w} \quad (19)$$

where v_G represents the gas velocity; $p_{v,sat,f}$ and $p_{v,G}$ are the saturated vapor pressure and vapor pressure in the gas phase, respectively; r_w is the specific gas resistance of water.

$$E_{solar}=\frac{I\cdot A_s}{L} \quad (20)$$

where I is the solar radiation intensity; A_s is the exposed surface area of water; L is the latent heat of water vaporization. The total water evaporation rate in this method is the combination of natural evaporation and solar-driven evaporation.

The energy demand for spray drying was ~ 11.6 kJ g^{-1}, cited from a reference regarding pilot-scale spray drying energy consumption.[17]

2. Simulation of upcycling process.

An ASPEN simulation of the PP waste upcycling to surfactants was conducted using the PFD (Figure 4a and Supplementary Figure 17b). The simulation process was based on the following assumptions:

a. Production capacity: 10,000 tons per annum;

b. Three shifts per day and 8,000 working hours were assumed;

c. Degradation reaction conditions: electrical heating, 400°C working temperature, 1 atm working pressure in N_2, and a reaction time of 8 h (an extra 2 h was needed for the first batch for heating up). The product chain length and distribution were not specified for simplicity.

d. Hydrocarbon yield: The yields of ~ 87 wt.% in the scale-up experiment (Figure 3c) was used in the design for material flow (Figure 5 and Supplementary Table 5). The $C_{C=C}$ of the hydrocarbon is assumed

to be 4.5 mmol/g, according to the characterization of PP-STW-6h (Supplementary Table 1)

e. Upcycling reaction conditions: **Sulfonation and hydrolysis.** The hydrocarbons were transferred to a drop-film reactor equipped with a stirring system. The operation conditions and procedure followed a recommended procedure in the sulfonation industry.[2] Excess SO_3 was utilized and converted into Na_2SO_4. **Drying.** After sulfonation and hydrolysis, the products were collected by skimming and dried in air for 12 h , resulting in net content > 95% (~ 30°C, Supplementary Figure 16c).

f. Upcycling conversion of α-olefins to surfactant was assumed to be 99% (Figure 5a), generating ~13 t surfactant (residue water was ignored) from 10 t of plastic wastes.

3.Technoeconomic analysis (TEA).

The TEA was based on the block flowchart, PFD, and the ASPEN simulation (Supplementary Tables 13-15, Figures 14-17). The material balance calculations were based on the annual capacity of 10,000 tons of PP waste (Figure 5a). The TEA was evaluated using a similar method to our previous reports, including calculations of the following key parameters.[18]

a. Capital investment: An industrial setup to upcycle 10,000-ton PP was designed and evaluated using ASPEN (Supplementary Tables 13-15). Based on the PFD (Supplementary Figures 17 and 32) and equipment cost (Supplementary Table 13), we estimated the fixed-capital and total capital investment using the method of Percentage of Delivered-Equipment Cost.[19]

b. Product cost and revenue: Details of equipment cost and manufacturing costs were given in Supplementary Table 13. The raw material cost and other direct production costs were estimated as a ratio of the total product cost or the labor cost.[19] Details of the fixed charges and costs assumptions were given in Supplementary Table 13. Normally the fixed charges take 5%-15% of the total product cost. Gross earnings are calculated by total revenue minus total product cost. The total annual revenue from product sales is the sum of the unit price of each product multiplied by its rate of sales, that is, $/yr = ∑(sales of product, ton/yr)(product sales price, $/ton).

c. Prices: The raw material costs and product selling were obtained by direct quotation and secondary market research (Supplementary Table 15). Particularly, the prices of PP wastes and SO_3 were obtained by direct quoting from the circular economy industrial park, Jieshou Anhui, China on July 23, 2024. Quoted prices were converted at a CNY/USD exchange rate of 7; the price of SO_3 was obtained by quoting Xingzhilian Ltd. Zibo, Shandong, China.

d. Profitability: The profitability was evaluated based on return on investment (ROI), payback period (PBP), net present value (NPV), and internal rate of return (IRR). ROI and PBP do not consider the time value of money, but NPV and IRR do. NPV combined with IRR is recommended for making economic decisions. The profitability and other TEA parameters were tabulated in Supplementary Table 15.

ROI and PBP are defined as follows,

$$\text{ROI}=\frac{\text{annual net profit}}{\text{total capital investment}} \tag{21}$$

$$\text{PBP}=\frac{\text{fixed capital investment}}{\text{average annual cash flow}} \tag{22}$$

Depreciation was calculated using the straight-line method (Supplementary Equation 23), assuming the property value decreases linearly with time over the recovery period.

$$\text{Annual depreciation}=\frac{\text{OPI}}{\text{LRP}} \tag{23}$$

where OPI is the original property investment at the start of the recovery period, LRP is the length of the straight-line recovery period.

NPV is the total of the present value of all cash flows minus the present value of all capital investments (Supplementary Equation 24). NPV is calculated based on nominal net cash flow A_i at year i; d is the discount rate; j is the lifetime; and TCI refers to total capital investment, defined as follows.

$$NPV=\sum_{i=1}^{j}\frac{A_i}{(1+d)^i}-TCI \quad (24)$$

IRR is defined as any discount rate that results in an NPV of zero. Thus, it is determined by setting NPV = 0 in Supplementary Equation 24 and solving for the discount rate (d) that satisfies the resulting relation.

4. Life cycle assessment of greenhouse gases emissions.

The process flow and system boundary of waste plastic recycling and surfactant production were displayed in Supplementary Figure 32, based on the energy consumption analysis by ASPEN (Supplementary Table 15) and emission factors in China. Four principal processes, from waste collection and transportation, to fractionated degradation, upcycling, and post-treatment (hydrolysis and drying), have been investigated. In this study, the functional unit was defined as 1 ton of waste PP entering the system of recycling. The life cycle greenhouse gas (GHG) emissions can be computed by the following equation:

$$CE=\sum(EN_{i,j}\times ec_j+FA_{i,j}\times ef_j-FS_{i,j}\times ef_j) \quad (25)$$

where CE is the GHGs emissions to recycle 1 ton of waste plastic; $EN_{i,j}$ is the energy j consumed in process i; ec_j is the GHGs emission factor of the consumption of energy j; $FA_{i,j}$ is the material j consumed in process i; ef_j is the emission factor of the production of material j; $FS_{i,j}$ is the material j generated in process i; ef_j is the emission factor of the primary production of material j; $FS_{i,j}\times ef_j$ represents the potential GHGs reduction benefits by substituting recycled material for primary material.

The material inventory throughout the processes was depicted as below:

a. Collection and transportation: The waste plastic was assumed to be transported by a medium truck, with an average shipping distance of 100 km. The emission factor of truck shipping is 0.147 $kgCO_2e\ (t\ km)^{-1}$ (Supplementary Table 15).

b. Fractionated degradation: With 1 ton of waste plastic input, the reaction process produced 870 kg of oil, 120 kg of coke, and 10 kg of reaction gas. Two reaction scenarios (B and D) have been assessed. In the D scenario, the total energy consumption was ~460 kWh of electricity per ton. In the alternative B scenario, the energy use can be saved to ~154 kWh electricity. The coke output can substitute coke from coal, and thus provide a carbon offset. The offset credit was calculated according to the emission factor of coke production from coal. The emission factors of energy and materials can be seen in Supplementary Tables 14 and 15. In all scenarios, the product chain length and distribution were not specified for simplicity.

c. Upcycling: The material inputs of the upcycling process were 870 kg oil, 191 kg NaOH, and 365 kg SO_3. The major output was 1,300 kg of surfactant. The electricity consumption to upcycle this amount of oil was 86.9 kWh and assumed unchanged for B and D scenarios. The surfactant can replace those from conventional production and create a carbon offset benefit. The emission factor of NaOH, SO_3, and conventional surfactant are presented in Supplementary Table 15.

d. Post-treatment (hydrolysis and drying): In the post-treatment, the water residue was dried to recover surfactant products (Figure 5a). Two drying approaches, spray drying (-P) vs. solar drying (-SD), can be applied. In the -SD scenarios, electricity demand was ignored as solar light was the major energy source needed per functional unit of material input. For -P scenarios, the electricity use was 238 kWh.

Supplementary discussion and overview

Field research of PP waste supply and processing work flow

A field research was conducted in a major plastic circulation industry park in Jieshou, China at July 23, 2024. To learn the status-of-quo of plastic supply and processing work flow, the field research was conducted through non-participant observation and interview to the park supervisors and enterprise owners. We visited 5 companies in the park, including two large enterprises (100k ton per year), one specialized for PE and PP, and one for general plastics. The interviewing questions included (1) what product do you produce, (2) where do you obtain waste plastic, and (3) how do you convert the plastic waste to product; (4) what is the market price of PP wastes.

Jieshou is a major contributor of plastic waste circulation in China. According to China Xinhua News (https://www.ah.chinanews.com.cn/news/2023/0211/312924.shtml), an official media of China, the annual capacity of Jieshou in 2024 was 2.6 million metric tons, corresponding to ~15% of plastic wastes recycled in China, and ~8% of the world. Therefore, the operation situation in Jieshou is representative to the plastic circulation system in China, and partially representative of the world.

A potential general work flow for real-life PP waste is recovered based on the field research (Supplementary Figure 2). The initial pre-sorting is the critical step in the flow. The pre-sorting by plastic characteristics (*e.g.*, plastic labels, transparency, tactility, initial functions, etc.) helps skillful workers to determine the plastic type. In a company specialized for polyolefins, plastic waste are ordered by plastic types and colors (Supplementary Figure 2a; *e.g.,* hard bottles, caps, shopping bags, woven bags, etc.) to improve the purity and color uniformity of their products. The sorted wastes can be baled for low-quality regenerated plastics (*e.g.*, disposable product, counterweight), or delivered for cleaning and refinement for improved waste quality. Sink-flow system is generally equipped to remove the heavy components (stones, sand, ashes, heavy polymers). The purified plastic wastes are dried for sell, or sorted again mechanically (AI-assisted and spectroscopic systems) for higher purity (Supplementary Figure 22,W1, ~ 99 wt. %), typically utilized for food-grade materials.

The product value is determined by the waste purity. A rough value margin of 128-400 \$/ton was quoted for beaconing the typical pricing of polyolefin wastes after primary sorting (mechanical sorted wastes are not included; Supplementary Figure 2). Although, the actual prices of each were not revealed, it possibly elevated after each purifications. To be conservative, the highest price of 400 \$/ton was utilized in TEA.

Disadvantage of the non-fraction reactors

The typical reactor for thermal gradient degradation is a non-fraction reactor (Supplementary Figure 4). It was designed by Zhen Xu based on the reactive separation principle to fully capture degradation products and rigorously analyze degradation mechanisms.[1] By adjusting the temperature gradient, particularly the outlet temperature, the average molecular weight or average chain length of the hydrocarbons can be controlled. However, this approach is ineffective and inefficient for differentiating sizes and manipulating distribution, as demonstrated in previous studies and our characterization of PP-NT-5h (Supplementary Figures 4 and 9).[20]

An reason of the ineffective size differentiation and separation is the significant entrainment, heavy hydrocarbons and oligomers are observed in HT-GPC and APCI-MS analyses in the literature.[1,20] Entrainment is a phenomenon where liquid droplets are carried along with the vapor phase as it ascends through the distillation column. This can occur due to the high velocity of the vapor or turbulence in the column, effectively pushing liquid droplets upwards to the collector. Entrainment phenomena reduces

separation efficiency and induce contamination, which, in the context of PP and PE degradation, leads to broad CLD.

Inhibiting entrainment in NT reactors is challenging, as revealed by the Souders-Brown equations (Supplementary Equation 26).[11]

$$\frac{\pi g d^3}{6}(\rho_l - \rho_g) = \frac{k_s \rho_l \pi d^2}{4} v^2 \qquad (26)$$

where d is the diameter of the droplet; ρ_l and ρ_g are the densities of the liquid and gas, respectively; k_s is resistance coefficient that related to the droplet shape; v is the velocity of surrounding medium. The criteria of the minimum medium velocity for floating a droplet with specific shape is as follows.

$$v \geq \sqrt{\frac{2gd}{3k}} \times \sqrt{\frac{\rho_l - \rho_g}{\rho_g}} \qquad (27)$$

Experimental results show that the medium velocity in a non-fractionating reactor meets the conditions that promote entrainment (Supplementary Equation 27). To counteract this, altering the densities of the liquid and gas, or adjusting droplet size and shape are not feasible to operate. Therefore, the most practical approach to controlling entrainment is adjusting the velocity. Although reducing the degradation temperature can lower the medium velocity, this would decrease process efficiency and increase operational costs as well. To mitigate entrainment, a de-entrainment plate (preferred with small sieve pore size) should be introduced into the reactor, which effectively induces fractionation and transforms a non-fractionating reactor into a fractionating one.

Fractionated degradation process and advantages

1. Reaction Process. Fractionated degradation may progress through three stages: initiation, equilibrium, and termination, evidenced by chain scission kinetic curves (Supplementary Figure 15d). During initiation, reaction kinetics were constrained by polymer melting rates but subsequently accelerated. This acceleration induced pressure buildup, rapid vapor flow, and entrainment phenomena (Supplementary Figure 5d), producing broad chain-length distributions at the reaction start (PP-T1-1h and PP-T2-1h; Supplementary Figures 8-9).

In the equilibrium stage, VLE and re-degradation serve as the fundamental processes narrowing distribution, which, macroscopically, can be tuned by adjusting F#.

Vapor-Liquid Equilibrium (VLE): molecular redistribution driven by VLE is fundamental for narrowing CLDs of hydrocarbons. The VLE governs the bidirectional, thermodynamic separation of the hydrocarbon mixture on a tray. During operation, lighter hydrocarbons preferentially vaporize from the liquid phase due to the low vaporization enthalpies, when heavier hydrocarbons in the vapor phase undergo condensation, migrating downward to lower fractions. Each tray, thus, continuously strips heavy components from ascending vapor and light components from descending liquid, progressively sharpening molecular weight distributions within individual fractions. The process establishes well-defined temperature and $C\#_n$ gradient along the mass transfer pathway (Figure 3b).

Re-degradation: selective breakdown of heavy fractions, specifically oligomers, long-chain hydrocarbons, and macromolecular byproducts is critical for reducing hydrocarbon chain lengths and lowering the molecular weight upper boundary. Re-degradation is intrinsically linked to VLE driven descending flows. The efficiency of re-degradation was quantified by the scission number s ($s = M_0/M_t$, where M_0 represents initial molecular weight and M_t denotes post-scission molecular weight).[21] Fractionated systems (*e.g.*, three-fraction and single-fraction reactors) enable recirculation of heavy hydrocarbons to

degradation zones. In contrast, non-fractionated configurations accumulate ultra-heavy species due to inefficient or suppressed VLE descending flows (Supplementary Table 1).[1,20]

Fractionation: Adjusting F# is the straightforward strategy to enhance VLE efficiency. Each additional tray establishes a discrete VLE stage, cumulatively narrowing CLDs to yield progressively sharper, more discrete $C\#_n$ bands across fractions, as evidenced by the logarithmic correlation across reactors (Supplementary Figure 18).

Process termination may occur through reactant depletion and competitive side reactions. During termination, diminished pressure impedes mass transfer. Constant reflux within high-temperature zones preferentially drives side reactions (Supplementary Figure 26) rather than chain scission events.

2. Advantage of fractionated degradation over direct distillation.

a. Selective preparation of PP-derived α-olefins with tunable and narrow CLD at high yield. Fractional degradation synthesizes a tailored fraction of specific hydrocarbons, whereas direct distillation partitions an existing mixture for useful portions. In our three-fraction reactor system, C_3-C_7 (gaseous), C_6-C_{15} (liquid), and C_{15}-C_{30} (liquid) α-olefins were successfully obtained with narrow CLD (CV < 0.6). These distributions appeared notably narrower than the literature (Supplementary Figure 7a). In comparison, direct distillation only separates the desired fractions from a existing mixture for downstream processes, with the undesired fraction wasted, leading to a lower yield than fractionated degradation.

b. Reduced operational costs and energy requirements. The degradation-distillation approach entails additional equipment costs, maintenance, and energy (~40% higher CO_2 emissions, Figure 4, Supplementary Table 16). In contrast, the fractionated degradation system represents a process intensification strategy that combines degradation and fractionation within a integrated equipment, reducing manufacturing expenses, facility floor area, and energy demands.[22]

β-Scission, side reactions, and impact of contamination

1. β-scission.

Previous density functional tight-binding (DFTB) simulations suggested that PP degradation occurs mainly through random β-scission (Supplementary Figure 26a), with disproportionation as a secondary pathway.[1] Our current observations consistent with this mechanism. Under near-ambient pressure in a Schlenk flask, the evolving molar mass of fragments aligns well with the random scission model (Supplementary Figure 15). Moreover, mathematics correlations between alkenyl density (mmol/g) and scission frequency ($s = 1/M_n$), suggesting α-alkene formation may directly relate to chain scission behavior (Supplementary Figure 11). Taken together, these findings indicate β-scission plays a significant role in PP degradation, which aligns with observations in other reactor systems.[23,24]

2. Analysis of side reaction and impact of contamination on fractionated degradation.

NOTE: *analysis of impurity effects on fractionated degradation products utilized exclusively real-life PP waste sourced from the plastic circular industry park in Jieshou, Anhui, China (W1, W2, W3). This selection was based on the site's high productivity (~ 8 wt.% of the world). While certain chemistry in the analysis may applicable to real-life plastic waste from other sites, our analysis must not be construed as universally valid. The findings represent rigorously scientific observations strictly confined to PP waste processed in the Jieshou circular industry park, including materials that underwent standardized collection, sorting, cleaning, and reuse protocols specific to this industrial park.*

The investigation of real-life PP wastes with varying purity focused on hydrocarbons collected from the T2 tray. This was based on prior performance data that PP-T2-5h-S demonstrated superior performance than those obtained from the T1 tray. Samples were designated as PP-T2W1, PP-T2W2, and PP-T2W3.

a. Aromatization and internal alkenyl formation. Internal alkenyl formation emerges as a notable side reaction during polyolefin degradation, with internal alkenes potentially serving as precursors to aromatics. Under appropriate conditions, alkenes may undergo cyclization and dehydrogenation leading to formation of aromatics. Non-catalytic thermal degradation of PP in inert atmosphere is initiated through radical reactions, including C-H and C-C homolysis, β-scission, or radical initiators (Supplementary Figure 26b). Subsequent dehydrogenation or disproportionation then generates alkenyl intermediates (Supplementary Figure 26c).[25-27] These structures capable of rearranging, cyclizing, and dehydrogenating to form cyclic and aromatic structures (Supplementary Figure 26d). For instance, intramolecular cyclization pathways such as Diels-Alder reactions could yield six-membered rings that subsequently aromatize to phenyl groups (Supplementary Figure 26d path II).

The HMBC-NMR analysis of PP-T1-5h and -T2-5h detected minor signals of internal alkenes and aromatics during virgin PP degradation (Supplementary Figure 10). In real-life PP waste, impurities enhanced internal alkenyl and aryl formation, probably through both ionic and radical pathways. We observed substantial increases in internal alkenyl signal with higher contamination levels (110-130 ppm), while α-alkenyl signals diminished (Supplementary Figure 22). This inverse correlation suggested that some internal alkenyls might derive from α-alkenyl, potentially through α-olefin polymerization, forming internal radicals, then underwent disproportionation or dehydrogenation (Supplementary Figure 26c, path III). Additionally, allylic methylene groups in α-alkenyls have higher reactivity than typical methylenes, which could facilitate hydrogen abstraction, leading to conjugated alkene formation near chain termini.

The formation of internal alkenyl groups corresponded to the reactions of CH_3, CH_2, and CH groups (Supplementary Figure 22). Specifically, the CH_3 signal near 20 ppm decreased sharply, likely due to internal alkenyl generation, causing CH_3 signal split with new peaks emerging near 22-23 ppm and at 14 ppm (allylic methyl in conjugated systems). These internal alkenyls appear highly reactive, further converting into aryl structures. This transformation correlated with peak intensification at 125-131 ppm (aryls) and 32-37 ppm (benzylic methylenes), particularly pronounced at the lowest purity levels (Supplementary Figure 22f).

b. Polymerization. Polymerization competes with polyolefin degradation according to the fundamental principle of polymerization.[28] The polymerization retards at high monomer conversions due to viscosity limiting diffusion to active sites. Under degradation (reduces viscosity and increases monomer concentration), polymerization rate would rebound, causing competition with degradation. Initiation of olefins could be induced by C-H homolysis or initiator (*e.g.*, oxygen, peroxides, or some impurities). The resulting radicals propagate and trigger intramolecular reactions with internal alkenyl groups (Supplementary Figure 26e), leading to crosslinked networks and cyclic structures. The polymerization and intramolecular reaction can be accelerated at elevated temperatures in the presence of radical-initiating species.

During PP fractionated degradation, polymerization of α-olefins might occur in the degradation unit and T2 tray. The product became more viscous with long reaction period (Supplementary Figure 22c). Kinetic analysis further confirmed this dynamic competition between polymerization and chain scission. The rapid chain scission dominated the initial 4 hours before plateauing due to polymerization (Supplementary Figure 22a).

c. Gasification. Gasification reaction producing light hydrocarbons (CH_4, C_2H_4, C_3H_6, etc.) typically intensifies at elevated temperatures. Gasification primarily proceed through chain-end scission, or dominant after complete chain scission (Supplementary Figure 26f). Gasification requires temperatures >500 °C for preferable conversion.[29] In our fractionated degradation systems, however, two factors suppressed

gasification: rapid removal of reactive fragments from the hot regions and the lower operating temperature (400 °C), as evidenced by the detection of only trace C_4 - C_7 alkenes in PP-T0-5h.

d. Branching. During fractionated degradation, chain scission generates free radicals that initiate branching through radical coupling, chain transfer, or other pathways (Supplementary Figure 26). The ^{13}C NMR analysis of PP-T2-5h reveals molecular tacticity variations and three characteristic signal groups at ~20 ppm (CH_3), ~29 ppm (CH_2), and ~45 ppm (CH). Significantly, signals in the 27-31 ppm region correspond to methine (CH) carbons, serving as potential branching indicators.[30]

For real-life PP waste, impurities in real-life plastic waste complicated the quantification. With high impurity level, the CH intensity reduced significantly due to alkenyl formation, converting saturated tertiary and quaternary carbons to unsaturated species, obscured branching analysis through signal overlap and signal shifts.

Nonetheless, branching represents a potential reaction during fractionated degradation, coexisting with radical coupling and polymerization, processes inherent to olefin radical chemistry. When alkyl radicals couple with other radicals or alkenes, they form tertiary or quaternary branch points (Supplementary Figure 26g). Furthermore, olefin polymerization (Supplementary Figure 26g) also generates branched structures.

3.Side reactions during the sulfonation

a. Olefin polymerization. In addition to radical-induced polymerization, olefins could undergo cationic polymerization when exposed to acid or cationic species (Supplementary Figure 26e, Path II). These reaction increase oil viscosity, induce color darkening, and ultimately degrade surfactant performance and functionality. Notably, such polymerization becomes particularly dominant under low viscosity, and tend to be dominant when the concentration of acidic initiator is relatively low, inhibiting radical coupling termination and offering reaction opportunity for activated center and monomers.

b. Over-sulfonation. We define over-sulfonation as a series of side reactions involving pyrosulfonations, coking, and anhydrides formation (Supplementary Figure 26h, Path I).[2] Insufficient heat transfer is the primary cause of over-sulfonation in industrial processes. The highly exothermic nature of sulfonation creates localized hot spots, which accelerate side reactions. In our experiments, over-sulfonation may occur through three pathways. First, when using olefins with a broad distribution, the rapid sulfonation of light olefins facilitates over-sulfonation with sulfonation reagents, resulting in a sulfonated mixture with dark coloration (Supplementary Figure 3b).

Second, when sulfonation conditions are poorly controlled, such as with slow stirring, overheating, or rapid feeding of the sulfonation reagent, localized hot spots of either temperature or SO_3 are created, causing local over-reaction.

Third, in the case of real-life plastic upcycling, the presence of impurities, particularly cationic species and metallic components, may affect the reaction. Some metals can absorb SO_3 onto its surface, converting SO_3 to SO_2 and sulfides.[31] Metal oxides can also incorporate SO_3 into the frameworks,[32] while some metal ion coordinate with SO_3, generating stable metal complexes.[33] Collectively, these interactions alter the electron structure on SO_3, either stabilizing or destabilizing SO_3 and redirecting the sulfonation reaction pathways.

For instance, introducing SO_3 at stoichiometric ratios in the presence of metal and metal oxide particles would lead to SO_3 absorption and reduction, thereby decreasing the yield of products. The generation of SO_2, sulfides, and other sulfur-containing byproducts would result in malodorous products and potentially cause secondary water pollution. If SO_3 becomes incorporated into an metals oxide framework and coordinates with metals, the reactivity and yields of the sulfonation reaction could be compromised.

c. Sultone. In alkene sulfonation chemistry, as the intermediate of sulfonation, sultones represent a special case.[2,34] While typically, sultones undergo hydrolysis to form hydroxyl sulfonates without compromising surfactant performance, β-sultones present unique challenges. After hydrolysis, the adjacent hydroxyl and sulfonate induces intramolecular hydrogen bonding, creating stabilized conformations possess poor surfactant activity (Supplementary Figure 26h, Path II).

d. Internal alkene sulfonation. It is noteworthy that a portion of internal alkenes have formed through the aforementioned pathways or been induced by SO_3 (Supplementary Figure 26h, Path III). The sulfonation of internal alkenes results in internal sulfonates. This side reaction is captured in our HT-NMR-HMBC characterization (Figure 3a), showing a minor shifts near δ 5.2 (^{1}H NMR).

Discussion of non-ideality

The proposed fractionated degradation demonstrated favorable size selectivity at both 10-gram and 10-kg scales. However, due to the complex and often unpredictable composition of real-life wastes, non-idealities are anticipated in pilot-scale experiments and industrial production. Consequently, potential non-idealities are outlined and discussed below. Future research targeting system optimization, modification, and pilot testing should consider these non-idealities to achieve optimal performance.

1. Thermodynamic non-Idealities.

a. Azeotrope formation. Azeotrope forms when intermolecular forces (*e.g.*, hydrogen bonding) create thermodynamic non-idealities in vapor-liquid equilibrium. These molecular interactions vary between compounds, resulting in deviations from Raoult's law that produce either minimum- or maximum-boiling azeotropes. Azeotrope systems establish barriers where vapor and liquid phases share identical compositions, preventing further separation through conventional distillation. Mixtures with divergent properties, such as benzene/toluene, benzene/cyclohexane, and water/methanol, are susceptible to form azeotrope.

Fractionated degradation of PP primarily yields α-olefins with similar molecular architectures. Ideally, mixtures composed exclusively of linear molecules would exhibit uniform intermolecular interactions consistent with Raoult's law, avoiding azeotropic phenomena and enabling relatively ideal component separation with sufficient theoretical plate. However, the ideality could be compromised by side products and impurities (Supplementary Figure 20), where the introduction of cyclic structures, oxygen contamination, or other impurities, may promote azeotrope or near-azeotrope and reduces separation efficiency. Typically, achieving complete separation of all components in near-azeotropic systems ($\alpha \sim 1$) requires operational adjustments such as increasing stage number, adjusting reflux ratios, installing de-entrainment devices, or implementing other compensatory measures. For true azeotropes ($\alpha = 1$), alternative approaches including impurity pre-separation, pressure-swing distillation, or extractive distillation may become necessary. Critically, the effect of azeotrope on fractionated degradation is currently unquantifiable due to thermodynamic complexity of real-life plastic waste and their degradation products (see discussion "*Characteristic of real-life plastic waste*"). Missing VLE data of fractionated degradation product from varied waste sources is a critical gap. The knowledge gap necessitates focus on chemical properties prediction or measurement, and will be discussed in the section of VLE deviation.

b. Foaming. Foaming occurs when trace surfactants or other polar impurities disrupt surface tension and viscosity. vapor is trapped in liquid, leading to entrainment and potential flooding. While foaming is common in practices like crude oil distillation and surfactants drying, foaming remains unlikely in operation of pure hydrocarbon mixtures. Specifically, for the fractionated degradation of virgin PP and real-life wastes (W-heze, W1, W2, and W3), no foaming was observed due to low polymer melt viscosity at 400℃ and minor polar contamination after proper cleaning procedure (Supplementary Figure 2a). However,

polyolefins are frequently used in detergent containers. Improperly cleaned feedstock or accidental supply-chain contamination may introduce active species. Mitigation strategies include thorough cleaning, adding antifoam reagents, and operating degradation with controlled heating rates to moderate vapor flow.

c. VLE deviation. VLE deviations occur when experimental or predicted phase behavior diverges from actual performance, primarily due to incorrect assumptions or VLE data. Modeling approximations (*e.g.*, Wilson, NRTL, and UNIQUAC equations) require accurate parameters that often unavailable or inaccurate for innovative systems. While recent Aspen Plus V14 updates incorporated polymer pyrolysis databases, viscosity, thermodynamic, degradation kinetics, multi-component interaction parameters of polymers remain absent. Consequently, determining VLE parameters remains a significant challenge for industrial process design and simulation.

Future research aiming to optimize or modify the fractionated degradation system for industrial applications should prioritize minimizing VLE deviations. Regression-based prediction methods offer a effective pathway for obtaining reliable VLE data. By precisely determining key liquid and vapor molar ratio across temperature and pressure ranges, the physical properties can be fitted, either manually or through automated regression tools in ASPEN. In our simulations, the miss BIP parameters were regressed using data in Supplementary Figures 9 and 10. In addition, missing VLE parameters can also be estimated through group contribution methods.[35,36] The group contribution methods enable parameter determination via manual or software computation without experiment. However, group contribution methods typically deliver lower accuracy than experiments or regression fitting, though it remains valuable for system assessments when the other techniques are unavailable. Ultimately, for practical implementation, regression-derived and simulation-based parameters should not be accepted without validation against actual pilot-scale operating records where available.

2. Equipment & Hydrodynamic Non-Ideality.

a. Entrainment. Entrainment occurs when vapor velocity lifts liquid droplets or foam from a lower to the upper tray. This phenomenon arises when vapor energy overcomes gravitational forces and liquid film surface tension, primarily driven by high vapor flow rates and pressure gradient. The upward flow contaminates streams in the upper trays. Consequences include broadened distribution, off-specification products, reduced separation performance, higher heat duty, and even column flooding. In the fractionated degradation of polymer, entrainment poses the critical threat to product uniformity. While largely mitigated in our conceptual design after equilibrium (Supplementary Figure 5), the entrainment during system initiation caused distribution broadening (PP-T1-1h and PP-T2-1h; Supplementary Figures 8-9).

To suppress entrainment in fractionated degradation systems, the following design and operational modifications could be helpful:

(1). increase tray spacing and expand reactor diameter to reduce vapor velocity;

(2). install de-entrainment plate above output trays or within vapor transfer direction;[37]

(3). choose proper tray types that compatible with expected vapor-liquid loading ranges;

(4). for foaming systems, one may extend liquid flow paths and incorporate anti-foam devices;

(5). reduce reboiler duty and control heating rate to moderate vapor generation rates;

(6). introduce anti-foam reagents to suppress bubble formation (need compatibility with process to prevent contamination).

b. High pressure drop. Pressure arises from frictional resistance during vapor flowing through trays. Factors causing pressure buildup include equipment flaws (improper internals installation, damaged trays) and operation conditions. Maldistribution of flow exacerbates pressure value and creates localized high-pressure zones. Potential consequences of high pressure include flooding and increased operation costs in vacuum systems.

Our experimental data showed controlled pressure drop of <1 kPa (Supplementary Figure 5e), within normal operational range. However, in industrial implementation, risks of entrainment and flooding caused by high pressure during startup should be considered. Wet feedstock and rapid degradation (caused by catalyst or uncontrolled heating) accelerate pressure buildup at the reactor bottom. To mitigate high pressure, tray engineering is critical for effective management of hydraulic behavior in the reactors. Sieve trays with large open area and small pore, valve trays, and cross flow trays are potential options.

Future work may prioritize scale-up studies and pilot testing to examine tray configurations for industrial fractionated degradation (experiments at laboratory scale is challenging due to difficult tray manufacturing at small scale, diameter ~ 2-3 cm). Anti-fouling properties of tray coating should also be included in relevant study, particularly, for real-life plastic wastes.

c. Back mixing. Back mixing refers to the unintended reversal of liquid or vapor flow, such as entrainment, disrupting the counter-current that essential for efficient separation. Back mixing of liquid occurs on trays due to liquid recirculation, excessive hydraulic gradients, or weeping. Back mixing of vapor arises when vapor jets penetrate liquid layers or when significant vapor bypasses around or through damaged trays. The back mixing disrupts the concentration gradient along the mass transfer direction, causing fluid mixing between adjacent trays and compromising Murphree tray efficiency. The consequences of back mixing include diminished product purity, increased energy consumption, reduced throughput capacity, compromised process controllability, etc.

Back mixing is critical for industrial applications and requires thorough tray engineering investigation.[37] Key layout parameters, flow path length, outlet weir height, flow path width, etc., require optimization to minimize back mixing. Crossflow trays and high-performance sieve trays can reduce back mixing, as well as pressure drop, fouling resistance, and entrainment.

3. Kinetic non-ideality.

Intrinsic kinetic. Kinetic parameters from polymer degradation studies often reflect apparent rather than intrinsic kinetic values due to coupled factors, including side reactions, heat transfer limitations, mass transfer effects, etc.[38] For example, thermal gradients in the reactor slow down the degradation reaction and could cause deviations from the intrinsic kinetics. Even with minimal heat and mass transfer limitations, kinetic curves may show deviations, attributable to the side reactions (Supplementary Figure 22a). In addition, during the fractionated degradation, characterizing molar mass evolution (polymer to small molecules) using a single analytical approach is challenging, while using integrated techniques may introduce systematic errors.

Acquiring intrinsic kinetic parameters presents critical experimental challenges. To approach the intrinsic value of polymer degradation, the kinetic experiments employed judiciously designed setup and processes. A small thin-walled Schlenk flask was heated with thick asbestos insulation under controlled conditions (~400°C, ~1 atm). Molar mass characterization employed APCI-MS and GC-MS, with kinetic analysis focused specifically on the linear region (0.5-4 hours). Through mitigation of mass/heat transfer limitations, simulation of degradation unit conditions, and rigorous post-model fitting, the derived kinetic parameters can optimally approach the intrinsic values of PP random scission. To better determine intrinsic chain scission kinetics, judiciously designed microreactors featuring sub-mm channels might achieve near-isothermal conditions and further reduce diffusion timescales. Additionally, material forms (thin films, fibers, or nanoparticles) can be optimized to accelerate heat transfer. Computational deconvolution methods may provide valuable insights as well, for example, iterative solutions of energy/mass balances coupled with reaction equations using computational fluid dynamics.

Distribution assessment

The distribution of hydrocarbon products requires a proper descriptor for scientific comparison. With the inertia of polymer chemistry, PDI is inherited to described product distribution in some earlier research on plastic recycling.[1,39,40] Although, PDI is widely used to characterize polymers and oligomers,[28] it is a ratio of average molecular weights with the distribution being normalized. Therefore, mathematically, PDI does not directly describe the distribution. Since PDI is a ratio, for a given value, the distribution range tends to increase as the average molecular weight rises (Supplementary Figure 7b). For example, the PDI of a mixture with 25 mol% C4, 50 wt.% C5, and 25 wt.% C6 is 1.02. However, it would be misleading to suggest that the distribution of this C4-C6 mixture is equivalent to that of a standard polymer reference, as the latter can span a broad range from a few kDa to several hundred kDa. Therefore, using PDI as a distribution descriptor is inappropriate in this context, as it is mathematically irrelevant and may provide inaccurate information about the distribution. To provide a more accurate description of hydrocarbon distributions, alternative statistical descriptors were explored (Supplementary Equations 1, Supplementary Figure 7a, and Supplementary Table 2).

S quantifies the degree of randomness or disorder within a dataset. It is calculated as a weighted sum of the probabilities of different outcomes, with the weights determined by the logarithms of those probabilities (Supplementary Equation 1). As such, S is highly sensitive to the shape and uniformity of the data, rather than the distribution. For example, the entropy of PP polymer is only slightly higher than that of the hydrocarbons in our study and in the literature (Supplementary Table 2). The entropy of a light hydrocarbon mixture,[41] although appearing narrower than the others, ultimately converges to a value that aligns with the rest of the distributions (Supplementary Figure 7a and Supplementary Table 2).

In contrast, the σ directly reflects the spread of a distribution. Mathematically, σ is defined as the square root of the variance, which measures the average squared deviation of each data point from the mean. This provides an indication of how widely the values are dispersed within the distribution. For instance, the σ of PP polymer is significantly greater than that of its degradation products (Supplementary Table 2). Additionally, the σ values for PP-T0-5h and β+Pt@Hie-TS-1 (gases and short hydrocarbons) are smaller than other liquid and solid hydrocarbons. Similar to σ, the CV is a relative measure of dispersion, comparing the σ^2 to the mean. This provides insight into the variability relative to the size of the mean. Since the σ is influenced by the range of the data, CV offers an indirect measure of the range. However, unlike σ, CV is particularly advantageous when dealing with datasets that have high skewness in scale between groups. In such cases, CV provides a more reliable measure of variability because it normalizes the σ and σ^2 by the mean, ensuring a consistent measure of dispersion regardless of the absolute scale of the data.

Characteristic of real-life plastic waste

1. Definitions.

To discuss real-life plastic waste, it is essential to first define "virgin plastic", "after-use plastic", and real-life plastics. According to the United Nations Development Programme (UNDP), virgin plastic refers to plastics that are newly manufactured from petrochemical feedstocks, and have never been used before.[42] After-use plastic or post-consumer plastic waste is derived from the UNDP's definition of single-use plastic, with slight modification, and refers to plastic items that are discarded or recycled after being used a limited times.[42] Real-life plastic waste, as adapted from the UNDP Plastic Pollution website, is broadly defined as plastic found in landfills, natural environments, or recycling facilities, whether sorted or unsorted, clean or contaminated.[43]

2. Complicity of real-life plastics wastes.

Real-life plastic waste exhibits greater complexity than virgin or regenerated plastics. Additive and

contaminant often vary significantly between batches (Supplementary Figure 22d). This variability introduces uncertainty in determining precise chemical structures and concentrations within waste streams, making conventional analytical frameworks time-consuming and of limited practical value.

To characterize the composition of these waste materials, we employed standardized analytical methods based on visual examination (Supplementary Figure 22).[7,8] The visual methodology is widely adopted due to its efficiency and reliability in characterizing heterogeneous materials. In this work, we categorized impurities into two categories: mixed impurities (macroscopically heterogeneous) and blended impurities (microscopically heterogeneous).

3. Composition analysis of real-life PP wastes.

Mixed impurities. Mixed impurities are foreign materials occupying distinct domains within plastic mixtures (Supplementary Figure 20). These macroscopic and phase-separated impurities source from contamination during transportation and are typically immiscible under ambient conditions. Compositional analysis of W3 (Supplementary Figure 22d) identified natural polymers and paper (biomass) as a prevalent polymeric impurities at 1.2 wt.%. Visual inspection further detected trace quantities of animal-derived materials (<0.1 wt.%, categorized as "Others") and rubber components (1.0 wt.%). Other plastics including PET and PVC were also identified through visual inspection at minimal levels (<0.1 wt.%, categorized as "Others"). Although PE shares spectral (visual light and infrared) similarities with PP, their degradation pathways differ fundamentally. PP degradation yields predominantly alkenes, while PE degradation produces ~40 mol% alkanes.[1] Our quantification of PP-STW-6h revealed alkane formation (~3.5 mol%, Supplementary Figure 23b), indicating PE contamination. Using APCI spectral analysis and assuming 40% alkane yield from PE degradation, we estimated the PE fraction at ~10 wt.% (Supplementary Figure 19c), establishing it as the dominant polymer impurity in W-heze.

Other than polymeric components, metals emerged as significant mixed impurities. These metals may not be introduced during manufacturing, and are likely introduced through supply-chain contamination. In the W3 sample, metal content reached ~5.8 wt.%, potentially introduced through supply-chain contamination. Characterizing the precise mineral composition presents challenges due to heterogeneous distributions and particulate variability at sub-sampling scales.

Blend impurities: Blended impurities, conversely, consist of additives integrated or dissolved within materials during manufacturing, including stabilizers, pigments, antioxidants, and plasticizers.[44,45]

To identify potential additives within the mixture, we employed a deductive approach correlating elemental composition with specific additive sources. For instance, post-consumer polyolefin waste from packaging and household goods contains metallic components derived from stabilizers, pigments, and fillers, including $BaSO_4$, TiO_2, AO-series, S-series, P-series, and EPDM,[44] corresponding to elements (Ca, P, Ba, Co, S, etc.) detected in our elemental analysis (Supplementary Table 12 and Supplementary Figure 22). Following, potential impurities are discussed by element, with example compounds listed in parenthesis.[44,45]

Ca primarily originates from stabilizer additives like calcium stearate, flame retardant (calcium magnesium hydroxide oxide), and pigment ($CaCO_3$ and calcium resinate).

Mg typically derives from mineral fillers such as talc, stabilizer (magnesium carbonate), and pigment (magnesium ferrite, magnesium sulfate).

P primarily originates from phosphite stabilizers (P-1, P-2, and P-3, triisotridecyl phosphite) and flame retardant ((2-cyclohexylphenyl) phosphite, Tris (3-chloropropyl) phosphate).

S is introduced through thiol-based antioxidants like S-1 (DSTDP), S-2, and S-3 (DLTDP). Other sources include stabilizer (methanesulfonate, pentalead tetraoxide sulfate), pigment (zinc sulfide), and antistatic agent.

Na, Al, and Si may come with each other.

Na is sourced form pigment, such as sodium docusate and ultramarine blue ($Na_8Al_6Si_6O_{24}$).

For Al, in addition to pigment, it may also come from flame retardent (sodium aluminate, sodium aluminosilicate, sodium aluminum phosphate), stabilizer ($Al_2Ca_6O_6(SO_4)_3$, aluminum hydroxide), and pigment (Al, Al_2O_3).

Si sources are vary. Other than pigment, many silicates (Pb, Mg, Zn, Zr) are plastic stabilizers.

Due to the dominance of Na, Al, and Si in lithosphere and ocean, these element can also be introduced through contamination during transportation.

Br is predominantly associated with flame retardants such as brominated phosphates, hexabromocyclododecanes, and tetrabromobisphenol A.

Ba is primarily from barium stabilizers (stearate, acetate), colorants (1-naphthalenesulfonate), monomer (barium nonylphenolate), and fillers (element or oxidate) in plastics.

Sr is commonly attributed to Sr-Zn stabilizers, strontium chromate colorants, and $SrTiO_3$-based electronic fillers in e-waste.

Pb is frequently introduced as stabilizers, as element lead, chloride, naphthenate, phthalate, phosphite, or oxides. Other application includes colorant (lead chromate), crosslinker, and hardener (tetraethyl lead).

Zn often comes with other metals. For example, Zn is a common element in lubricants (Al-Mg-Zn carbonate hydroxide) and pigments (Zn chromate, Zn oxide).

Zr is well-known a key element of Ziegler-Natta catalyst, and may come with Al, Zn, Ti, and Fe.

Fe is sourced from many applications. As an additive, Fe is mostly utilized as colorants (*e.g.*, iron chromate, iron manganese trioxide, and iron oxide). Yet, it can also be introduced into real-life waste by rust during transportation and mechanical wear from pulverization equipment.

Cu, a non-ferrous metal, is often used as pigment or an ingredient of pigment.

Inorganic fillers sometime constitute the most predominant additives (30-40 wt.%), added to enhance mechanical properties of regenerated plastics. For example, the filler material was observed during the fractionated degradation of W-heze at 10 kg scale, producing ~12 wt% gray solid residue and ~87 wt.% of PP-STW-6h (Figure 23). PXRD analysis revealed diffraction patterns corresponding to $CaCO_3$ (**Supplementary Figure 23a**).

4. Dynamic and Unpredictable real-life plastic wastes.

The heterogeneous, spatial, and temporal nature indicate that the average property of real-life plastic waste is dynamic.[46,47] Its characteristics are influenced by both internal and external factors, making its composition and chemistry difficult to be forecasted. As a result, large-scale experiments offer a partial solution for investigating real-life plastic waste upcycling. At larger scales, macroscopic variations can be minimized, enabling more controlled studies. In this study, we conducted fractionated degradation of real-life plastic waste in a scale-up reactor. This allowed us to treat the composition of real-life PP waste as a simplified, averaged lump, assuming that the composition remained relatively constant. We compared the degradation reactions of pure PP and real-life PP to assess potential differences and explore the impact of impurities on the upcycling process.

Applicability for mixed and contaminated polyolefin wastes

Properly sorted, cleaned, and purified real-world PP wastes (W-heze, W1, W2; Figure 2d, Supplementary Figures 19 and 22) yielded dominantly α-olefins. The resulting PP-STW, PP-T2W1, and PP-T2W2 exhibit minimal contamination and trace internal and aryl structures (Supplementary Figure 22). The W-heze (87 wt.% PP, 10 wt.% PE, 0.7 wt.% rubber, 1.7 wt.% biomass, 1.4 wt.% other, Supplementary Figure 19c) exemplifies the process applicability to mixed polyolefin with contamination, yielded PP-STW-6h (87 wt%) with dominantly α-alkenes and minor alkanes (~3.5 mol%). Critically, the metallic impurities level of

PP-STW-6h was low (Supplementary Table 12), while metal could precipitate sulfonates, it showed negligible impact on the specific case of W-heze upcycling to sulfonate (Figures 4c, 4d).

In contrast, severely contaminated PP waste (*e.g.*, PP-T2W3: Al ~1.7 g/kg, Fe 1.7 g/kg, Ba 0.5 g/kg; Supplementary Table 12) or PP waste with high PE content, may compromise upcycling applicability. Impurities (metals and organic impurities) reduced α-olefin dominance during fractionated degradation, generating greater unsaturation through internal alkenes and aryl compounds (Supplementary Figure 22). High-PE feedstocks further exacerbate side reactions, producing substantial alkanes (~40 mol% for pure PE),[1] which reacts less efficiently than α-olefins. The alkane portion may be converted to internal alkenes via hydrogen abstraction (Supplementary Figure 26h, Pathway III), and then promotes polysulfonate formation, polymerization, coke, and internal sulfonates (Supplementary Figure 26e). Metals (*e.g.*, Ca, Mg, Fe) intensify coloration and precipitate sulfonate (*e.g.*, Ca-sulfonates).[48] Crucially, while hydrolysis treatments remove metals and some organics, they cannot prevent irreversible persulfonate and sulfonated byproducts that degrade detergency, disrupt hydrophilic-lipophilic balance (HLB), reduce solubility, darken color, and impair emulsification.

Hence, the proposed method demonstrates applicability for upcycling mixed polyolefin and contaminated wastes into surfactants, though real-world utility remains application-specific. When processing PP-rich wastes (>95 wt.%), or PE-rich streams using modified conditions (elevated degradation temperature, acidic catalyst),[1,49,50] the approach yields high-performance surfactants comparable to the commercial benchmarks. However, for severely contaminated wastes, product complexity may compromise viability of our method in high-value applications like household cleaning, enhanced oil recovery, precision emulsifiers, and corrosion inhibitors.[51] These materials, nonetheless, may serve effectively in industrial cleaning, heavy metal removal, and low-end detergents where blended sulfonates meet performance requirements.

Potential biodegradation pathway

The biodegradation pathways of PP-T1-5h-S and PP-T2-5h-S may align with alkyl sulfonate biodegradation pathway. Taking sodium dodecylbenzenesulfonate (SDBS) as an example, the most commercially significant sulfonate surfactant, the process typically initiates with alkyl chain oxidation.[52] This initial transformation involves ω-hydroxylation, where microbial enzymes including alkane 1-monooxygenase and cytochrome P450 systems convert terminal methyl groups into alcohols. These hydroxylated intermediates then undergo sequential oxidation: first to aldehydes, then to carboxylic acids. The resulting derivatives subsequently enter β-oxidation pathways, where they are cleaved into smaller molecular units that serve as carbon substrates for microbial metabolism (Supplementary Figure 25h). Concurrently, the aromatic component degradation may take two pathway. Aryl group could undergo direct enzymatic conversion. Bacterial oxygenases hydroxylate the benzene ring, forming catechol derivatives that undergo either ortho- or meta-cleavage. Aryl group could also be converted into benzoyl compounds. The β-oxidation consumes alkyl chain and eventually leave a carbonxyl group the aryl.[52] Both processes ultimately yields water-soluble aliphatic acids, thereby significantly reducing the environmental persistence and ecotoxicity characteristic of sulfonate compounds.

Outlook

1. Comparison with representative methods for distribution controlling. Current approaches to size-selective degradation predominantly dependent on catalytic and chemical engineering pathways. While catalytic hydrogenolysis represents a common methodology (Supplementary Table 17 and Supplementary Figure 7a), which necessitates sophisticated catalyst design and preparation involving costly noble metals

like ruthenium and platinum.[40,53-57] Fundamental limitations including high-pressure operational requirements, poor impurity tolerance, and expensive preparation protocols continue to restrict practical implementation of these systems.[1,58] The production of alkane-dominated outputs further constrains downstream utility for fine chemical synthesis. Alternative strategies employing engineered reactors enable direct degradation under ambient pressure without hydrogen or catalysts, yielding olefin-rich products from PP and PE that are suited for functional chemical manufacturing.[1,20] Nevertheless, both catalytic and non-catalytic methods generate hydrocarbons or oligomers with broad carbon distributions, or narrow-distributed light hydrocarbons. The divergent reactivity between short- and long-chain olefins compromises reaction uniformity in downstream processing.

The fractionated degradation demonstrates promise for advancing this field. By converting PP into narrowly distributed C_6-C_{15} and C_{15}-C_{30} α-olefins, this methodology enables precise valorization pathways such as sulfonate surfactant production. The ambient-pressure operation and catalyst-free operation position this approach as a potentially transformative solution for circular plastic economies. More importantly, this catalyst-free platform establishes a robust and scalable foundation for transforming plastic waste into functional hydrocarbons. Beyond surfactants, this technology opens high-value routes to specialty chemicals including petroleum derivatives, advanced lubricants, battery separator materials, and pesticides.

2. From laboratory to industry. The transition from prototype reactors to industrial-scale operations requires significant improvements in efficiency and profitability. First, implementing professional engineering designs, such as internal tube heaters and helical turbulators to improve heat transfer, and feedstock pre-heating systems to shorten heating period.

Second, adopting continuous operation represents a advancement in production efficiency. Unlike fractionated degradation in batch mode, which requires 4-5 h to achieve thermodynamic equilibrium, continuous systems capitalize on dynamic equilibrium conditions. Although not experimentally validated in the current study, the similarity with petroleum refining practices suggests considerable potential for continuous production. This approach would maximize reactor utilization and increasing output capacity.

It should be noted that while continuous processing aligns with regulatory preferences and offers inherent efficiency advantages, commercially successful batch and semi-batch systems remain viable for plastic degradation, exemplified by the Resynergi's microwave-assisted approach. Following the example, clustered reactors for fractionated degradation may demonstrate advantages in thermal efficiency and energy conservation. Our conservative techno-economic analysis (Figure 5) confirms the economic robustness of batch operations, indicating that batch and semi-batch modes are valid alternatives when continuous mode proved unnecessary, impossible, or challenging.

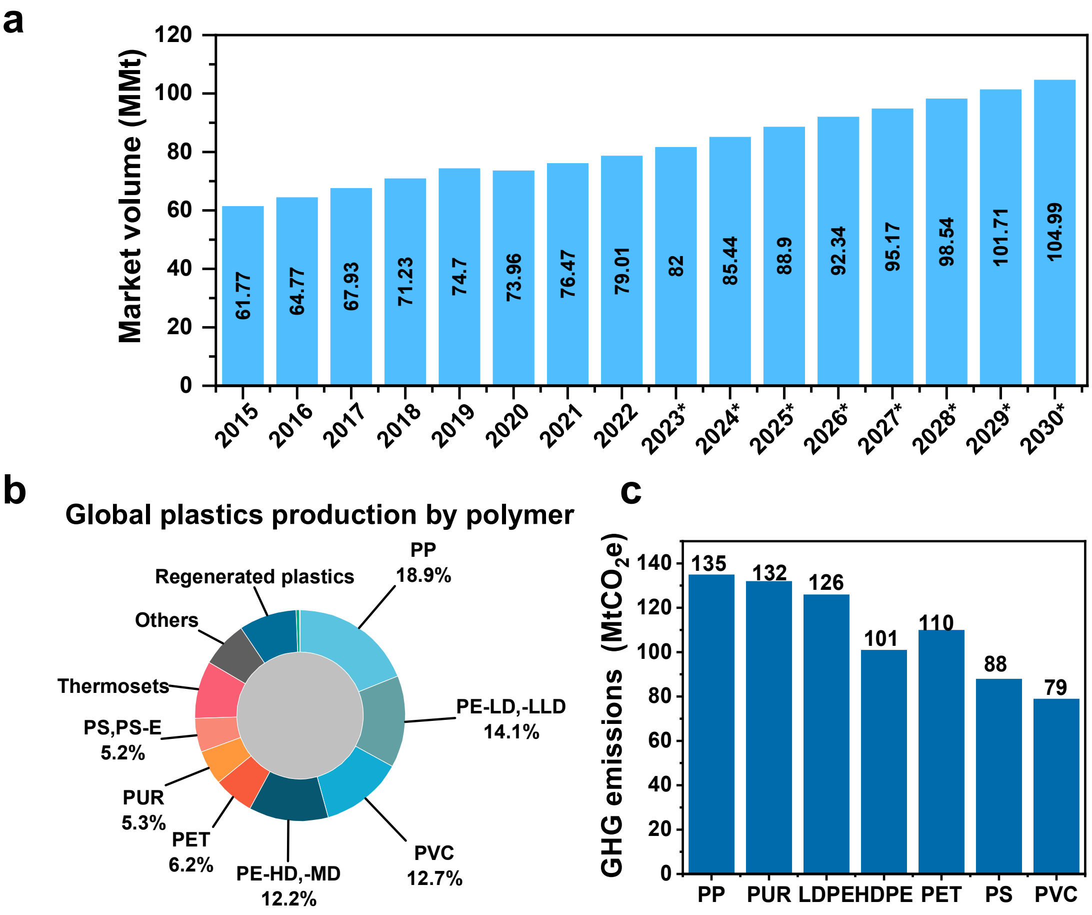

Supplementary Figure 1. (a) Market volume of polypropylene worldwide from 2015 to 2022, with a forecast from 2023 to 2030, as reported by the updated database of Organization for Economic Co-operation and Development (OCED).[59] (b) Global plastics production by type in 2023.[60,61] (c) Global GHG emissions of plastics by types in 2015.[61-63]

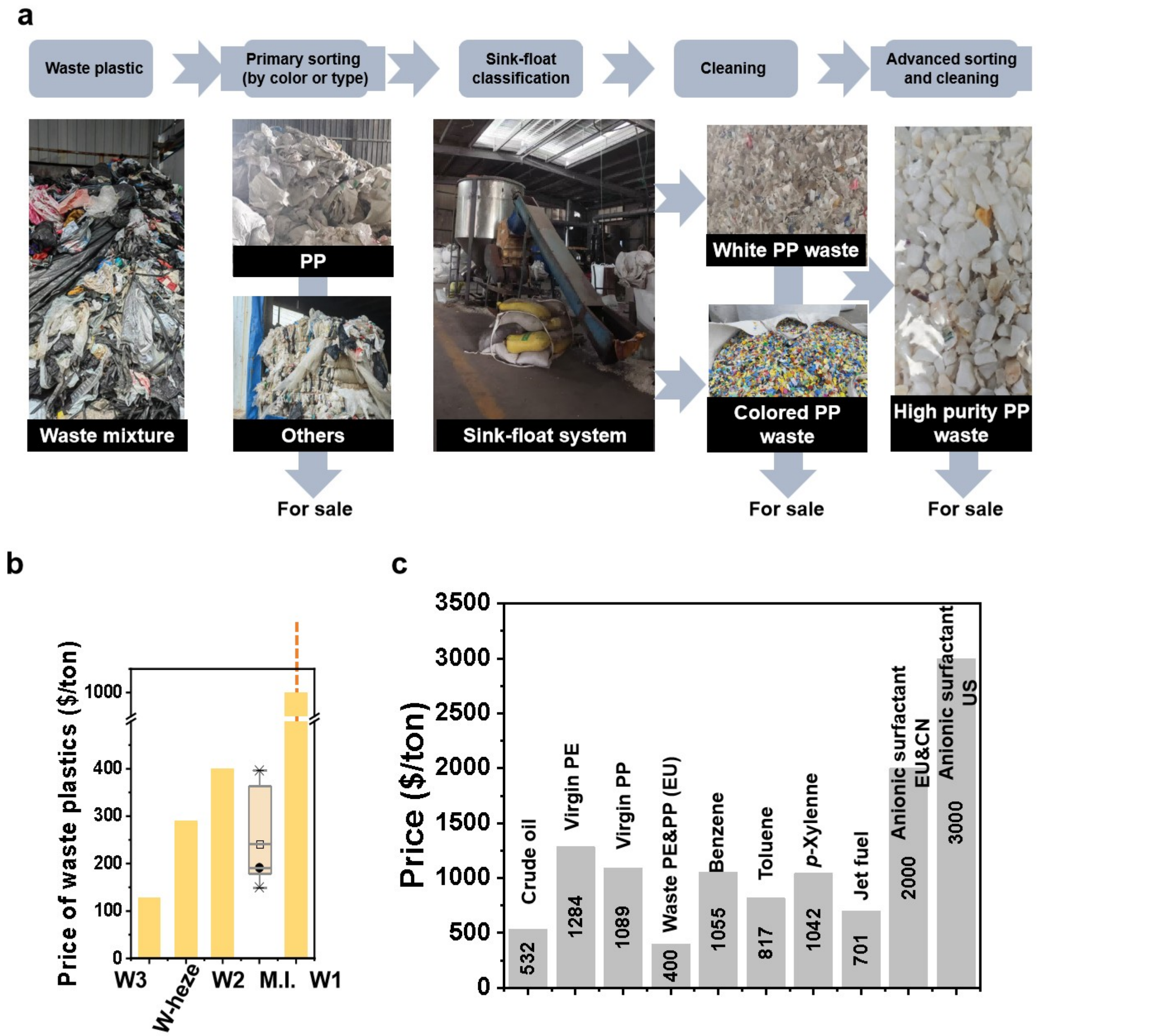

Supplementary Figure 2. Field research and market investigation. (a) field research of plastic circulation industry park in Jieshou (Aug. 2024). The flow chart represent a potential process flow of plastic wastes to obtain PP waste of different purity. (b) Quoted prices of real-life PP waste during the field research. Note that the prices are rough estimations and could fluctuate. W1, W2, W3 are purchased from the Jieshou plastic circulation industry park to investigate potential effect of impurity on products. W-heze was purchased from the local municipal recycling depot in Heze city. The degradation of W-heze in our scale-up reactor generated PP-STW. The box chart of M.I. represented prices of waste PP cited from https://jiage.zz91.com/suliao, (July 14, 2025). The box captures the maximum (top line), 75th percentile (2nd line), mean (3rd line and square), median (4th line and dot), 25th percentile (5th line), and minimum (bottom line). (c) Bruent oil price (Nov 2024) per ton was recalculated from barrel; Virgin PE and PP were obtained from IMARC reports;[64,65] Approximate prices of waste polyolefins were obtained from EU joint research center report;[66] Prices of benzene, toluene, and *p*-xylene were obtained from ECHEMI, Market price&Insight website (Nov. 2024); Price of jet fuel was obtained from IATA Jet Fuel Price Monitor website (Nov. 15, 2024); Approximate prices of anionic surfactants in EU, CN, and US were obtained from ChemAnalyst report.[67]

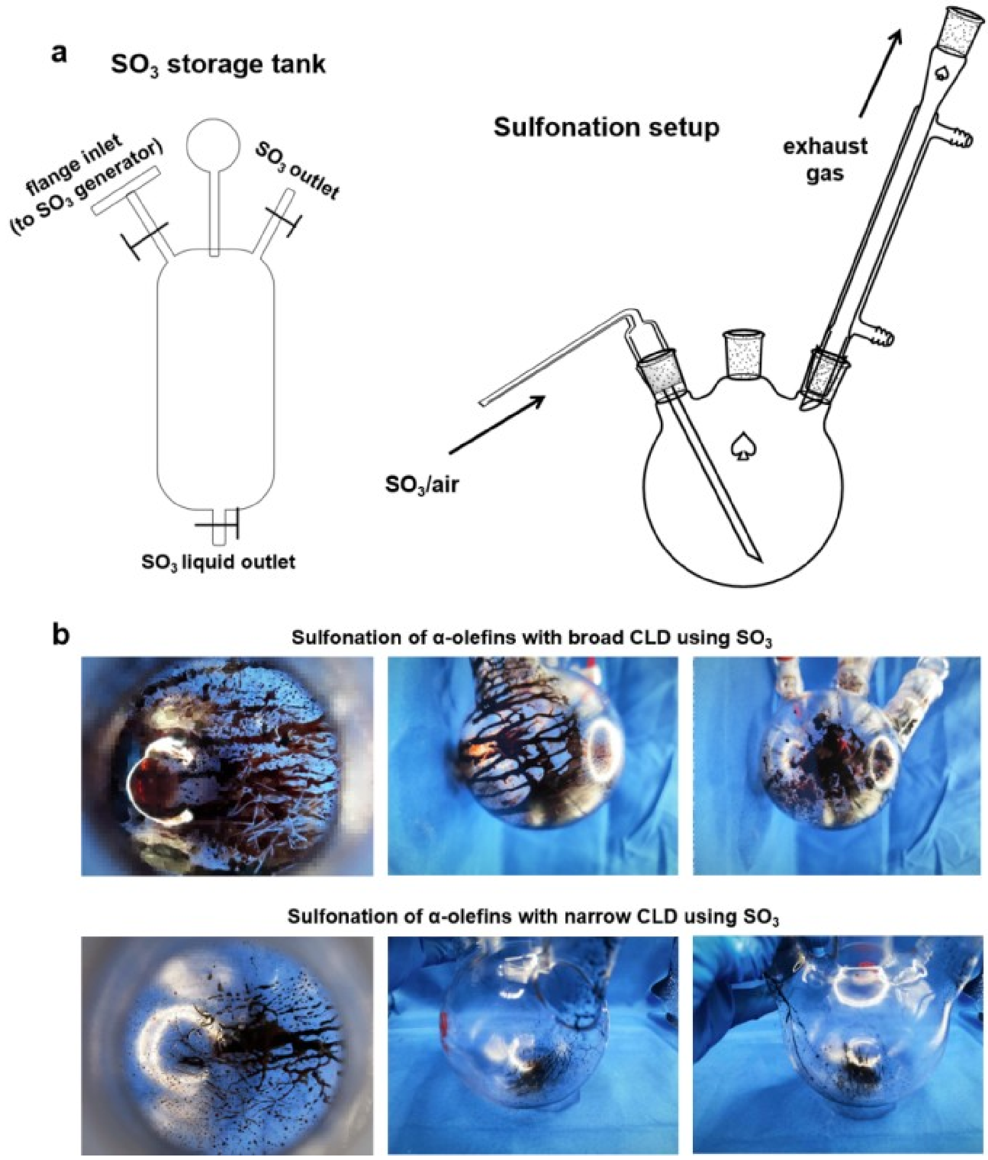

Supplementary Figure 3. Exploratory studies of SO_3-based sulfonation for α-olefins with broad and narrow CLD. (a) SO_3 storage tank and sulfonation setup, connected with a Teflon® tube and sealed with Teflon® tape; (b) Image of coke formed after sulfonation, leaving the black residue. The a-olefins with narrow CLD showed better controlability than that with broad CLD.

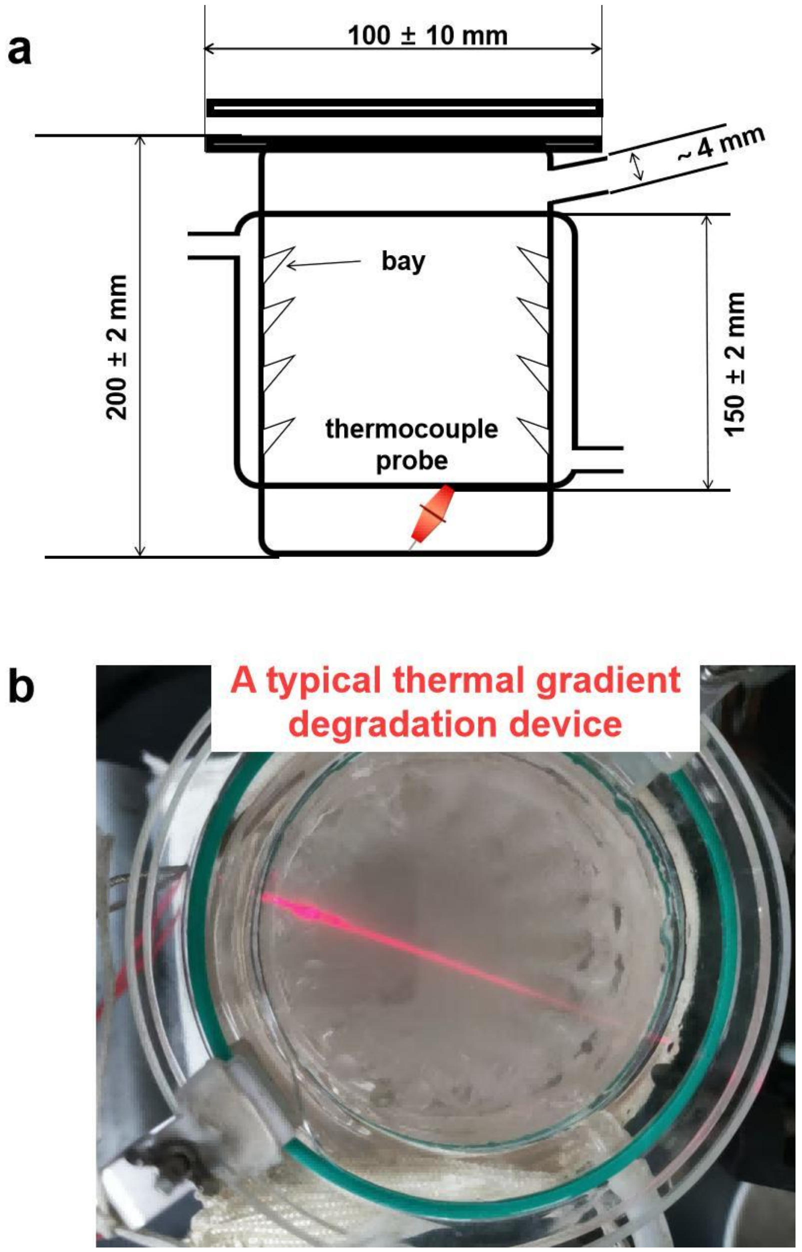

Supplementary Figure 4. (a) Technical drawing of a typical thermal-gradient non-fraction degradation reactor. (b) Digital image showing strong entrainment within the reactor. The red laser was directed near the product outlet of the thermal gradient reactor. The light intensity of the trail significantly decreases due to scattering caused by the high density of droplets from entrainment. This observation was corroborated by the broad distribution of PP-NT-5h (Figure 1 and Supplementary Figure 9) and previous research.[1,20]

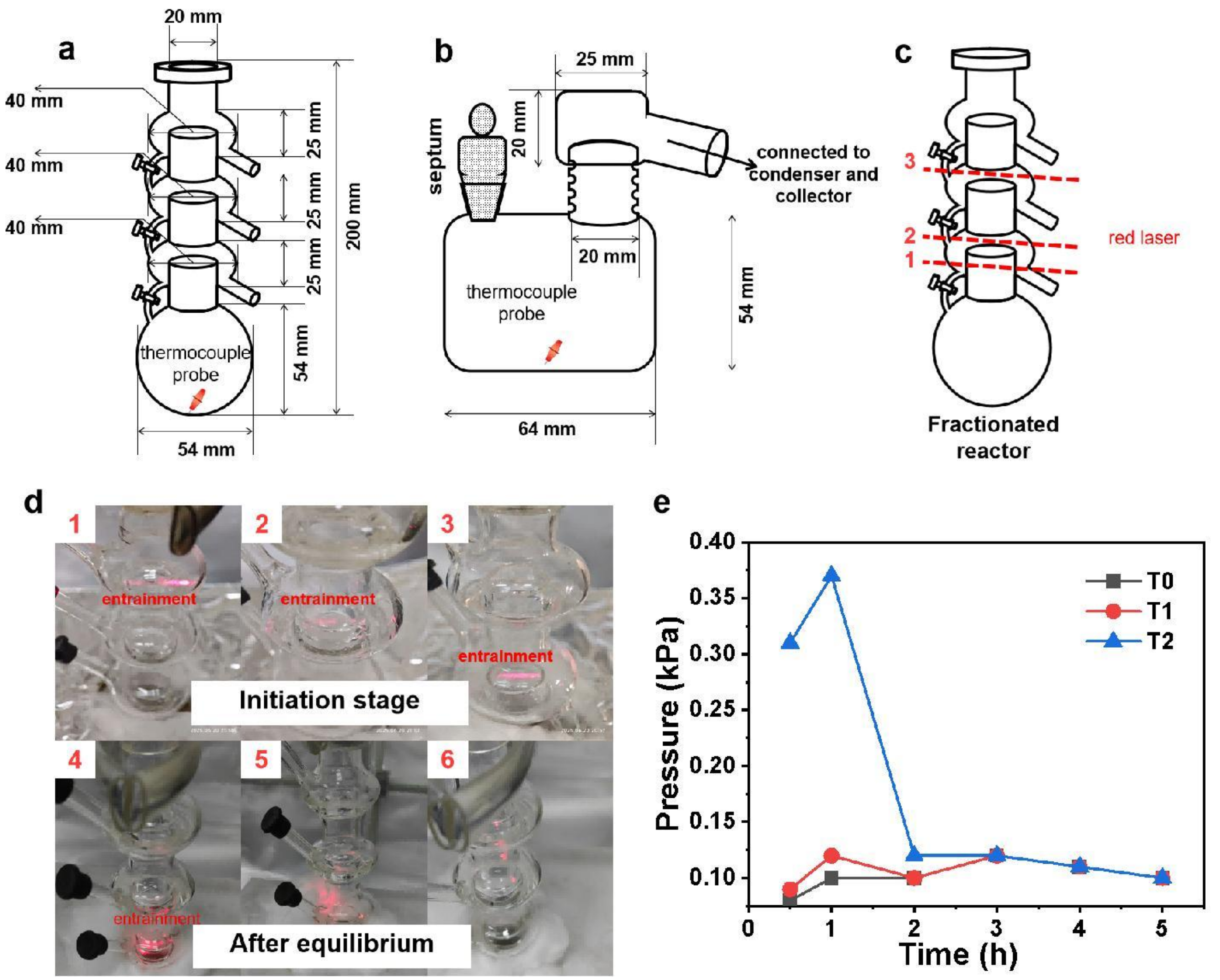

Supplementary Figure 5. Technical drawing of (a) a three-fraction reactor and (b) a single-fraction reactor. (c) Schematic showing laser positions used to examine entrainment: 1 - bottom of T2, 2 - middle of T2, and 3 - middle of T1. (d) Digital images of fractionated degradation in a three-fraction reactor at the initiation stage and after equilibrium. During initiation (0-0.5 h), pronounced entrainment phenomena were evident across all trays. After equilibrium, entrainment effects were largely mitigated, persisting only minimally at the lower section of T2. (e) Pressure monitoring in the reactor at three tray levels.

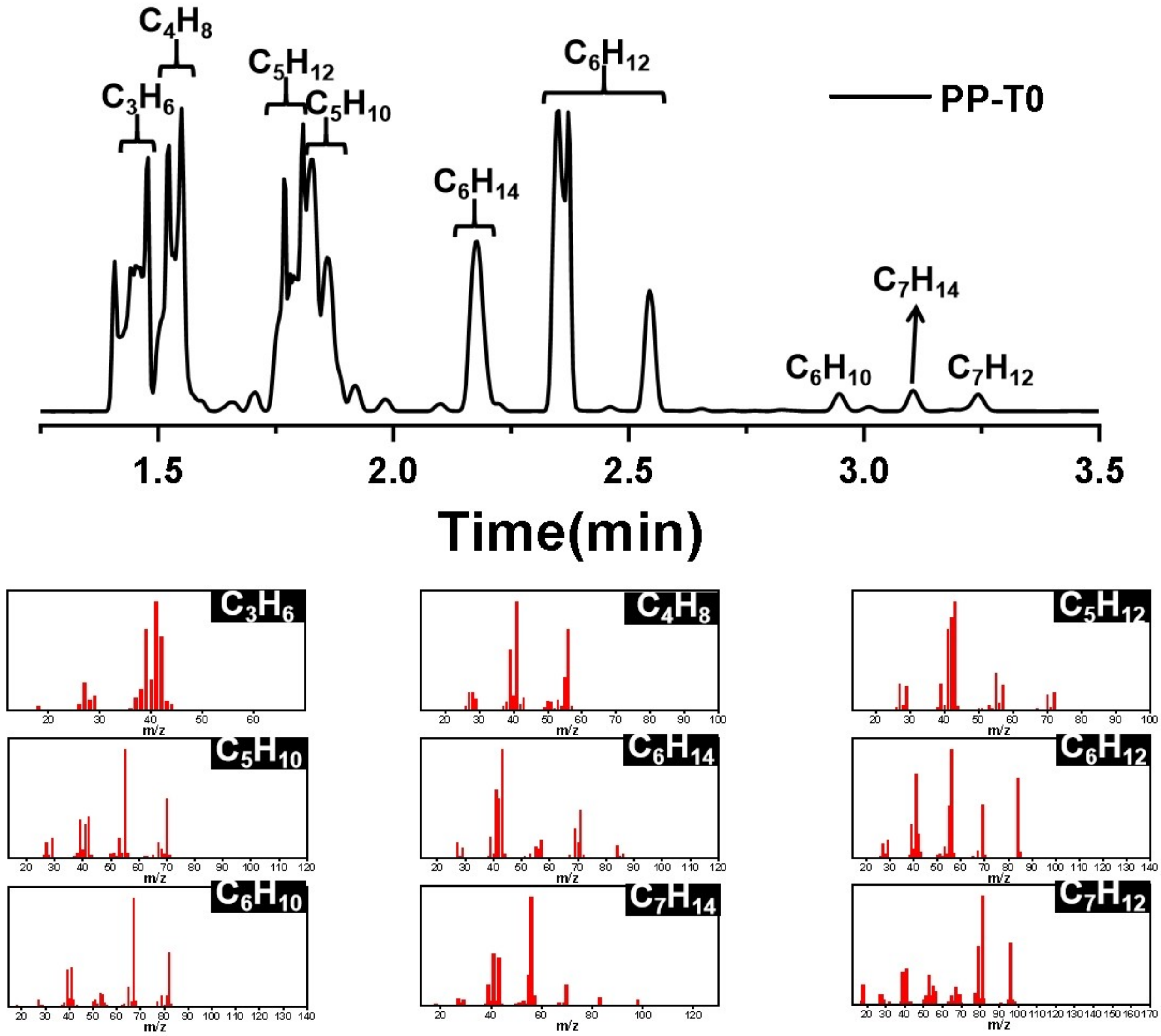

Supplementary Figure 6. GC-MS analysis of PP-T0-5h. The chromatogram displays various hydrocarbon species identified at different retention times. Detailed information on average molecular weights and distribution is provided in Supplementary Tables 1.

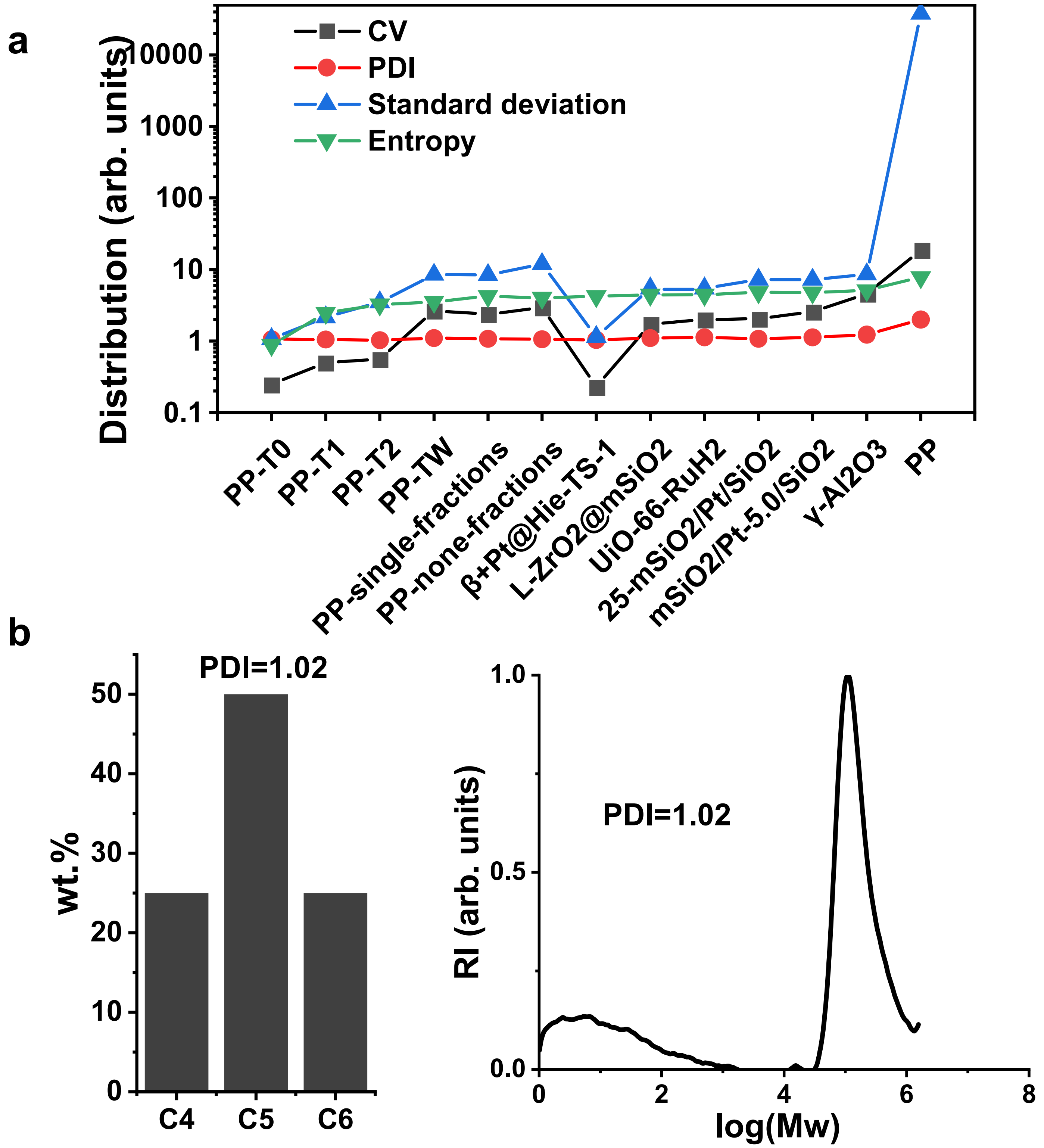

Supplementary Figure 7. (a) Distributions of PP polymer and hydrocarbon small molecules characterized by calibrated variance (CV), polydispersity index (PDI), standard deviation (σ), and entropy (S) with literature data for β+Pt@Hie-TS-1,[41] L-ZrO_2@$mSiO_2$,[40] UiO-66-RuH_2,[53] 25-$mSiO_2$/Pt/SiO_2,[55] $mSiO_2$/Pt-5.0/SiO_2,[56] and γ-Al_2O_3.[54] Lines are included for visual guidance. (b) Illustrative examples of hydrocarbons and a polymer exhibiting identical PDI values.

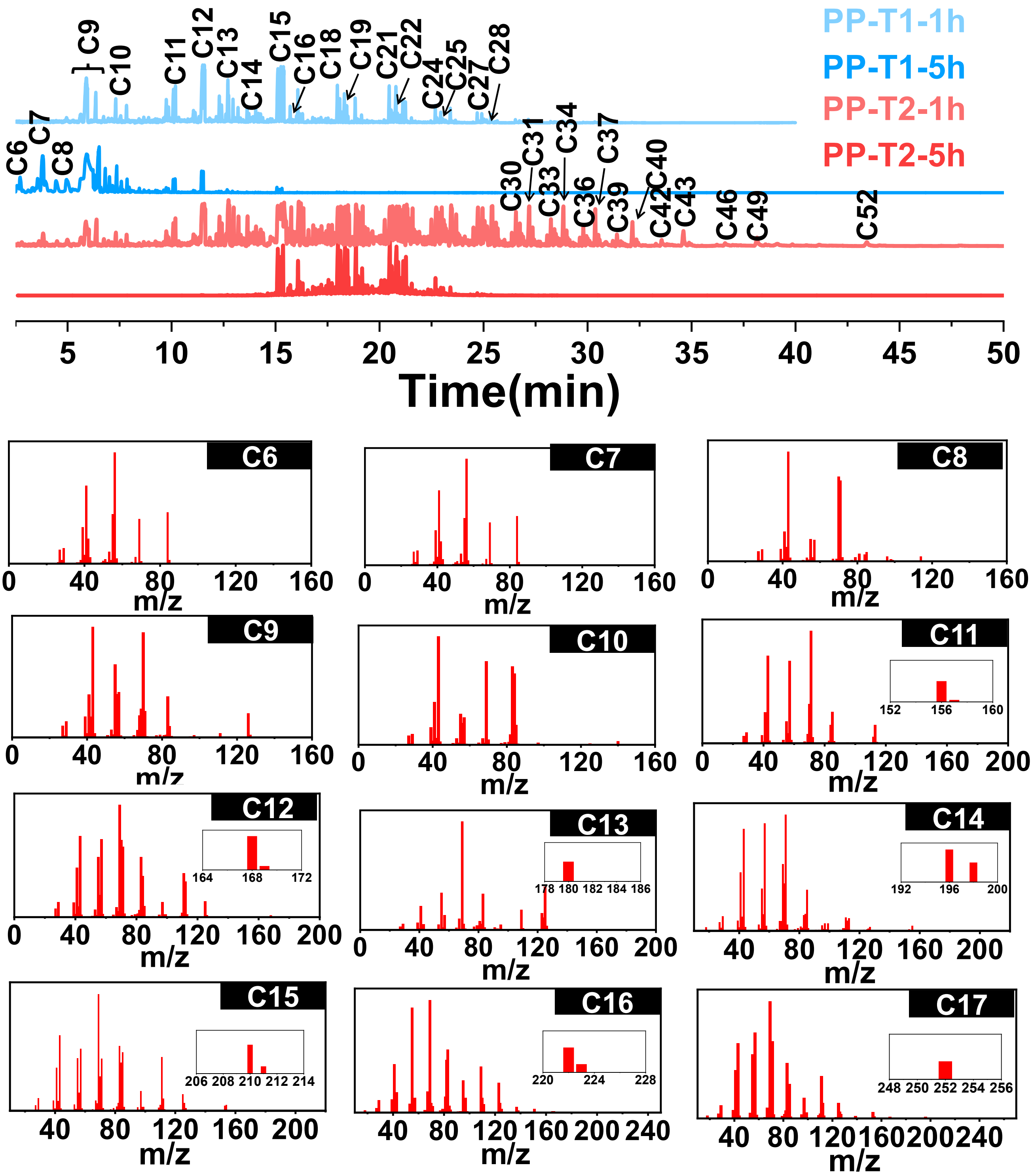

Supplementary Figure 8. GC-MS analysis of hydrocarbons on T1 and T2 after 1h and 5h reactions.

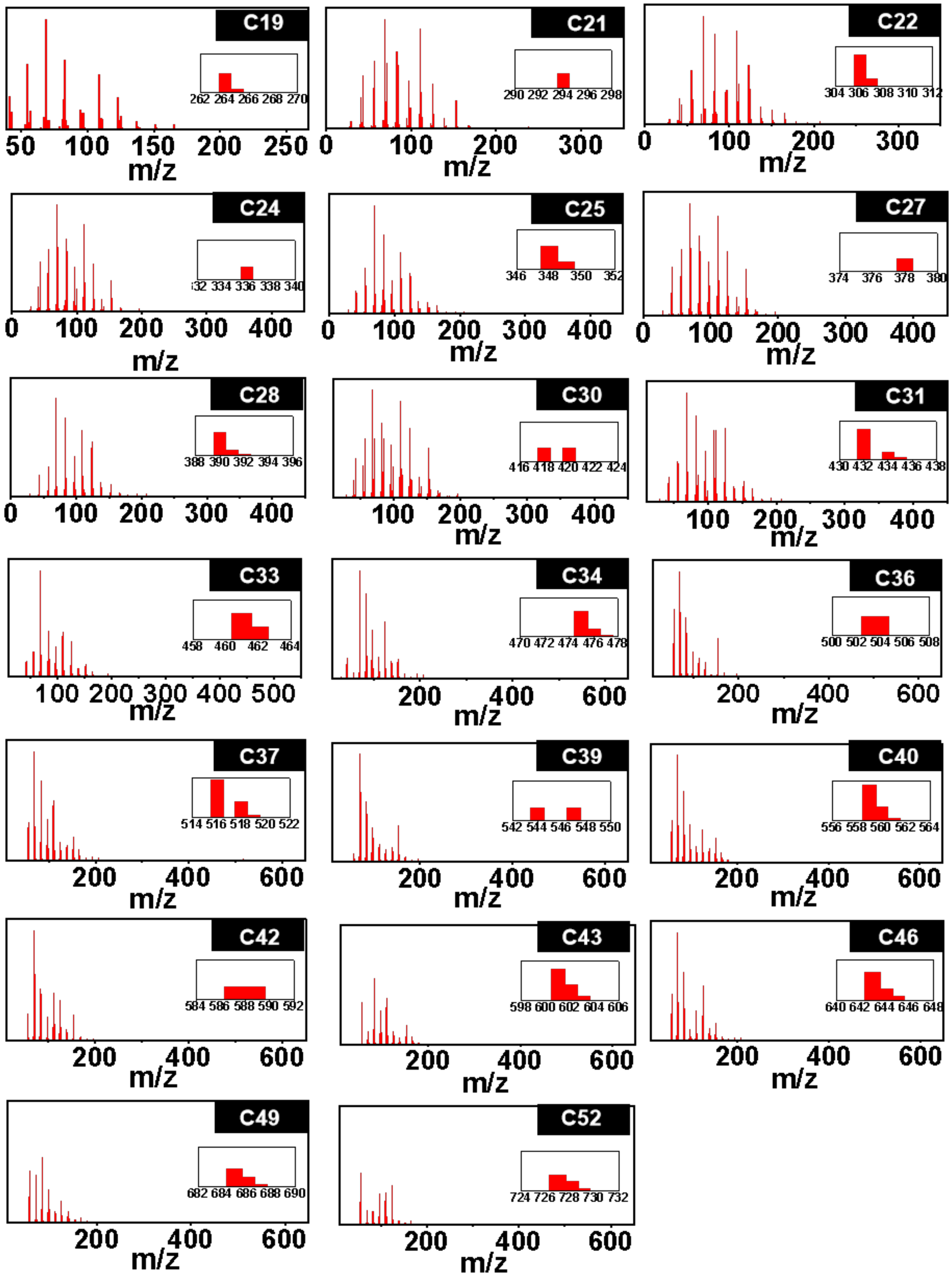

Supplementary Figure 8 (continued). GC-MS analysis of hydrocarbons on T1 and T2 after 1h and 5h reactions.

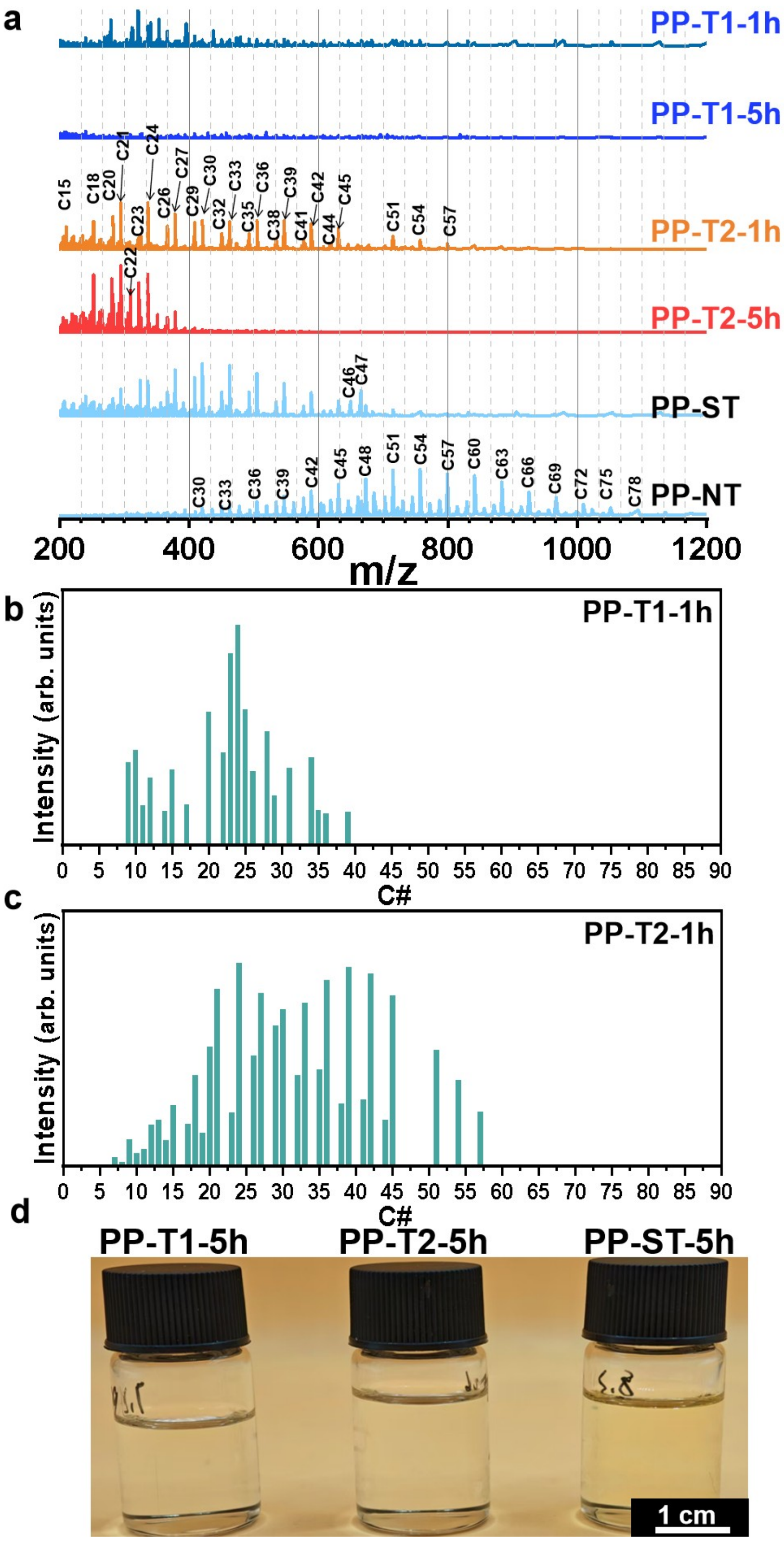

Supplementary Figure 9. (a) APCI-MS analysis of PP degradation products. The spectra exhibit the molecular weights and distributions of PP degradation products in three-fraction reactors after 1 hour (PP-T1-1h and PP-T2-1h), after 5 hours (PP-T1-5h and PP-T2-5h), PP-ST, and PP-NT-5h (Supplementary Tables 1 and 2). The spectrum of PP-T1-5h shows minimal signals, indicating that PP-T1-5h predominantly consists of hydrocarbons with chain lengths less than 15 (m/z = 210), which was reliably characterized by GC-MS (Supplementary Figure 8). A grid is used to facilitate comparison and reading across the spectra. The solvent baseline has been subtracted from the spectra. (b & c) Product carbon number distributions for PP-T1-1h and PP-T2-1h after 1-h degradation at 400°C, estimated based on GC-MS and APCI-MS. (d) Digital images of PP-T1-5h, PP-T2-5h, and PP-ST-5h.

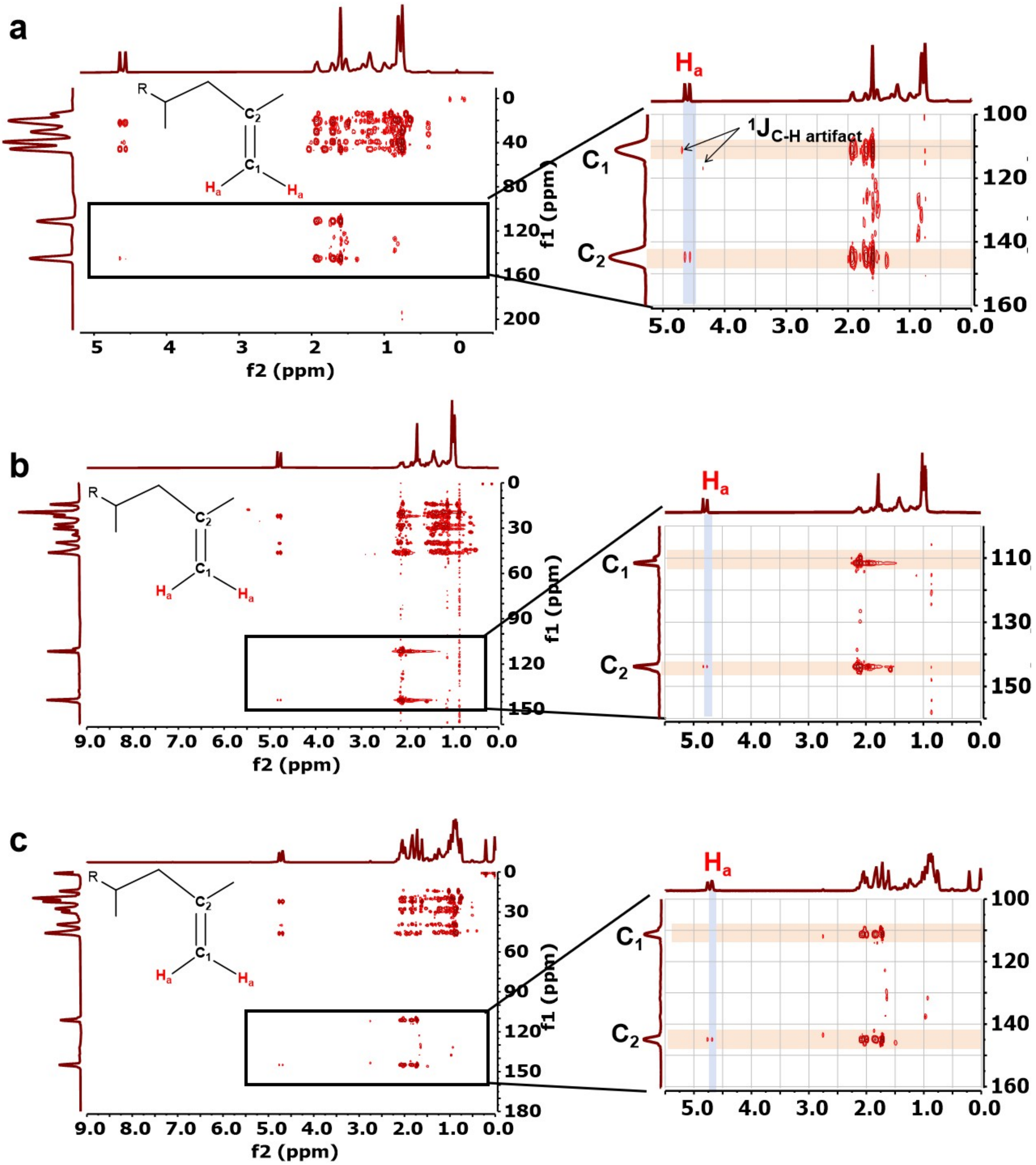

Supplementary Figure 10. HMBC-NMR spectra of (a) PP-T1-5h, (b) PP-T2-5h, and (c) PP-ST-5h in $CDCl_3$ at 22°C. The magnified views show α-alkenyl signals, as highlighted by the colored boxes. The spectra area near the C1-H_a correlation in (a) showed potential $^1J_{C\text{-}H}$ artifact peaks which are commonly observed in aliphatic and aromatic compounds.[68]

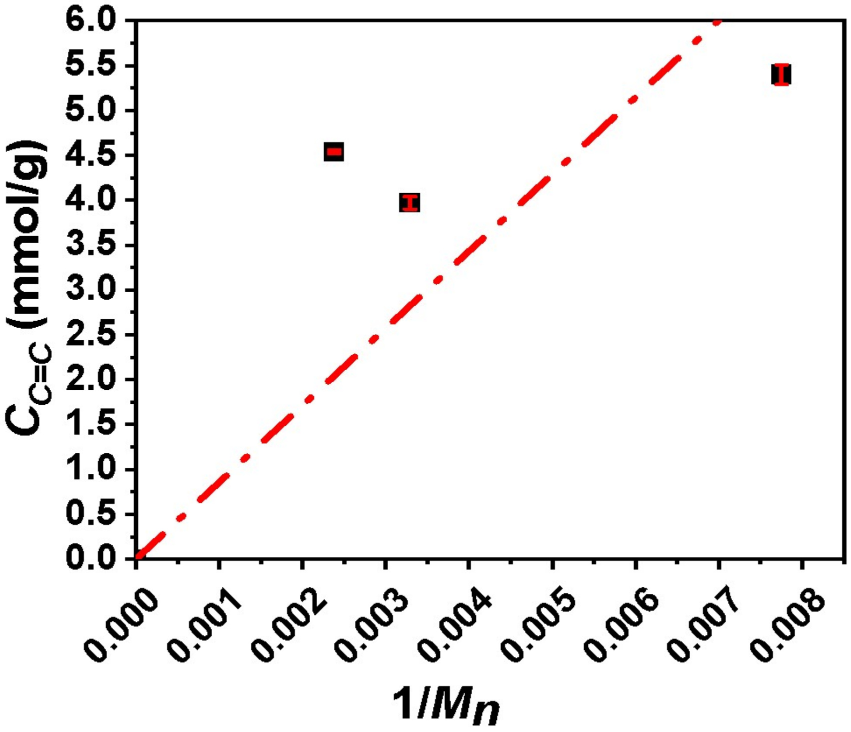

Supplementary Figure 11. Correlation between alkenyl concentration and the reciprocal of M_n. The concentrations of alkenyl groups were quantified using Supplementary Equation 2 and plotted against $1/M_n$. The slope of the linear fit, 858, closely approximates the theoretical value of 1000 for monoolefins. For comparison, a slope of approximately 2000 would be expected for diolefins. The error bar represents standard deviation.

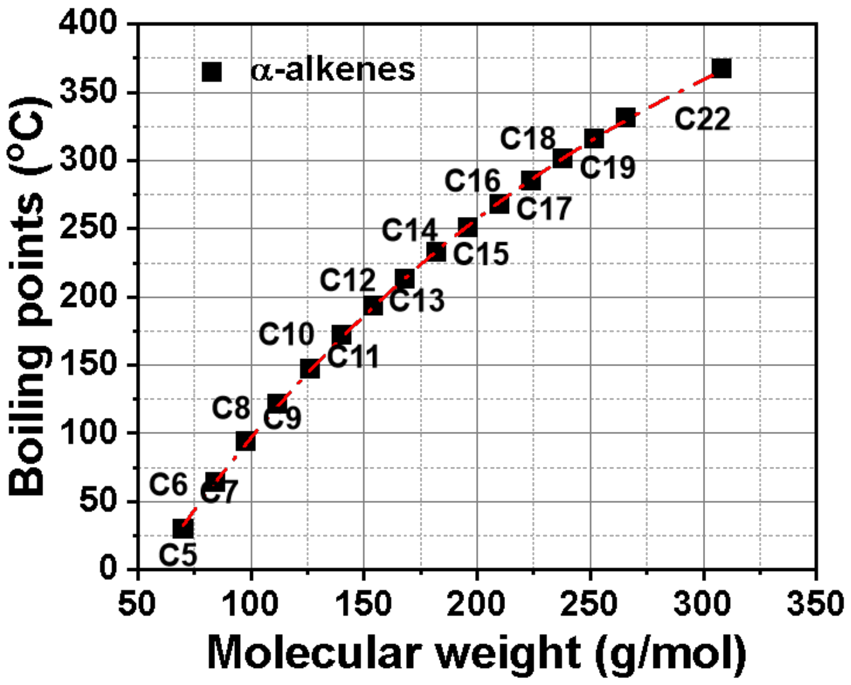

Supplementary Figure 12. Correlation between the boiling points and molecular weights of α-alkenes. The boiling point data were sourced from the Engineering Toolbox database.[69] Each data point is labeled with the corresponding carbon number.

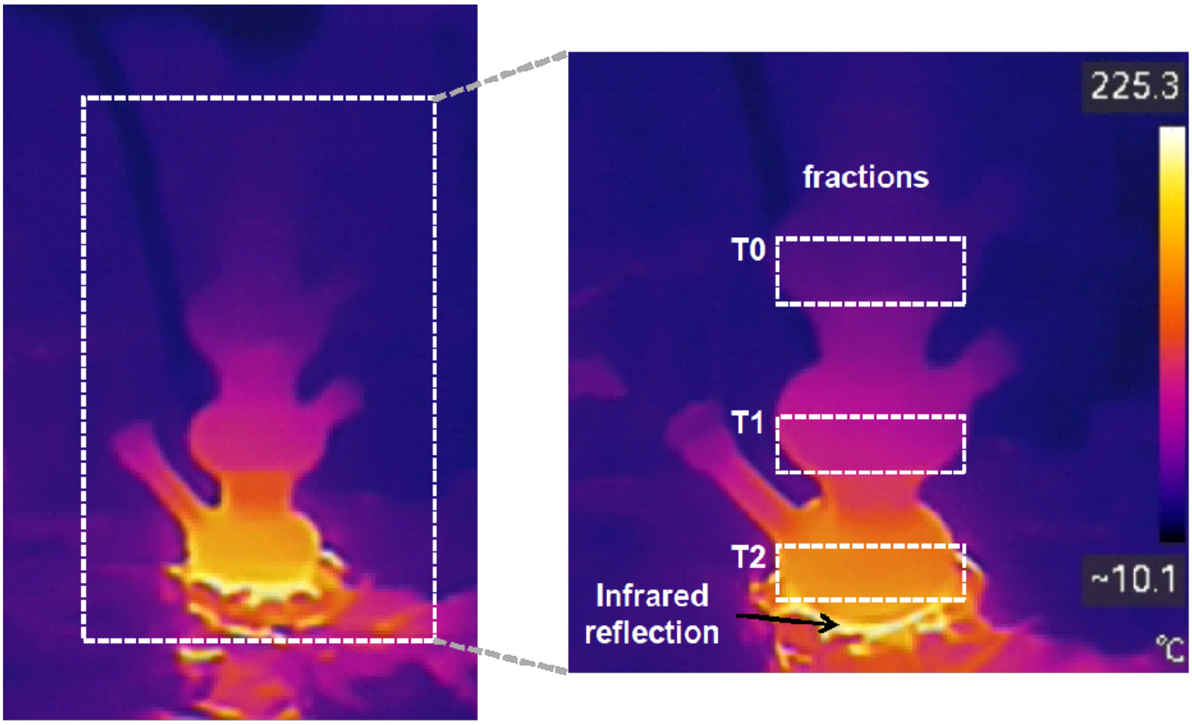

Supplementary Figure 13. Infrared thermographic imaging of a three-fraction reactor during polymer degradation. The left panel represents the overall reaction environment, while the right panel shows a magnified view. White boxes highlight fractions T0, T1, and T2. The thermal gradient bar indicates the surface temperature distribution on the reactor. Five temperature points were sampled from each box, averaged as the T_s of a fraction (Supplementary Table 1).

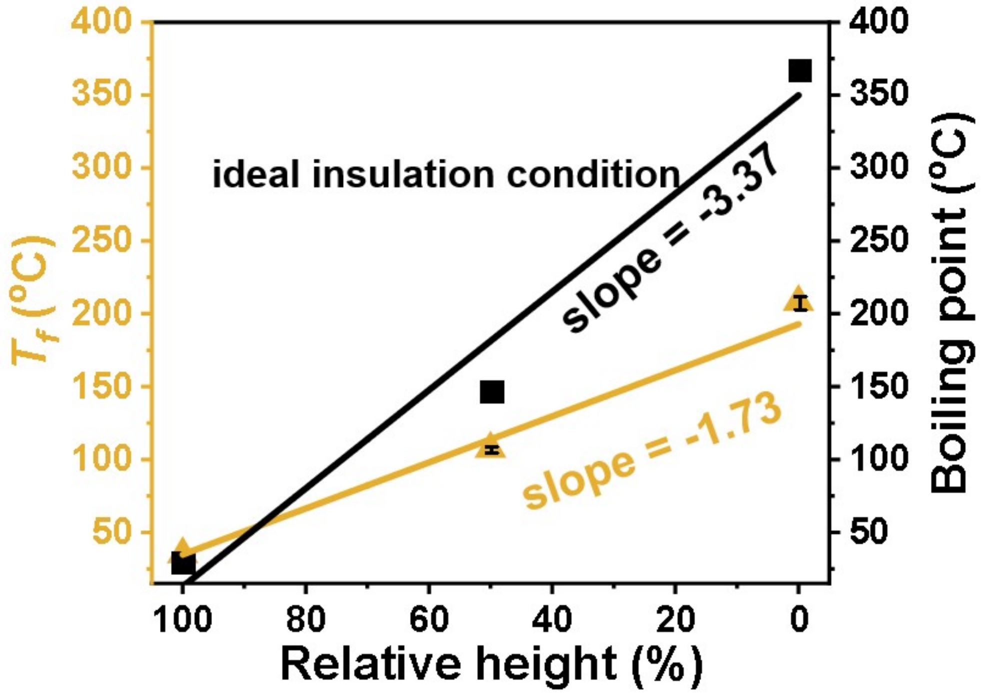

Supplementary Figure 14. Determination of the empirical correction factor k = 1.947. The black line represents the tray temperature under ideal insulation conditions, while the yellow line shows the tray temperature from experimental measurements, as extracted from Figure 2a and Supplementary Table 1. The error bar represents standard deviation.

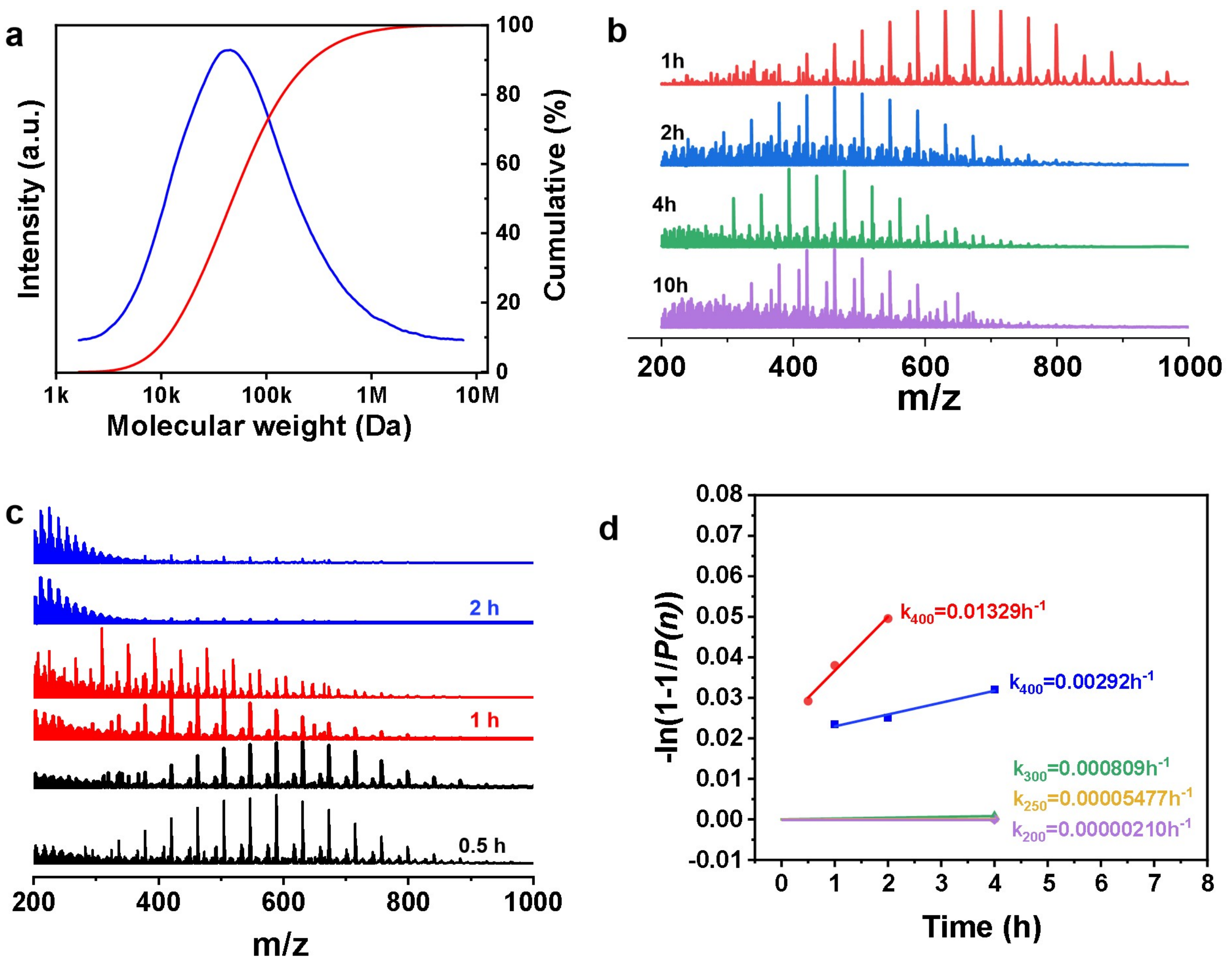

Supplementary Figure 15. Study of PP chain scission kinetics. (a) HT-GPC curves of laboratory-grade PP (M_n = 87 kDa, M_w = 387 kDa, PDI = 4.72); (b) and (c) APCI-MS of PP degradation at 360 and 400°C, respectively; (d) kinetic fitting of PP degradation at 360 and 400°C and their kinetic constants. The kinetic constants for 300 , 250, and 200°C (dashed lines) were predicted for reference.

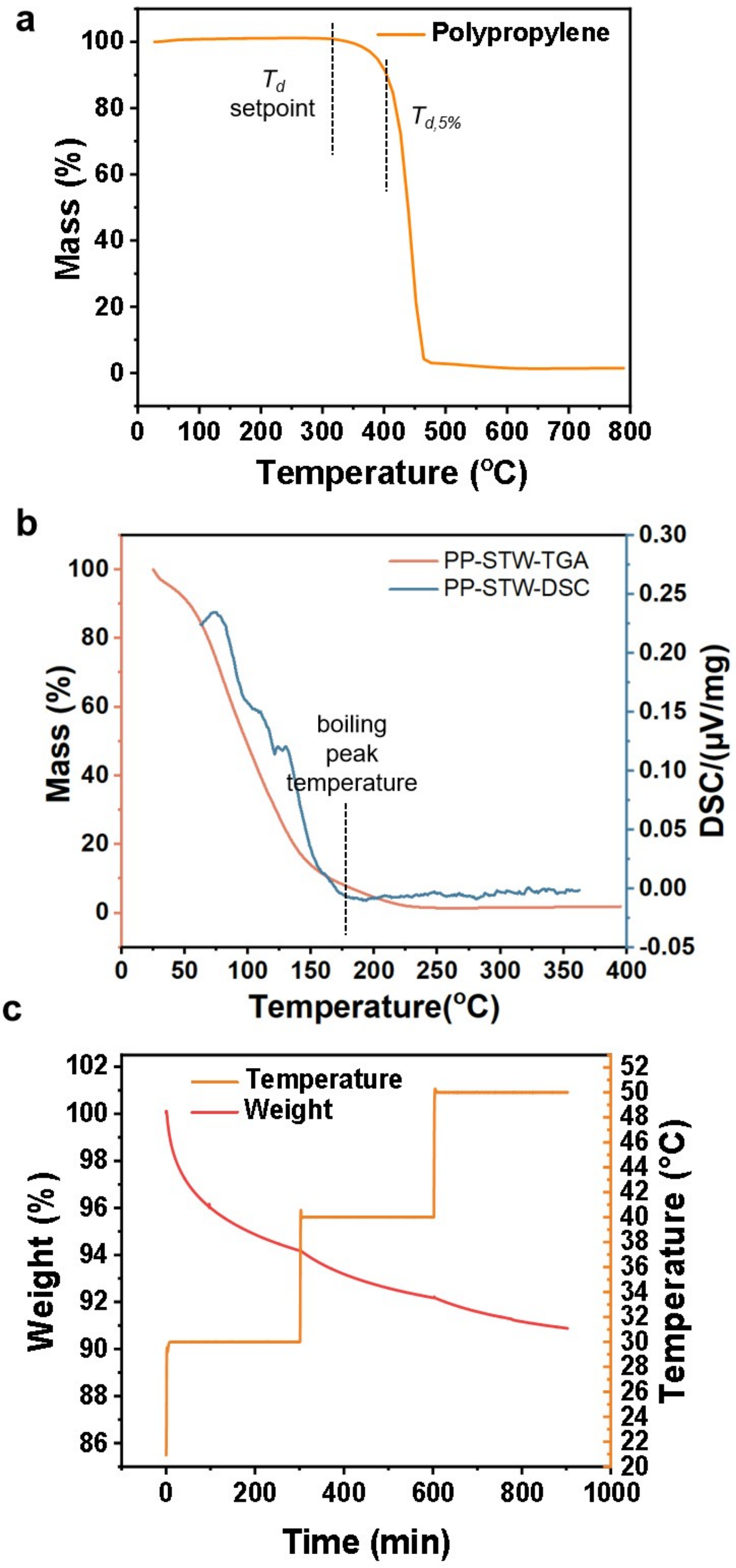

Supplementary Figure 16. Experimentally determined properties for ASPEN simulations and process design. Thermal properties of (a) PP and (b) PP-ST-5h measured by TGA and DSC. (c) Water content in the surfactant was determined by isothermal mass loss at 30, 40, and 50°C, equilibrated near 90 wt.%. Therefore, 5 h is adequate for net content ~ 95% at 30°C.

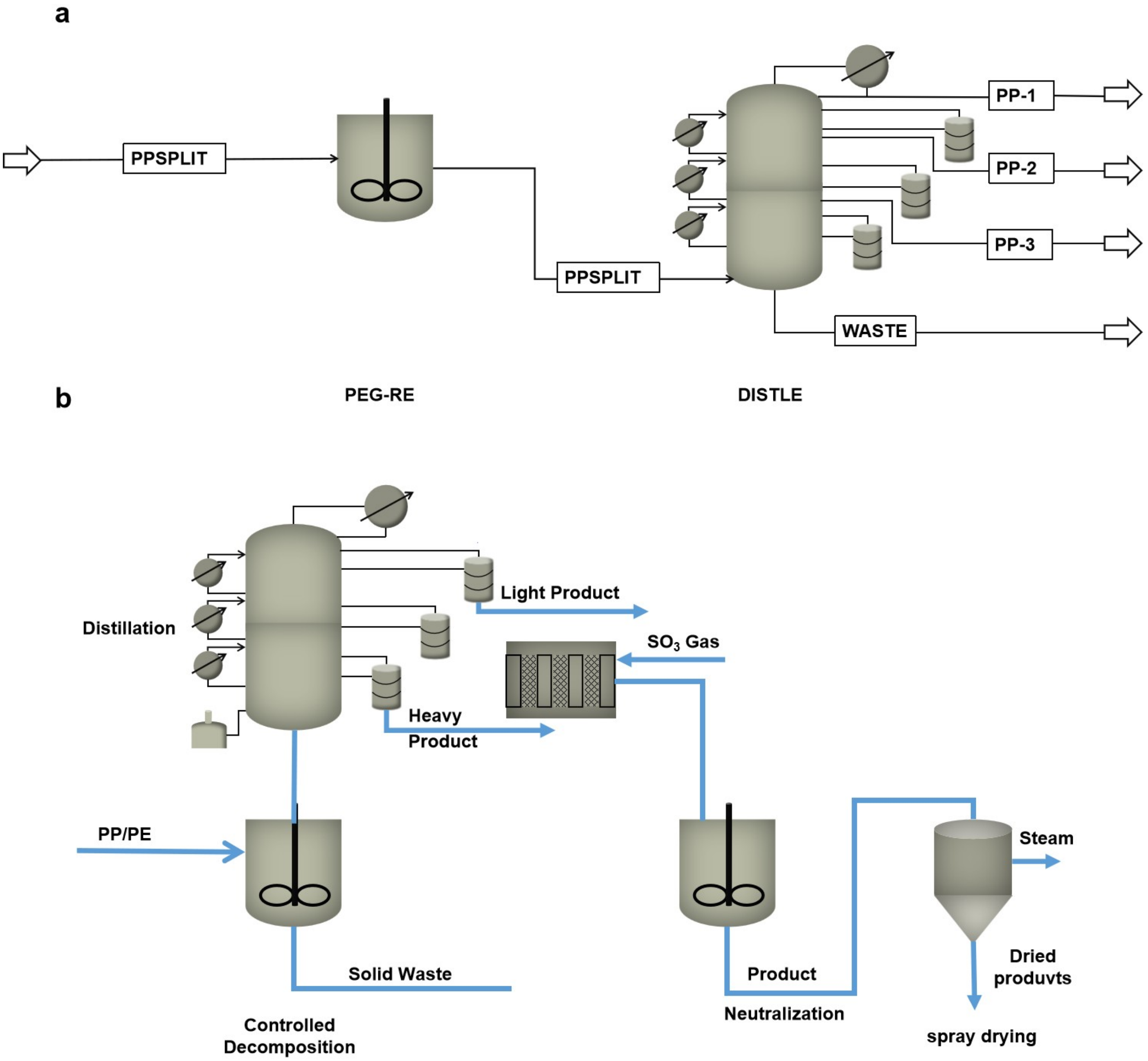

Supplementary Figure 17. Process flow diagrams for ASPEN simulations. (a) PFD of fractionated degradation and (b) upcycling generated by ASPEN software.

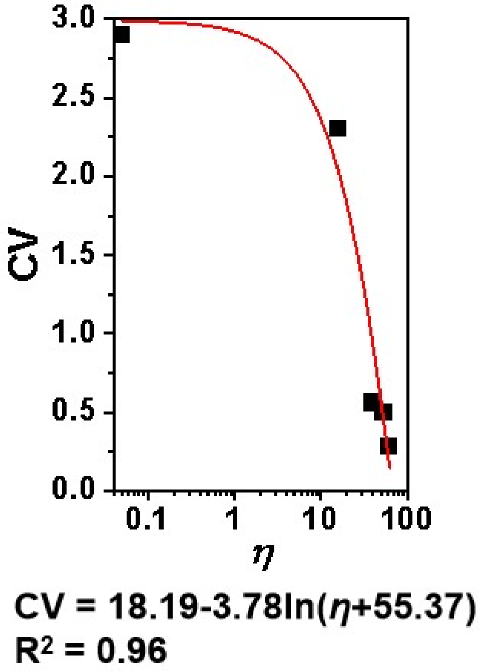

Supplementary Figure 18. Empirical correlations of CV with η. Logarithmic fitting of η with CLD descriptor, showing that η is a critical parameter controlling the distribution range. Data were extracted from Supplementary Table 8 and 9.

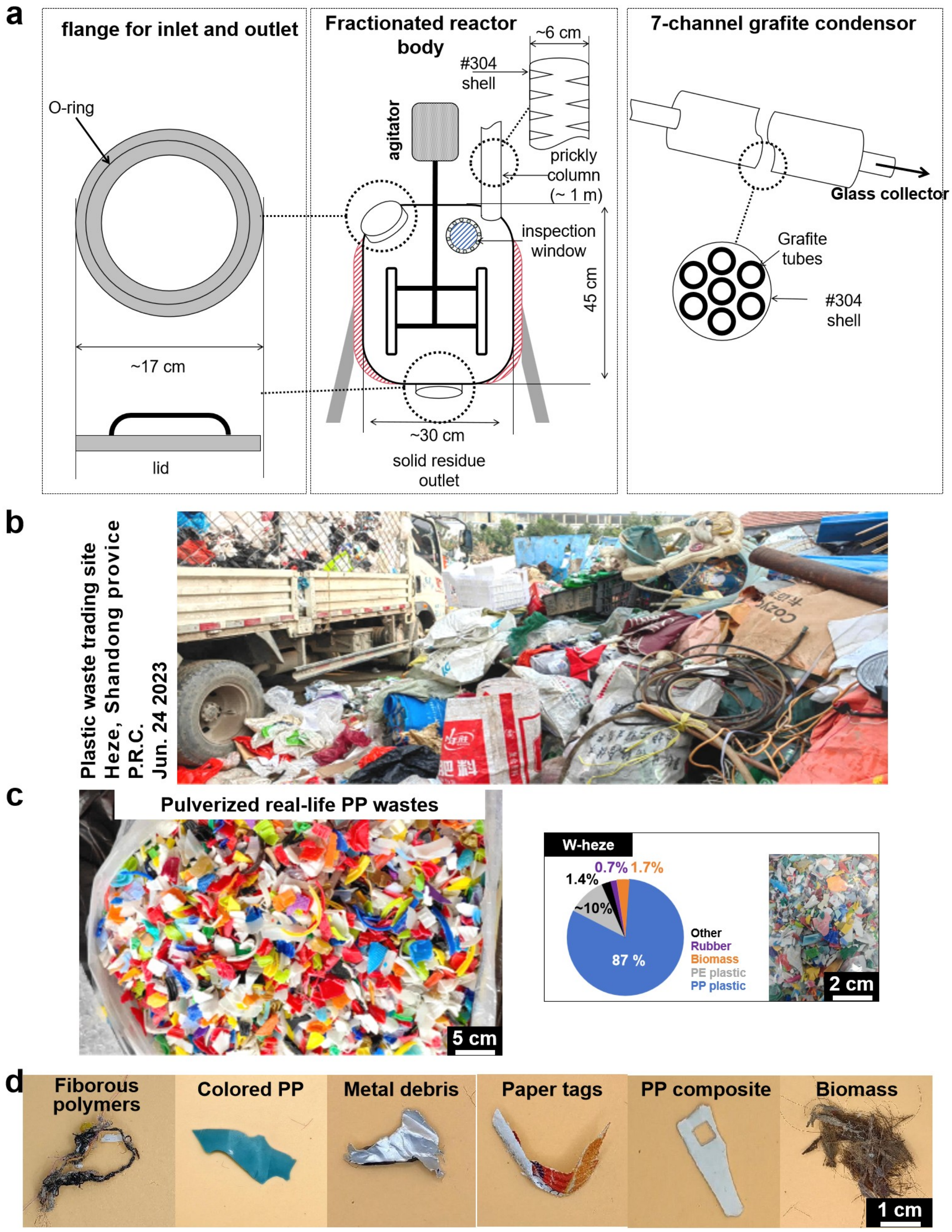

Supplementary Figure 19. Scale-up experiments using real-life plastics. (a) Schematic diagram of the scale-up reactor, featuring flanges for inlet and outlet (left), a fractionated reactor body equipped with an agitator, inspection window, and prickly column (center), and a 7-channel graphite condenser connected to a glass collector (right). Dimensions and primary components are labeled accordingly for clarity. The reactor body is constructed with a #304 stainless steel shell and includes a solid residue outlet at the base. The scale-up reactor was cleaned using ethanol before use. (b) The source and time of procurement of real-life PP wastes: images show PP wastes collected from a plastic waste trading site in Heze, Shandong Province, China, collected on June 24, 2023. The weights were measured using a platform balance (±0.1 kg). (c) Pulverized real-life PP wastes from the collection site in (b) and it composition. The mass ratio of PE was roughly estimated based on Supplementary Figure 23b. (d) Compositional analysis of real-life PP wastes: images show various waste components including fibrous polymers, colored PP, metal debris, paper tags, PP composites, and biomass.

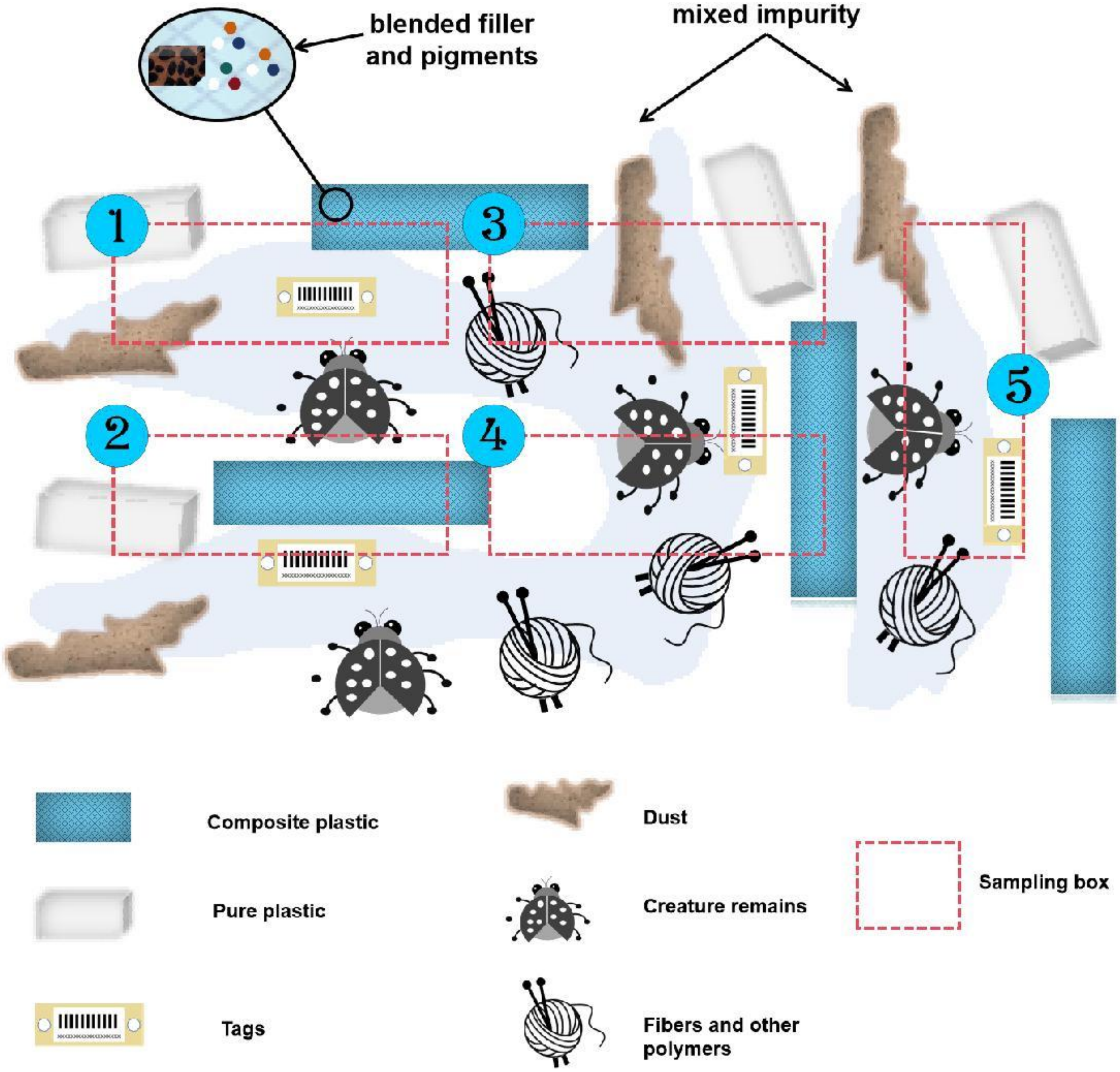

Supplementary Figure 20. Complexity of real-life plastic wastes. This figure illustrates the complex composition of an real-life plastic waste sample, which includes various impurities such as dust, tags, biomass, and other polymers. These impurities are categorized into mixed and blended impurities. The blue region indicates the domain of mixed impurities. The complexity of real-life plastic waste prevents a single portion from being representative. For example, the portions labeled 1 to 5 each contain different ratios of plastic. The portion 5 containing no plastic at all. Therefore, for the accurate investigation of real-life plastic waste, it is essential to conduct scale-up experiments or large-scale sampling to obtain representative samples.

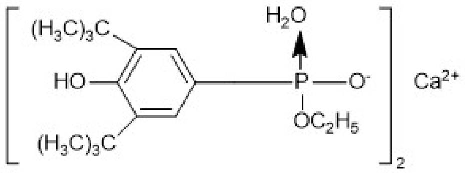

AO-13

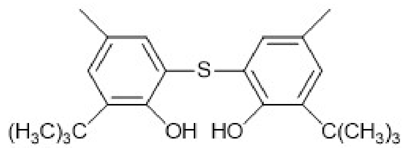

AO-27

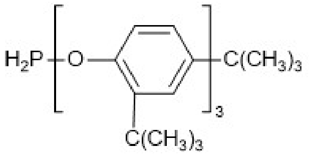

P-1

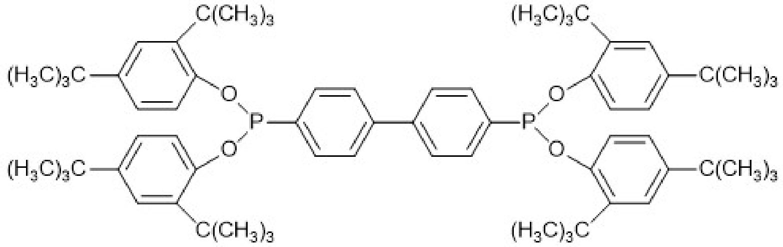

P-2

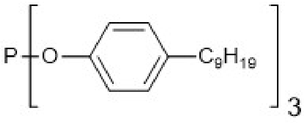

P-3

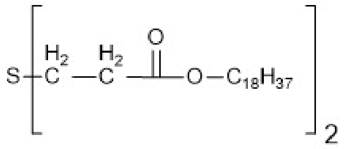

S-1

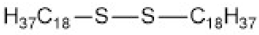

S-2

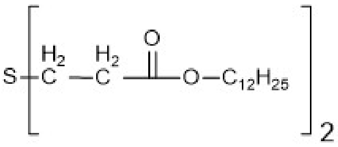

S-3

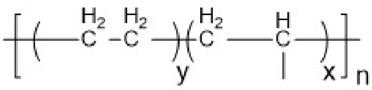

EPDM

Cadmium Yellow: (Cd, Zn)S
Cadmium Red: Cd(S.Se)
Ultramarine Blue: ($Na_{6\sim8}Al_6Si_6O_{24}S_{2\sim4}$)

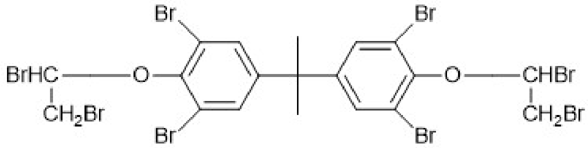

TBBPA-BDBPE

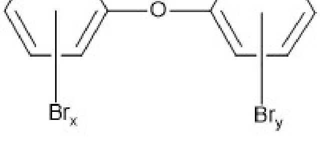

Decabromodiphenyl ether
x+y=10
Octabromodiphenyl ether
x+y=10
Pentabromodiphenyl ether
x+y=10

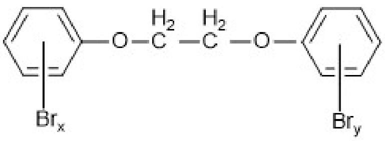

Thiobromodiphenoxyethane
x+y=6 (1,3,5)

Supplementary Figure 21. Examples of potential additives.[44,45]

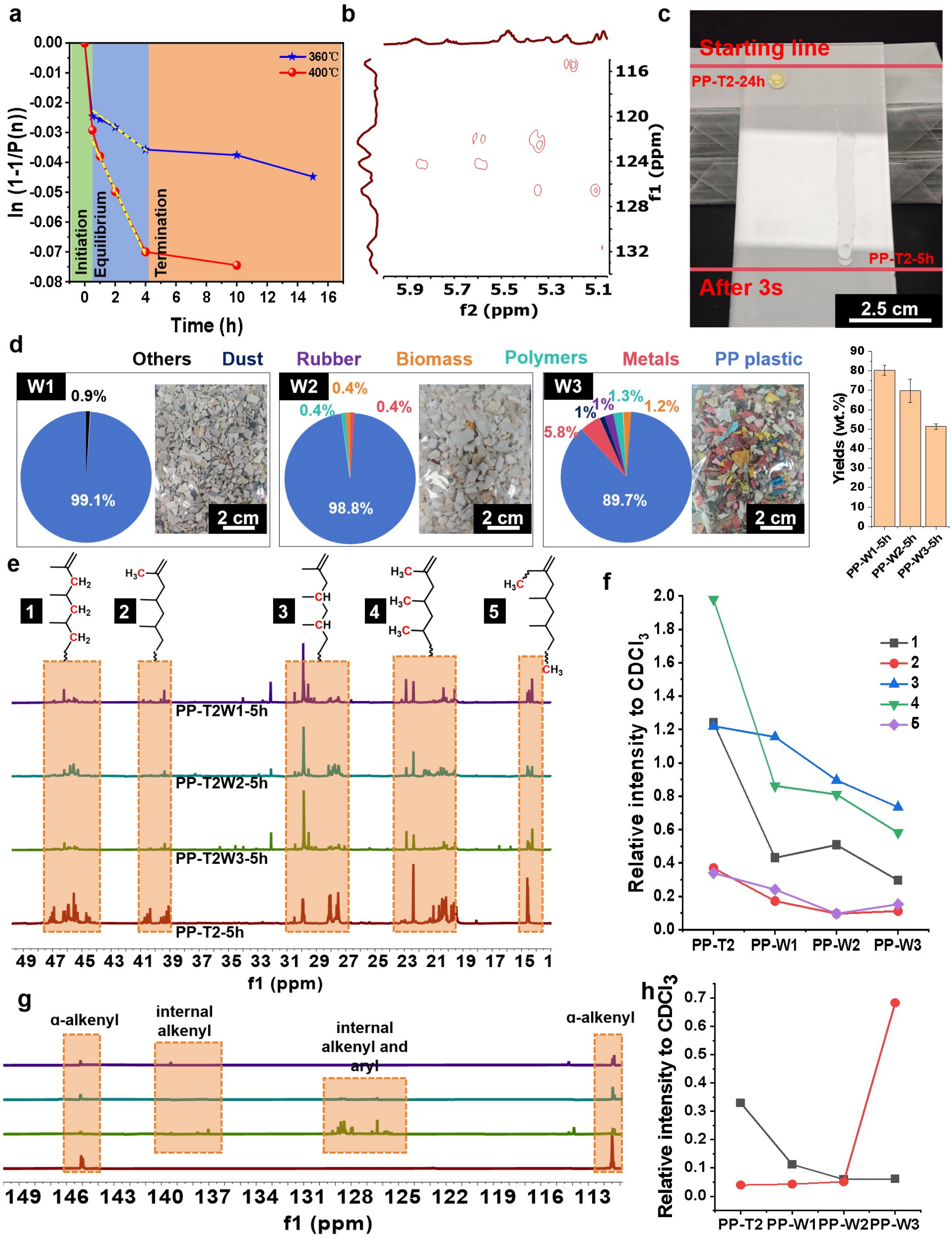

Supplementary Figure 22. Side reactions and purity effects on olefin chemistry. (a) Fractionated degradation kinetics under simulated conditions (400°C, ambient pressure) using a Schlenk flask. (b) Enlarged view of Supplementary Figure 10b highlighting aryl and internal alkenyl correlations. (c) Flow tests of PP-T2-5h and PP-T2-24h on silicone gel plate. PP-T2-24h shows yellowish color and higher viscosity than PP-T2-5h, suggesting polymerization. (d) Standardized purity analysis of Jieshou samples W1-W3 and their yields in laboratory-scale fractionated degradation using three-fraction reactors.[7] The other category includes minor PET plastics, PVC plastics, and undetermined components. The error bar represents standard deviation of at least 3 parallel experiments. *NOTE, PP or PE plastics are PP polymer with additives.* (e,f) ^{13}C NMR (10-50 ppm) spectra of fractionated products in $CDCl_3$ at 22°C from W1-W3 and PP-T2-5h using three-fraction reactors; structural variations in PP-T2-5h, T2W1, T2W2, and T2W3 indicate purity-dependent trends. (g) Spectral evolution in 110-150 ppm region showing α-alkenyl and internal alkenyl (including aryl) evolution. (h) Evelution of internal alkenyl and aryl groups (red) and α-alkenyl (black line).

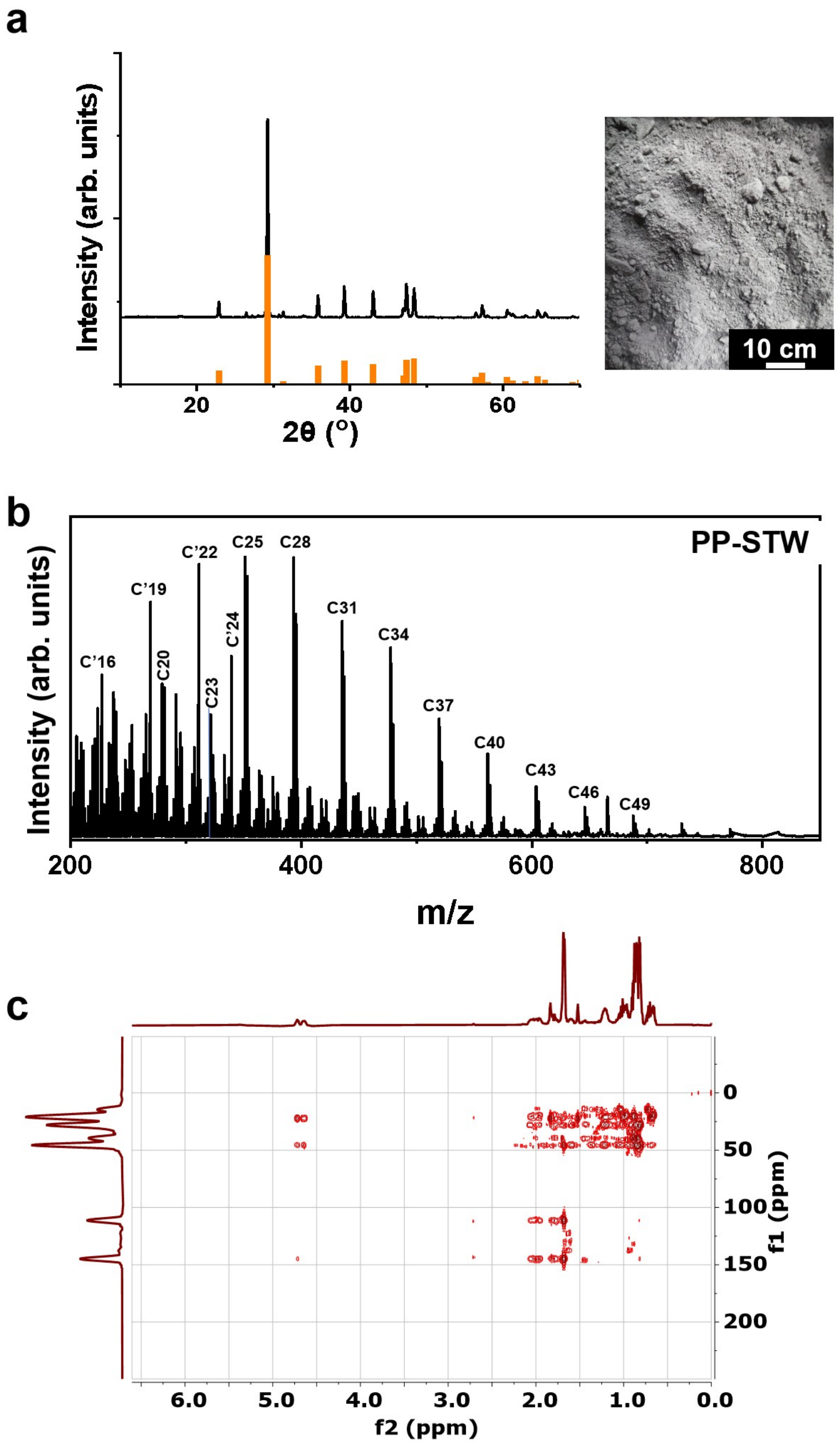

Supplementary Figure 23. Characterization of PP-STW-6h and solid residue. (a) PXRD pattern of solid reside and simulated pattern for $CaCO_3$. Insert shows digital image of solid residue after degradation. (b) APCI-MS spectrum of PP-STW-6h, where "C xx" and "C' xx" denote alkenes and alkanes, respectively. The mass ratio of PE can be roughly estimated based on the spectrum and the following assumptions: 1. molar ratio of alkane is 3.5 mol%; 2. yields of alkane from PE degradation was assumed to be 40 mol%; 3. the average molar mass of the alkane portion is ~ 350 kg/mol; 4. the average molar mass of the alkene is ~ 350 kg/mol; 5. other impurity are inert. (c) HMBC-NMR spectrum of PP-STW-6h in $CDCl_3$ at 23.5°C.

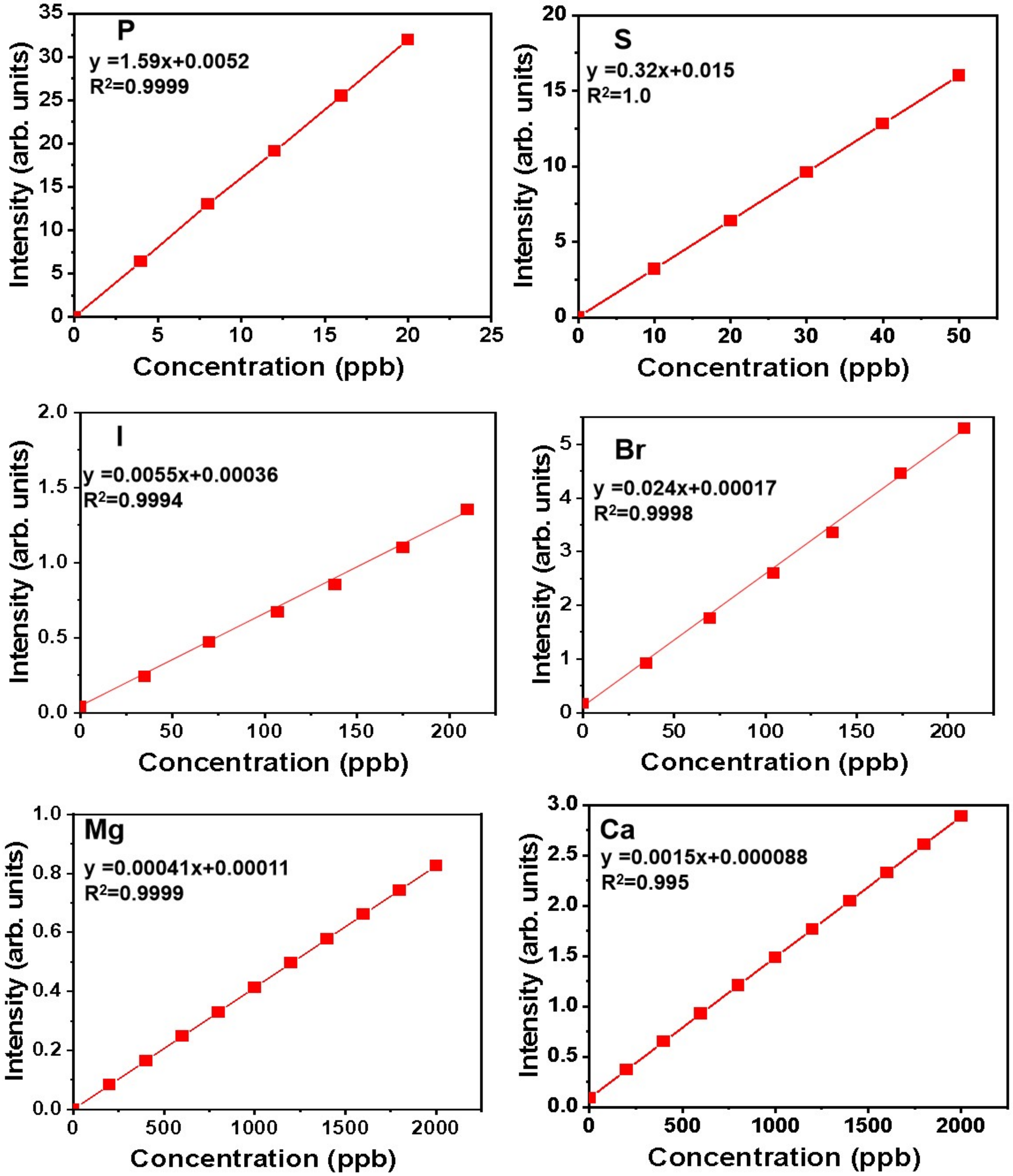

Supplementary Figure 24. Standard curves of P, S, I, Br, Mg, and Ca for ICP element concentration analysis.

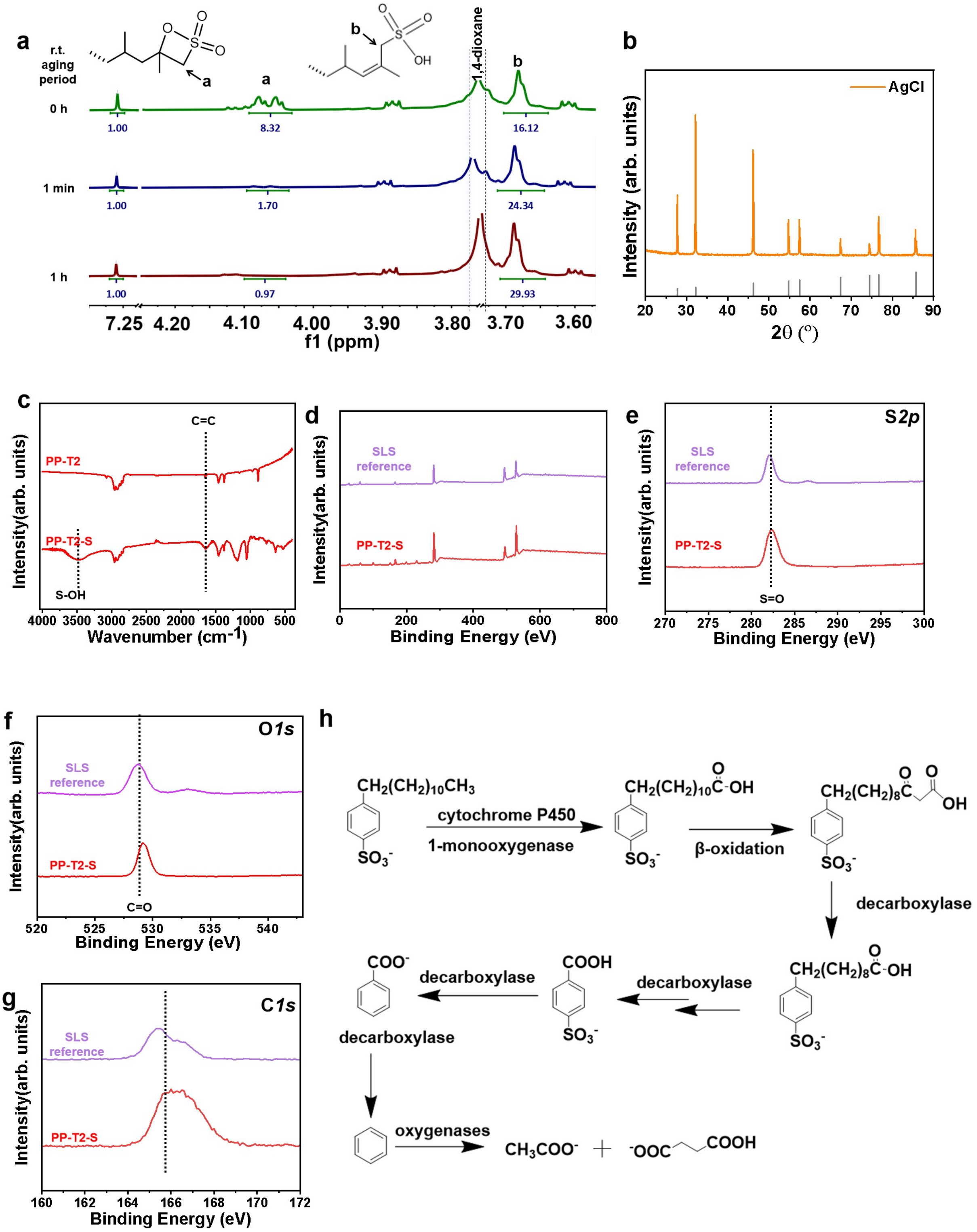

Supplementary Figure 25. Characterization of sulfonation intermediates and products. (a) Evolution of sulfonated product structures at ambient temperature, as indicated by ^{1}H NMR in $CDCl_3$. (b) PXRD pattern of precipitates formed in $AgNO_3$ solution during sulfonation and simulated pattern of AgCl. The composition of the sulfonation exhaust gases was characterized using chemical adsorption method. The exhaust gas was purged into an aqueous $AgNO_3$ solution, generating white precipitate. The precipitate, characterized by PXRD, displayed spectrum lines corresponding solely to AgCl, indicating that the primary component of the exhaust gas is HCl, with negligible amounts of SO_2 and SO_3. (c-g) FTIR and XPS spectra of α-alkenes, sulfonate, and a sodium lauryl sulfate reference. The presence of S-OH and S=O signals confirmed the successful conversion of alkenes to sulfonates. In (c), the C=C signals exhibit broadening due to the formation of internal alkenes, as evidenced by HT-HMBC-NMR spectra (Figure 3a and Supplementary Figure 23). (h) A typical bio-degradation pathway of sulfonate compounds.[52]

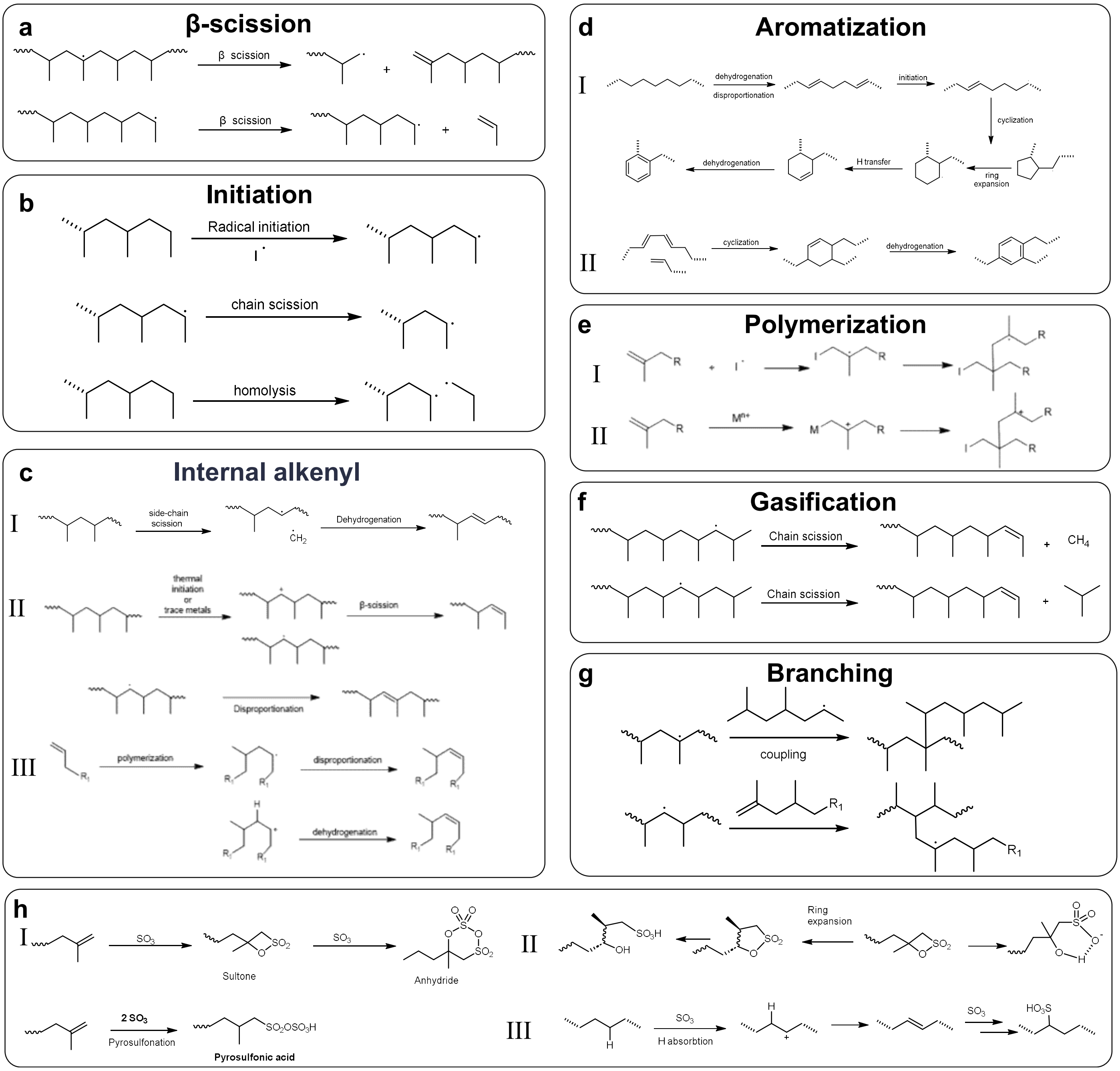

Supplementary Figure 26. β-scission, sulfonation, and other potential side reactions.

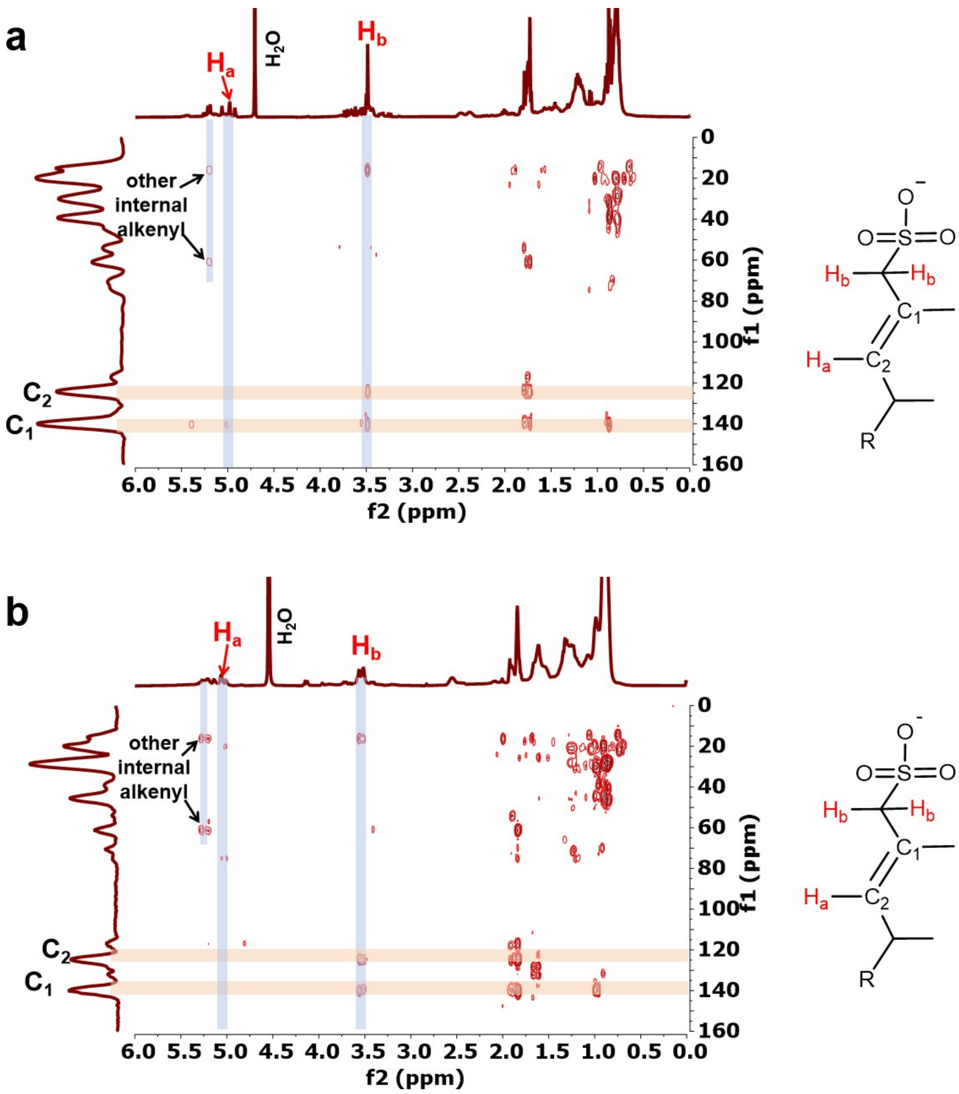

Supplementary Figure 27. HT-HMBC-NMR spectra of (a) PP-T1-5h-S and (b) PP-STW-6h-S. The sulfonates were dissolved in D_2O and characterized at 50°C to enhance solubility and signal resolution. Similar to the spectra shown in Figure 3a, both spectra exhibit signals corresponding to alkenyl sulfonates. The sulfonation reaction resulted in the formation of new alkenyl groups other than the β-alkenyl, attributed to a dehydrogenation reaction induced by SO_3, a potent Lewis acid.[34]

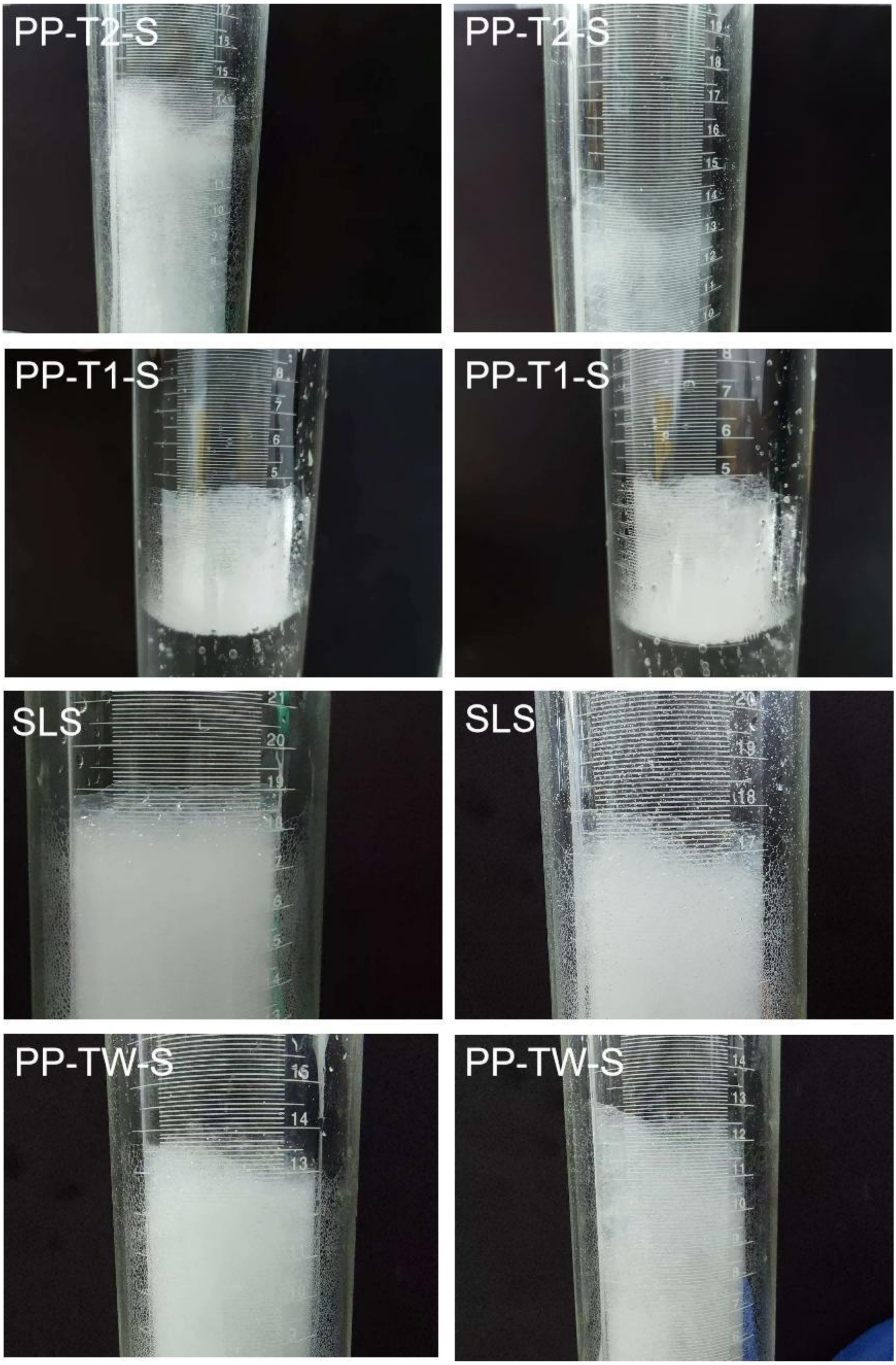

Supplementary Figure 28. Digital images of foaming height determined by Ross-Miles Method.

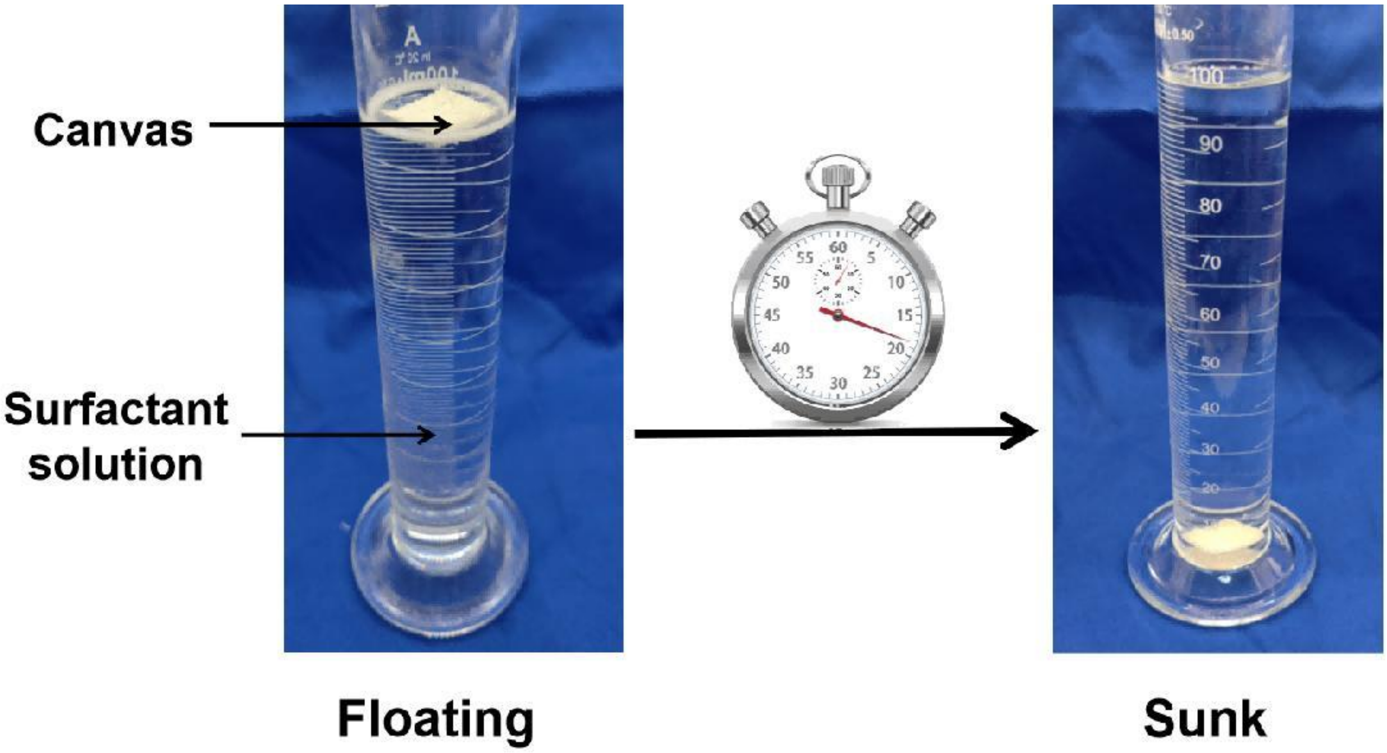

		Deionized water	PP-T1-S	PP-T2-S	SLS
Sinking time (s)	Exp1	60	20	8	12
	Exp2	63	27	8	11
	Exp3	67	27	9	11
	Exp4	75	23	10	13
	Average	66.3	24.3	8.8	11.8

Supplementary Figure 29. Wettability assessment using the cloth disc method. A cotton disc (0.5 cm × 0.5 cm) was placed on the surface of the test liquid in a 100 mL graduated cylinder. The stopwatch was started immediately upon placement of the disc and the time was recorded when the disc reached the bottom of the cylinder. Each surfactant and the blank control were measured in quadruplicate. The wetting of PP-T2-S solution was significantly faster than that of PP-T1-S and slightly faster than the SLS reference solution.

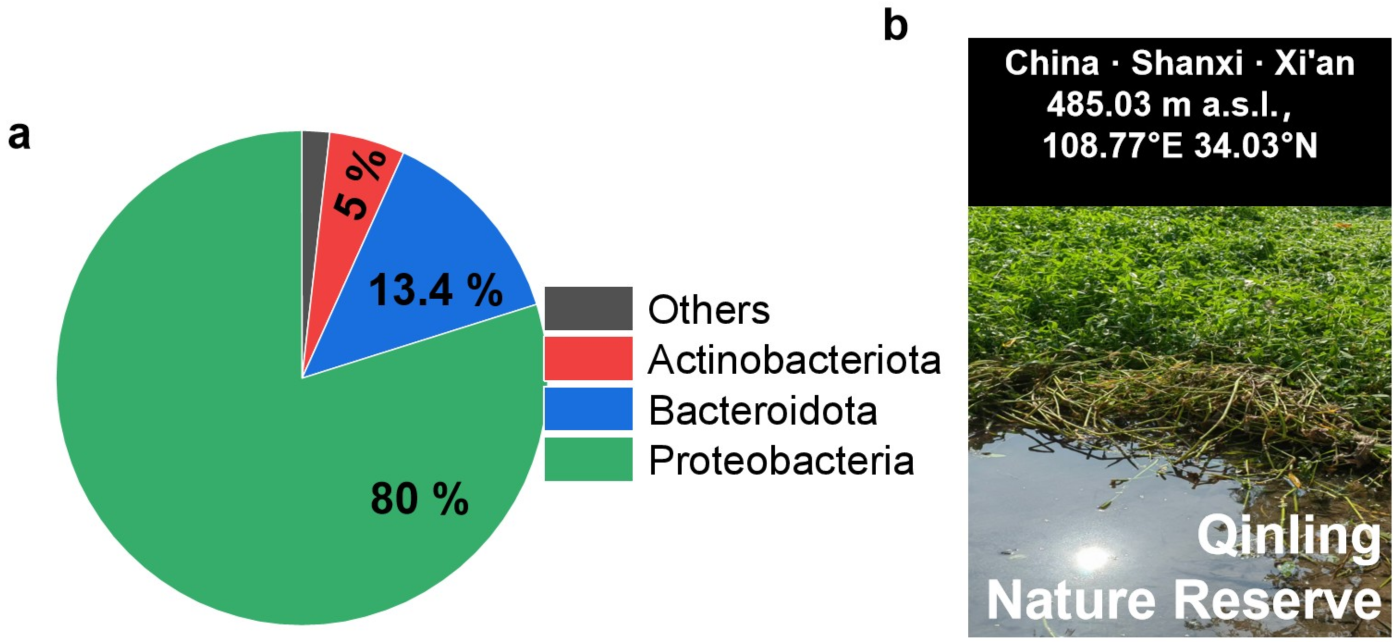

Supplementary Figure 30. Natural sludge collected from national reserve in Qinling mountain. (a) Microbial analysis of the natural sludge. The PCR amplification analysis showed dominantly *Proteobacteria* strain. (b) Geological location of the sludge sampling point.

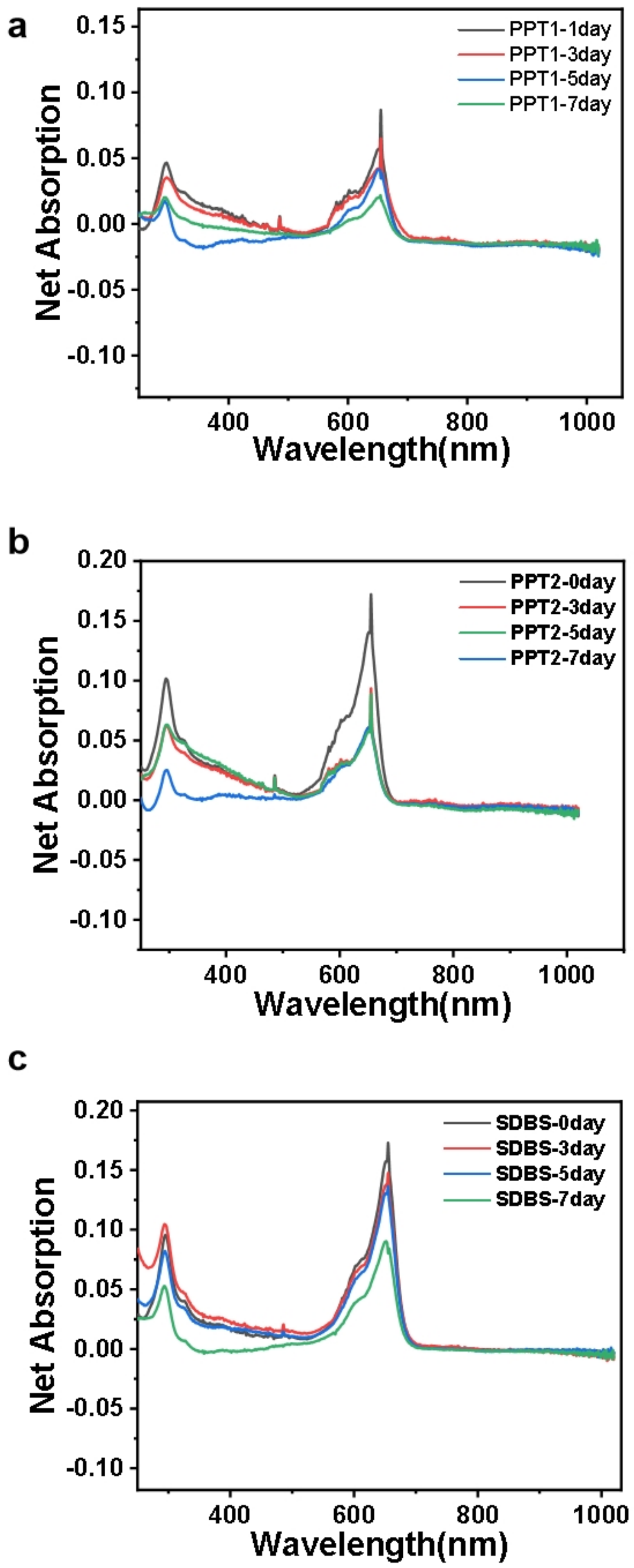

Supplementary Figure 31. UV-vis spectroscopy characterization of surfactant concentrations, including (a) PP-T1-5h-S, (b) PP-T2-5h-S, and (c) SDBS, in sludge solutions. Baselines were subtracted.

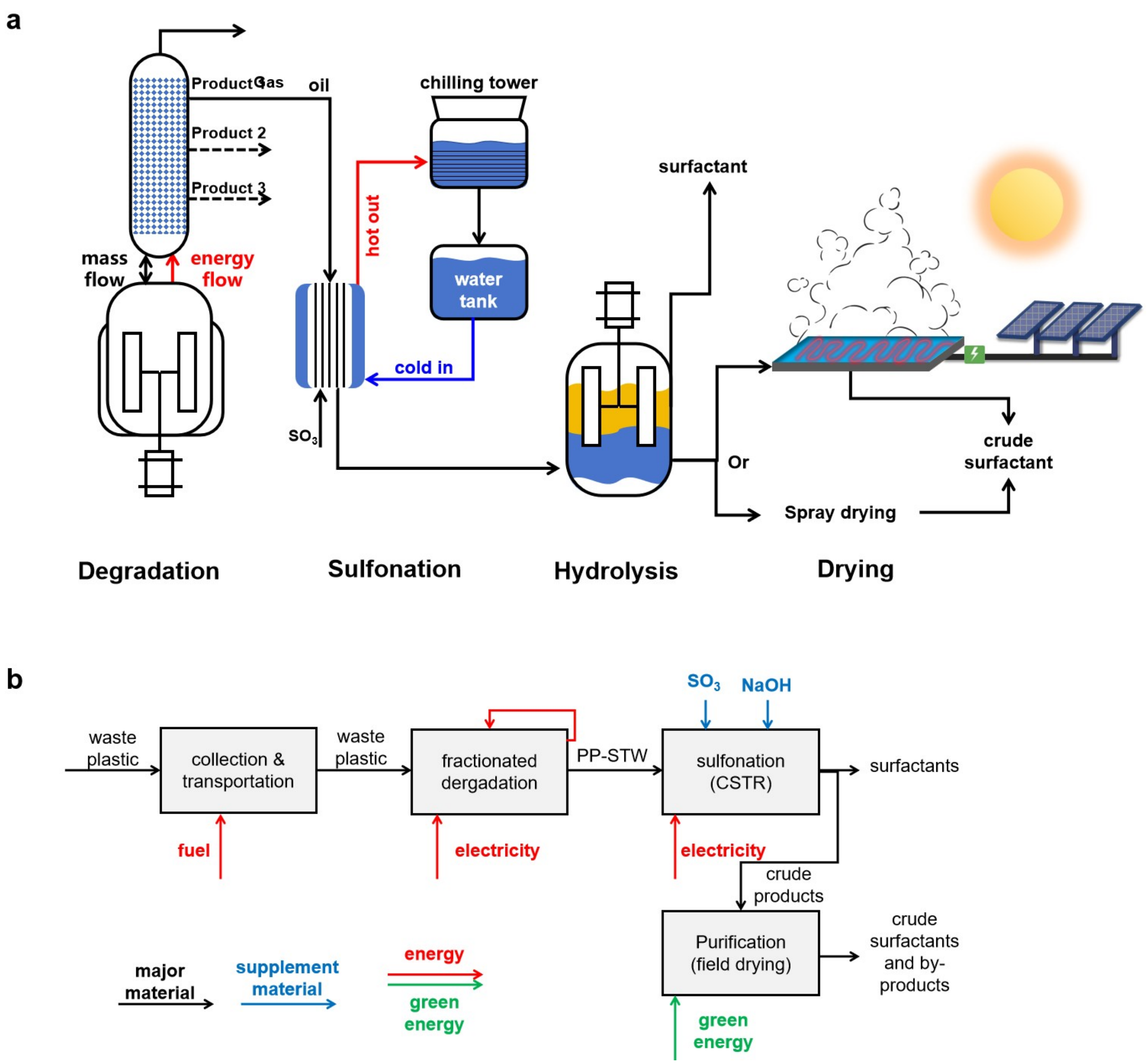

Supplementary Figrue 32. Flow diagram and block flow chart for LCA. (a) Schematic flow diagram of PP upcycling, including degradation, sulfonation, hydrolysis, and drying (solar drying and spray drying). (b) System boundary and material inventory of life cycle assessment of carbon emissions. wp: waste PP.

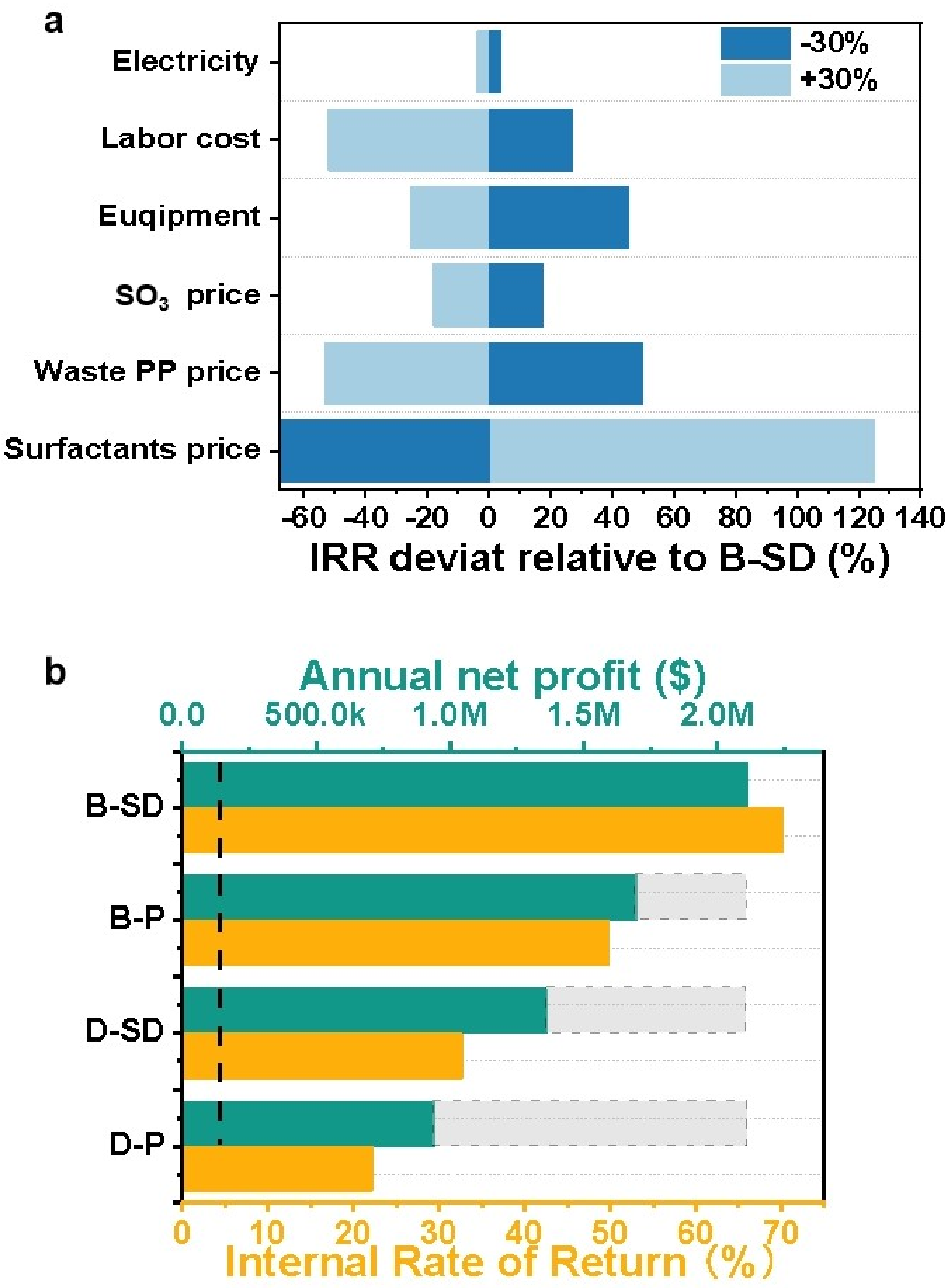

Supplementary Figure 33. Sensitivity analysis and profitability comparisons. (a) Tornado diagram showing the sensitivity of B-SD profitability to variations in electricity price, labor cost, equipment cost, SO_3 price, PP waste price, and surfactant price. Deviations are shown at ±30% relative to the base costs. (b) Comparative analysis of profitability across different scenarios: B-SD, B-P, D-SD, and D-P. The boxes highlight the profit loss relative the B-SD scenario.

Supplementary Table 1. Degradation products in fractionated and none-fraction reactors.

	Yields (wt. %)	T_f (°C)[e]	T_s (°C)[e]	M_n (g/mol)	M_w (g/mol)	$C\#_n$	$C_{C=C}$ (mmol/g)[d]	s[g]
**PP-T0-5h**	2.0 (± 0.6)	35.0 (± 14)	31.0(± 0.76)	67	71	4.8	-	530
**PP-T1-5h**[a]	53 (± 1.2)	107 (± 2.0)	65.0 (± 4.7)	129	136	9.2	5.40 (±0.11)	
**PP-T2-5h**[c]	32 (± 4.0)	208 (± 15)	106 (± 2.8)	290	297	21	3.97 (±0.07)	
**PP-ST-5h**[c]	89 (±1.4)	215 (± 1.6)	-	420	454	30	4.54 (±0.02)	207
**PP-NT-5h**[b]	92 (±1.5)	-	-	676	700	48	-	128
**PP-STW-6h**[cf]	87 (± 2.5)	221 (± 12)	-	379	416	27	4.25 (±0.03)	229

[a] The molecular weights were evaluated by GC using Supplementary Equation 2;

[b] The molecular weights were evaluated by APCI-MS using Supplementary Equation 3;

[c] The molecular weights were evaluated by combination of APCI-MS and GC-MS;

[d] The alkenyl concentration was determined using Supplementary Equation 4;

[e] The T_f and T_s were measured using thermocouple and IR camera, respectively.

[f] The standard error was corrected with balance accuracy.

[g] The average number of scission (s) were calculated as M_0/M_t,[21] where M_0 is initial molecular weight of PP (87 kDa, Supplementary Figure 15a), and M_t is M_n as calculated in Supplementary Table 1. The M_t of three-fraction reactor was estimated as weight average of M_n.

Supplementary Table 2. CLDs evaluated by CV, PDI, σ, and S[a]

	CV	PDI	σ	S	Refs
**PP-T0-5h**	0.28	1.06	1.14	0.88	-
**PP-T1-5h**	0.502	1.05	2.15	2.48	-
**PP-T2-5h**	0.555	1.03	3.40	3.23	-
**PP-T1-1h[b]**	1.57	1.10	4.50	4.23	-
**PP-T2-1h**	5.07	1.13	12.2	4.6	-
**PP-ST-5h**	2.37	1.08	8.42	4.25	-
**PP-STW-6h**	2.66	1.10	8.48	3.53	-
**PP-NT-5h**	2.95	1.06	11.9	4.01	-
**β+Pt@Hie-TS-1**	0.23	1.04	1.14	4.25	[41]
**L-ZrO2@mSiO_2**	1.73	1.10	5.45	4.43	[40]
**UiO-66-RuH_2**	2.00	1.13	5.44	4.45	[53]
**25-mSiO_2/Pt/SiO_2**	2.05	1.08	7.25	4.81	[55]
**mSiO_2/Pt-5.0/SiO_2**	2.57	1.13	7.22	4.74	[56]
**γ-Al_2O_3**	4.58	1.23	8.60	5.11	[54]
**PP[c]**	18.7	2.79	37768	7.80	

[a] The parameters were calculated using the compositions, estimated using GC and APCI-MS

[b] The distribution was evaluated using GC-MS and it was underestimated

[c] Estimated using HT-GPC.

Supplementary Table 3. PP chain scission kinetics

**Temperature (°C)**	**Time (h)**	$\boldsymbol{M_n}$ **(g/mol)**
**360**	**1**	565
		595
	**2**	443
		463
	**4**	419
		459
	**10**	418
		420
**400**	**0.5**	486
		487
	**1**	384
		368
	**2**	285
		291

[a] The alkenyl concentration was determined using Supplementary Equation 2.

Supplementary Table 4. General parameters for ASPEN simulation of fractionated degradation.

Item	Values
**Annual capacity**	10,000 tons
**Operation hour per year**	8,000 hours
**Polymer density**[a]	0.92 g cm^{-3}
**Hydrocarbon product density**[b]	0.51 - 0.79 g mL^{-1}
**Average boiling point**[c]	177°C
**Composition input**	See Figure 1d, single fraction
**Outlet temperature**	400°C
**Outlet pressure**	1 atm
**Heat duty**	2356 MJ h^{-1}
**Heat loss**[d]	Ignored
**Heat transfer coefficient**[e]	100 BTU hr^{-1} $ft^{-2}\cdot°C^{-1}$
**Reactor volume**	12 m^3
**Tray efficiency**	100%
**Column height**	5.5 m
**Column width**	0.5 m
**Stirrer paddle size**	1 m
**Stirring rate**	300 rpm

[a] The PP density was calculated using the NIST PSTAR program with material composition data;[70]

[b] hydrocarbon densities were recalculated using α-alkene densities by weight average;[69]

[c] boiling point of hydrocarbon was measured using TGA and DSC (Supplementary Figure 16).

[d] To simplify the TEA and LCA, heat loss was not included in the calculations. With GB/T 4272-compliant equipment, the actual impact of heat loss is negligible (< 1%).

[e] Heat transfer coefficient for simulation was estimated based on the reactor dimension and PP physiochemical properties using Chilton correlation.[71]

Supplementary Table 5. General parameters for ASPEN simulation of PP upcycling to surfactants.

Components	Degradation	Sulfonation	Hydrolysis
**PP (kg/h)**	1,250	2,174	3,295
**Reaction time (h)**	10	0.5	0.5
**Reaction pressure (MPa）**	0.1	0.1	0.1
**Reaction temperature (°C)**	400	25	170
**Yield (kg/h)**	1,087	1,647.5	1,647.5
**Gas and coke (kg/h)**	163	-	-
**SO_3 (kg/h)**	**-**	957	-
**NaOH (kg/h)**	**-**	-	600
**Circulation water (kg/h)**	**-**	-	2,000
**Surfactant (kg)**		3,295	
**Crude products (kg)**		365	
Surfactant		123	
Na_2SO_4		164	
$NaHCO_3$		77.5	

Supplementary Table 6. Degradation product properties at different cutting point.

Cutting point (wt. %)	Properties	PP
**10%**	Temperature (℃)	65
	Density, kg/m³, 20℃	673.3
	Viscosity mPa s, 10℃	0.185
**30%**	Temperature (℃)	104
	Density, kg/m³, 20℃	739.5
	Viscosity mPa s, 10℃	0.325
**50%**	Temperature (℃)	152
	Density, kg/m³, 20℃	759.6
	Viscosity 1mPa s, 10℃	0.827
**60%**	Temperature (℃)	176
	Density, kg/m³, 20℃	784.9
	Viscosity mPa s, 10℃	2.13

Supplementary Table 7. ASPEN simulation of PP fractionated degradation with different number of fraction

Number of fraction	Tray label[a]	M_n	M_w
1	1-1	293.02	353.19
3	3-1	107.36	113.4
	3-2	152.58	155.43
	3-3	209.47	214.7
5	5-1	80.52	82.37
	5-2	131.39	134.46
	5-3	154.68	156.53
	5-4	222.41	222.94
	5-5	264.56	264.99
7	7-1	83.35	84.97
	7-2	101.12	103.14
	7-3	109.94	112.43
	7-4	154.68	156.53
	7-5	164.81	165.56
	7-6	222.41	222.94
	7-7	264.56	265
8	8-1	77.5	78.57
	8-2	101.47	102.88
	8-3	126.62	127.59
	8-4	162.88	164.16
	8-5	175.55	176.54
	8-6	224.84	225.29
	8-7	266.91	267.29
	8-8	308.16	308.25

Supplementary Table 8. Group additive values for vapor pressure estimation of α-alkenes.[a]

Group	a_i	b_i	c_i	d_i
$-CH_3$	11.7714	-12.5737	2.2841	-0.2608
$-CH_2-$	3.2193	-9.0445	1.3134	-0.066
$=CH_2$	11.2767	-11.1067	1.5073	-0.0519
=CH-	5.6569	-12.5108	3.8423	-0.5502

[a] Vapor pressures at 1 atm were calculated using the group additive method (Supplementary Equation 15), following the equation provided in reference:[13] The results are shown in Supplementary Tables 9 and 10

Supplementary Table 9. Estimated composition and physiochemical properties of PP-T0, -T1, and -T2.

	C#	T_f (°C)	Vapor pressure (kPa)[a]	Mole ratio (%)[b]	P_A (kPa)[c]	α[d]	η[e]
**T0**	**C3**	35	1438	22.4	452	7.31	62%
	**C4**		398	21.0			
	**C5**		120	30.7			
	**C6**		37	24.6			
	**C7**		12	1.20			
**T1**	**C6**	108	346	8.96	61.8	13.2	54%
	**C7**		152	3.81			
	**C8**		69	9.81			
	**C9**		32	52.8			
	**C10**		15	4.85			
	**C11**		7	4.45			
	**C12**		3	8.05			
	**C13**		1.5	3.01			
	**C14**		0.73	0.15			
	**C15**		0.35	3.33			
**T2**	**C15**	208	22.5	9.80	4.66	123	40%
	**C16**		14.1	5.85			
	**C18**		5.54	16.2			
	**C19**		3.50	8.67			
	**C21**		1.40	15.8			
	**C22**		0.89	8.66			
	**C23**		0.57	12.8			
	**C24**		0.36	12.5			
	**C25**		0.24	2.76			
	**C26**		0.15	2.67			
	**C27**		0.10	3.29			
	**C28**		0.06	1.00			

[a] Vapor pressure of hydrocarbons were calculated using group additive method (Supplementary Table 8) at T_f under 1 atm;[13]

[b] The column of GC-MS was operated at 300°C, which can accurately detect hydrocarbons less than C_{19}. For hydrocarbons larger than C_{20}, APCI-MS results were more accurate. Thus, composition of PP-T0-5h and PP-T1-5h were determined by GC-MS directly (Supplementary Figure 8), while PP-T2-5h was characterized by both GC-MS (C_{15}-C_{19}, Supplementary Figure 8) and APCI-MS (C_{20}-C_{28}, Supplementary Figure 9a).

[c] Molar average vapor pressure of hydrocarbons on each plates;

[d] Relative volatility (Supplementary Equation 16) of PP-T0 and -T1 was estimated over the molar average vapor pressure of the lower tray, while PP-T2-5h was estimated as ratio of P_A over C_{30} at 215°C under 1 atm (Supplementary Table 10).

[e] Viscosity of PP-T0-5h and -T1-5h were predicted using NIST Thermophysical Properties of Fluid Systems. PP-T2 was estimate using Mehrotra equation, $\lg(\mu+0.8)=100\times(0.01T)^b$.[72]

Supplementary Table 10. Vapor pressure and hydrocarbon composition of PP-ST[a]

Reactor Type	Alkene C#	T_f(°C)	Molar ratio (%)[a]	Vapor pressure (kPa)[bc]	P_A (kPa)	η
ST	**6**	215	0.013	2408.09195	2.48	16%
	**7**		0.054	1392.01613		
	**8**		0.035	821.42954		
	**9**		0.00213	492.42011		
	**10**		0.016	298.87961		
	**11**		0.09	183.24055		
	**12**		0.174	113.27942		
	**13**		0.044	70.51912		
	**15**		0.547	27.79681		
	**17**		1.943	11.1551		
	**18**		1.48	7.10506		
	**20**		2.02	2.90835		
	**21**		3.27	1.86799		
	**23**		4.77	0.77572		
	**24**		4.87	0.50135		
	**26**		3.68	0.21048		
	**27**		7.30	0.13669		
	**29**		6.52	0.05788		
	**30**		9.17	0.03773		
	**32**		4.45	0.01609		
	**33**		9.74	0.01052		
	**35**		4.87	0.00451		
	**36**		9.00	0.00296		
	**38**		3.02	0.00127		
	**39**		7.41	0.00084		
	**42**		5.71	0.00024		
	**45**		3.86	0.000069		
	**48**		2.86	0.00002		
	**51**		1.73	0.000006		
	**57**		0.64	0.000005		
	**60**		0.49	0.0000001		
NT	**15**	35	0.04	0.00199	4.25E-6	0.05%
	**18**		0.092	8.47428E-5		
	**21**		0.16	3.71057E-6		
	**24**		0.25	1.65857E-7		
	**27**		1.34	7.5312E-9		
	**30**		2.93	3.46251E-10		
	**33**		3.74	1.60799E-11		
	**36**		4.71	7.52971E-13		
	**39**		5.98	3.55059E-14		
	**42**		6.98	1.68422E-15		
	**45**		8.28	8.0301E-17		
	**48**		9.06	3.84571E-18		
	**51**		10.7	1.84898E-19		
	**54**		10.2	8.92056E-21		
	**57**		8.69	4.31713E-22		
	**60**		7.96	2.09509E-23		
	**63**		6.37	1.01929E-24		
	**66**		4.28	4.97026E-26		

Supplementary Table 10 (continued). Vapor pressure and hydrocarbon composition of PP-ST[a]

Reactor Type	Alkene C#	T_f(°C)	Molar ratio (%)[a]	Vapor pressure (kPa)[bc]	P_A (kPa)	η
NT	**69**	35	3.12	2.42861E-27	4.25E-6	0.05%
	**72**		1.90	1.18894E-28		
	**75**		1.25	5.83061E-30		
	**78**		0.83	2.86395E-31		
	**81**		0.46	1.40883E-32		
	**84**		0.36	6.93983E-34		
	**87**		0.15	3.42289E-35		
	**90**		0.17	1.69026E-36		

a Vapor pressure of hydrocarbons were calculated using group additive method (Supplementary Table 8) at T_f under 1 atm;[13]
b Molar ratio as characterized by GC-MS and APCI-MS (Figure 1);
c Molar average vapor pressure of hydrocarbons on each plates;
d Relative volatility (Supplementary Equation 16) of PP-ST and -NT was estimated over the vapor pressure of C60 and C90 at T_f.
e Viscosity of PP-ST and -NT were predicted using Mehrotra equation, $\lg(\mu+0.8)=100\times(0.01T)^b$.[72]

Supplementary Table 11. Potential additives in PP plastic wastes.[44,45]

Additive Category	Primary Function	Inference basis	Potential Compounds
Antioxidants	Prevent thermal oxidation	P、S	•Hydroxyphenyl compounds: AO-13
			•Thiodiphenol: AO-27
			• Thioethers: S-1 , S-2, S-3
			• Phosphites: P-1, P-2, P-3
Lubricants	Improve processing flow	Ca	• Internal: Calcium stearate
Impact Modifiers	Enhance toughness	C、S	• Elastomers: EPDM (vulcanization)
Fillers/Reinforcements	Increase rigidity, reduce cost	Ca、Mg	• Mineral fillers: $CaCO_3$, talc (20-40%)
			• Reinforcing fibers: Glass fiber (30%)
Colorants	Provide color/opacity	S、Na	•Cadmium Yellow<br>•Cadmium Red<br>•Ultramarine Blue
Functional Additives	Specialized properties	P、S、Br	• Flame retardants: Brominated/phosphorus: Thiobromodiphenoxyethane、TBBPA-BDBPE、Phosphorus oxide, red phosphorus, etc.

Supplementary Table 12. Element profile of degradation products of real-life PP wastes and laboratory-grade PP via various degradation methods.[a]

Elements	PP-STW-6h			PP-T2-5h		Blank [a]	PP-ST-5h		PP-W1-5h	PP-W2-5h	PP-W3-5h
	**Comprehensive scan quantification**										
**Ba**	0.195	0.193		0.0311	0.0320	0.0048	0.0153	0.0148	1.07	247	502
**Sr**	0.159	0.205		0.0210	0.0190	0.0019	0.0018	0.0055	2.81	23.4	46.5
**Na**	13.2	18.5		ND	3.81	0.4137	1.13	1.13	0.570	81.1	76.0
**Al**	ND	7.28		0.510	0.210	0.0160	0.257	0.324	72.6	393	1798
**As**	0.00700	0.0500		0.0012	0.00560	ND	0.0022	0.0001	0.0600	3.91	1.22
**Sb**	0.0100	0.0110		0.0140	0.0440	0.0001	0.0012	0.0014	0.0800	8.20	12.9
**Cd**	0.00270	0.00380		0.0016	0.0330	ND	ND	0.0003	0.01	0.290	0.480
**Cr**	0.028	0.79		0.039	0.0850	0.0022	0.584	0.519	3.46	20.2	105
**Pb**	0.458	0.820		0.120	0.280	ND	0.0210	0.0342	3.36	6.22	8.82
**Fe**	2.08	7.55		1.81	2.06	0.0045	0.493	0.825	63.3	223	1749
**Cu**	0.570	0.700		0.083	0.140	0.0138	0.0355	0.0441	2.69	6.52	54.8
**Zn**	0.860	4.50		0.110	0.450	0.0013	0.245	0.506	10.2	80.9	104
**In**	0.350	0.0130		0.004	0.006	ND	0.001	0.003	0.0100	0.0100	0.0500
	**Standard element quantification[b]**										
**Br**	27.3	26.9	26.5	-	-	-	-	-	-	-	-
**I**	1.39	1.36	1.30	-	-	-	-	-	-	-	-
**Ca**	38.3	38.6	37.7	9.86	8.88	0.0454	0.740	1.85	1.75	57.7	62.9
**Mg**	3.01	2.86	2.96	0.680	0.860	0.0021	0.0560	0.0905	36.1	2.48	2.09
**S**	43.2	42.2	43.6	4.43	5.28	ND	ND	ND	0.0300	0.190	1.07
**P**	5.89	5.48	12.8	12.8	1.57	0.0032	2.63	1.67	15.9	24.5	43.5
**Si**	0.246	1.14	0.240	ND	ND	ND	0.0289	0.0163	0.156	2.18	8.18

[a] ND = not detected; - = not analyzed

[b] The blank sample was DI water

[c] The elemental compositions were measured using standard element calibration curves (Supplementary Figure 24)

Supplementary Table 13. Device costs of different scenarios.

Scenarios	Device costs (k$)					Total (k$)
	Fractionated reactors		Drop-film reactor	Hydrolysis reactor	Dryer	
**B-SD**	440		206	103	70	819
**B-P**	440		206	103	252	1002
	Degradation reactor	Distillation tower	Drop-film reactor	Hydrolysis reactor	Dryer	
**D-SD**	103	869	206	103	70	1350
**D-P**	103	869	206	103	252	1533

Supplementary Table 14. Key assumptions about TEA.

**Fixed capital investments**		
**Direct cost (65-85% of fixed capital investment)**		
Purchased equipment		40%
Other direct costs		
	Installation	6%
	Piping	6%
	Instrumentation and control	4%
	Building & Structure	4%
	Yard improvement	4%
	Service facilities	20%
	Land	1%
Indirect costs (15-35% of fixed capital investment)		
	Engineering & supervision	10.2%
	Legal expenses	1.7%
	Construction expenses	8.5%
	Contractor's fee	3.4%
	Contingency	10.2%
Total		119%
**Other investment assumptions**		
Operating labor		20% of total product cost
Operating supervision		15% of operating labor
Maintenance and repairs		10% of Fixed capital investments
Operating supplies		30% of maintenance and repairs
Laboratory charges		10% of operating labor
waste processing		10% of Raw materials
Taxes (property)		2% of Fixed capital investments
Financing (interest)		0% of Fixed capital investments
Insurance		1% of Fixed capital investments
Rent		0 of% Fixed capital investments
Plant overhead costs.		5% of total product cost
General expenses		10% of total product cost

Supplementary Table 15. Summary of prices and emission factors.

Items		Annual Quantity	Unit price[a]	Total
**Feedstocks**				
PP waste		10,000 t	405 $/ton	$3,461,538
SO_3		3,654 t	385 $/ton	$1,204,615
NaOH		1,914 t	488 $/ton	$798,726
Surfactants		13,000 t	1,333 $/ton	$17,329,000
Eletricity	B-SD	2,514,542 kWh	0.12 $/kWh	$257,901
	B-P	4,798,542 kWh		$575,825
	D-SD	5,470,097 kWh		$656,411
	D-P	7,847,097 kWh		$930,491
**CO_2 emission factors**				
Electricity (fire), $kgCO_2e/kWh$[b]		0.557		
Truck shipping, $kgCO_2e$ /(t•km)[c]		0.147		
Coke production, $kgCO_2e$ /kg		0.470		
NaOH production, $kgCO_2e$ /kg		1.590		
SO_3 production, $kgCO_2e$ /kg		0.061		
Sulfonate surfactant production, $kgCO_2e$ /kg		1.738		

[a] The price of PP waste was the maximum value obtained by field investigation in the circular economy industrial park, Jieshou Anhui, China at July 23, 2024. Other quoted prices in Supplementary Figure 2 were converted at CNY/USD exchange rate of 7; the price of SO_3 was obtained by quoting Xingzhilian Ltd. Zibo, Shandong China; the price of NaOH was obtained from ECHEMI; The price of sulfonates surfactants was obtained from open-source market analysis report;[67]

[b] The official factor was cited from Ministry of Ecology and Environment of China. CO_2 emission factors of electricity, 2021; https://www.mee.gov.cn/xxgk2018/xxgk/xxgk01/202404/W020240412827267102800.pdf (2021).

[c] The factor was cited from CLCD. Chinese Life Cycle Database-ECER0.8.1. http://www.efootprint.net/#/home (2013).

[d] The factor was cited from the reference;

[e] The factor was cited from China City Greenhouse Gas Working Group. China Products Carbon Footprint;

[f] Factors Database. https://lca.cityghg.com (2013);

[g] The factor was cited from *New and updated life cycle inventories for surfactants used in European detergents: Summary of the ERASM surfactant life cycle and ecofootprinting project*.[73]

Supplementary Table 16. summary of TEA and LCA.

TEA	B-SD	B-P	D-SD	D-P
Fixed capital investment, $	2,437,715	2,980,771	4,018,332	4,561,389
Working capital, $	365,657	447,115	602,749	684,208
Total capital investment, $	2,803,372	3,427,887	4,621,082	5,245,597
Start-up cost, $	243,771	298,077	401,833	456,138
Operation cost, $	11,844,504	12,379,746	12,771,225	13,306,467
Cost per ton, $	1,184	1,237	1,277	1,330
Annual sales, $	14,860,395	14,860,395	14,860,395	14,860,395
Annual gross profit	3,015,890	2,480,648	2,089,169	1,553,927
Annual net profit	2,115,588	1,696,647	1,361,695	942,754
ROI, %	75.5%	49.5%	29.5%	18.0%
Payback period, yr	1.24	1.83	2.9	4.4
Discount factors	0.03	0.03	0.03	0.03
Net present worth, $	30,646,034	2,429,7164	19,056,321	12,707,451
Discounted cash flow rate of return	0.227	0.227	0.227	0.227
IRR, %	70.1%	49.8%	32.7%	22.2%

LCA		B-SD	B-P	D-SD	D-P
Yield	CO_2 emission and reduction				
87%	Transportation, $kgCO_2/ton$	14.7	14.7	14.7	14.7
	Degradation, $kgCO_2/ton$	85.9	85.9	256.2	256.2
	Sulfonation, $kgCO_2/ton$	375.0	375.0	375.0	375.0
	Drying, $kgCO_2/ton$	0.0	132.5	0.0	132.5
	**Total eimission**, $kgCO_2/ton$	475.3	607.5	645.9	778.3
	**Carbon offset benefits**, $kgCO_2/ton$	2315.8	2315.8	2315.8	2315.8
	**Net CO_2 reduction,** $kgCO_2/ton$	1840.5	1708.3	1670.0	1537.5
80%	**Total eimission**, $kgCO_2/ton$	445	577.5	615.3	747.8
	**Carbon offset benefits**, $kgCO_2/ton$	2203	2203	2203	2203
	**Net CO_2 reduction,** $kgCO_2/ton$	1758	1625.5	1587.7	1455.2
90%	**Total eimission**, $kgCO_2/ton$	488.5	625.6	659.0	796
	**Carbon offset benefits**, $kgCO_2/ton$	2425	2425	2425	2425
	**Net CO_2 reduction,** $kgCO_2/ton$	1936.5	1799.4	1766	1629

Supplementary Table 17. Representative methods for controlling product distributions.

Ref.	Method	Catalyst/System	Operation conditions	Yield (wt%)	Main Products	Distribution
53	Hydrogenolysis	UiO-66-RuH_2	200°C, 35 bar H_2, 20 h	90	alkanes	C_5-C_{19}
54		$mSiO_2$/Pt-1.7/SiO_2	300°C, 0.89 Mpa H_2, 15 h	73.6	alkanes	C_9-C_{36}
55		$mSiO_2$/Pt/MCM-48	300°C, 2.06 Mpa H_2, 6 h	43.6	hydrocarbons	C_9-C_{52}
40		L-ZrO_2@$mSiO_2$	300°C, 0.992 Mpa H_2, 20 h	28	paraffin	C_8-C_{36}
57		Ru-doped ZrO_2	250°C, 3 Mpa H_2	71	alkanes	C_4-C_{17}
56	Ball milling	γ-Al_2O_3	ball milling, r.t., ambient pressure	77.2 (wax)	hydrocarbon, alcohols, and ketons	C_7-C_{45}
1<br>20	Thermal gradient degradation	w/o catalyst	360°C ambient pressure	90 (wax or liquid)	alkenes	C_{12}-C_{68+}
This work	Fractionated degradation	w/o catalyst	400°C ambient pressure	85 (liquid)	α-olefins	C_6-C_{15}, C_{15}-C_{30}

Supplementary References

1 Zhen Xu *et al.* Chemical upcycling of polyethylene, polypropylene, and mixtures to high-value surfactants. *Science* **381**, 666-671 (2023).

2 de Groot, W. H. *Sulphonation Technology in the Detergent Industry*. (Springer Dordrecht, 1991).

3 Bolyen, E. *et al.* Reproducible, interactive, scalable and extensible microbiome data science using QIIME 2. *Nat. Biotechnol.* **37**, 852-857 (2019).

4 Tong, H. Y. & Karasek, F. W. Flame ionization detector response factors for compound classes in quantitative analysis of complex organic mixtures. *Anal. Chem.* **56**, 2124-2128 (1984).

5 Jorgensen, A. D., Picel, K. C. & Stamoudis, V. C. Prediction of gas chromatography flame ionization detector response factors from molecular structures. *Anal. Chem.* **62**, 683-689 (1990).

6 Jin, C. *et al.* Comparison of atmospheric pressure chemical ionization and field ionization mass spectrometry for the analysis of large saturated hydrocarbons. *Anal. Chem.* **88**, 10592-10598 (2016).

7 Sampling and analysis methods for domestic waste. CJ/T 313-2009, (Ministry of Housing and Urban-Rural Development of the People's Republic of China, P.R.C., 2009).

8 Standard test method for determination of the composition of unprocessed municipal solid waste. ASTM D5231-92, (ASTM, U.S.A., 2003).

9 Standard test method for foaming properties of surface-active agents. ASTM D1173−23, (ASTM, U.S.A., 2023).

10 Testing method for biodegradability of synthetic detergent. JIS K 3363-1990., (Japanese Industrial Standards Committee, Japan, 1990).

11 Schulz, J., Schäfer, K. & Bart, H. J. Entrainment control using a newly developed telecentric inline probe. *Chem. Ing. Tech.* **92**, 256-265 (2019).

12 O’Connell, H. E. Plate efficiency of fractionating columns and absorbers. *Trans. AIChE* **42**, 741–775 (1947).

13 Tu, C.-H. Group-contribution method for the estimation of vapor pressures. *Fluid Phase Equilib.* **99**, 105-120 (1994).

14 Conley, R. *Thermal stability of polymers*. (Marcel Dakker Inc., New York, 1970).

15 Schmidt, S. K., Simkins, S. & Alexander, M. Models for the kinetics of biodegradation of organic compounds not supporting growth. *Appl. Environ. Microbiol.* **50**, 323-331 (1985).

16 Poós, T. & Varju, E. Mass transfer coefficient for water evaporation by theoretical and empirical correlations. *Int. J. Heat Mass Transf.* **153**, 119500 (2020).

17 Sim, J. W. *et al.* Increasing energy saving of pilot-scale spray dryers with enhanced yield by low-adhesive surfaces. *Case Stud. Therm. Eng.* **49**, 103218 (2023).

18 Xu, Z. *et al.* Cascade degradation and upcycling of polystyrene waste to high-value chemicals. *Proc. Natl. Acad. Sci. U. S. A.* **119**, e2203346119 (2022).

19 Peters, M., Timmerhaus, K. & West, R. *Plant Design and Economics for Chemical Engineers*. 5 edn, (McGraw-Hill Education, New York, 2003).

20 Munyaneza, N. E. *et al.* Chain-length-controllable upcycling of polyolefins to sulfate detergents. *Nat. Sustain.* **7**, 1681–1690 (2024).

21 Platzer, N. *Polymer Degradation – Principles and Practical Applications*. (John Wiley & Sons, MacMillan, New York, 1982).

22 Stankiewicz, A. & Moulijn, J. A. Process intensification: transforming chemical engineering. *Chem. Eng. Prog.* **96**, 22-34 (2000).
23 Guo, W., Fan, K., Guo, G. & Wang, J. Atomic-scale insight into thermal decomposition behavior of polypropylene: A ReaxFF method. *Polym. Degrad. Stab.* **202**, 110038 (2022).
24 Peterson, J. D., Vyazovkin, S. & Wight, C. A. Kinetics of the thermal and thermo-oxidative degradation of polystyrene, polyethylene and poly(propylene). *Macromol. Chem. Phys.* **202**, 6 (2001).
25 Ruiliang Gao, Shanjun Mao, Bing Lu, Wencong Liu & Wang, Y. Efficient upcycling of polyolefin waste to light aromatics via coupling C─C scission and carbonylation. *Angew. Chem. Int. Ed. Engl.* **64**, e202424334 (2025).
26 Song, J. *et al.* Catalytic pyrolysis of waste polyethylene into benzene, toluene, ethylbenzene and xylene (BTEX)-enriched oil with dielectric barrier discharge reactor. *J. Environ. Manage.* **322**, 116096 (2022).
27 Li, S., Li, Z., Zhang, F. & Chen, J. Upgrading waste plastics to value-added aromatics. *Chem. Catalysis* **4**, 100928 (2024).
28 Odian, G. in *Principles of Polymerization*, Radical Chain Polymerization, 198-349 (John Wiley & Sons, New York, 2004).
29 Saebea, D., Ruengrit, P., Arpornwichanop, A. & Patcharavorachot, Y. Gasification of plastic waste for synthesis gas production. *Energy Rep.* **6**, 202-207 (2020).
30 Jung, M. *et al.* Analysis of chain branch of polyolefins by a new proton NMR approach. *Anal. Chem.* **88** (2016).
31 Sellers, H. & Shustorovich, E. Chemistry of sulfur oxides on transition metal surfaces: a bond order conservation-Morse potential modeling perspective. *Surf. Sci.* **356**, 209-221 (1996).
32 Joyner, N. A., Lee, Z. R. & Dixon, D. A. Binding of SO3 to group 4 transition metal oxide nanoclusters. *J. Phys. Chem. A* **127**, 9541–9549 (2023).
33 Schenk, W. A. The coordination chemistry of small sulfur-containing molecules: a personal perspective. *Dalton Trans.* **40**, 1209-1219 (2011).
34 Kaneko, M., Kumagai, S., Nakamura, T. & Sato, H. Study of sulfonation mechanism of low-density polyethylene films with fuming sulfuric acid. *J. Appl. Polym. Sci.* **91**, 2435-2442 (2003).
35 Peters, F. T., Laube, F. S. & Sadowski, G. PC-SAFT based group contribution method for binary interaction parameters of polymer/solvent systems. *Fluid Phase Equilib.* **358**, 137-150 (2013).
36 Soave, G., Gamba, S. & Pellegrini, L. A. SRK equation of state: Predicting binary interaction parameters of hydrocarbons and related compounds. *Fluid Phase Equilib.* **299**, 285-293 (2010).
37 Cai, T. J. & Chen, G. X. Liquid back-mixing on distillation trays. *Ind. Eng. Chem. Res.* **43**, 2590-2597 (2004).
38 Liu, J., Zhang, S., Wang, W. & Zhang, H. Photoelectrocatalytic principles for meaningfully studying photocatalyst properties and photocatalysis processes: from fundamental theory to environmental applications. *J. Energy Chem.* **86**, 84-117 (2023).
39 Zhang, F. *et al.* Polyethylene upcycling to long-chain alkylaromatics by tandem hydrogenolysis/aromatization. *Science* **370**, 437-441 (2020).
40 Chen, S. *et al.* Ultrasmall amorphous zirconia nanoparticles catalyse polyolefin hydrogenolysis. *Nat. Catal.* **6**, 161-173 (2023).

41 Wang, S. *et al.* Ultra-narrow alkane product distribution in polyethylene waste hydrocracking by zeolite micro-mesopore diffusion optimization. *Angew Chem. Int. Ed. Engl.* **63**, e202409288 (2024).

42 UNDP. *Plastics 101*, <https://www.undp.org/plastics-101> (2024).

43 UNDP. *Plastic Pollution*, <https://www.undp.org/chemicals-waste/plastic-pollution> (2024).

44 Hans Zweifel, M. S., Ralph D. Maier. *Plastics Additives Handbook*. 6 edn, (Hanser Publications, München, 2009).

45 Monclus, L. *et al.* Mapping the chemical complexity of plastics. *Nature* **643**, 349-355 (2025).

46 Lanorte, A. *et al.* Agricultural plastic waste spatial estimation by landsat 8 satellite images. *Comput. Electron. Agric.* **141**, 35-45 (2017).

47 Talbot, R., Granek, E., Chang, H., Wood, R. & Brander, S. Spatial and temporal variations of microplastic concentrations in Portland's freshwater ecosystems. *Sci. Total. Environ.* **833**, 155143 (2022).

48 Zhang, G., Wei, G., Liu, Z., Oliver, S. R. J. & Fei, H. A Robust sulfonate-based metal–organic framework with permanent porosity for efficient CO2 capture and conversion. *Chem. Mater.* **28**, 6276–6281 (2016).

49 Ke, L. *et al.* Polyethylene upcycling to aromatics by pulse pressurized catalytic pyrolysis. *J. Hazard. Mater.* **461**, 132672 (2024).

50 Gan, L. *et al.* Beyond conventional degradation: catalytic solutions for polyolefin upcycling. *CCS Chemistry* **6**, 313-333 (2024).

51 Shaban, S. M., Kang, J. & Kim, D.-H. Surfactants: recent advances and their applications. *Compos. Commun.* **22** (2020).

52 Gu, Y. *et al.* Critical biodegradation process of a widely used surfactant in the water environment: dodecyl benzene sulfonate (DBS). *RSC Adv.* **11**, 20303-20312 (2021).

53 Chauhan, M. *et al.* Isoreticular metal–organic frameworks confined mononuclear Ru-hydrides enable highly efficient shape-selective hydrogenolysis of polyolefins. *J. Am. Chem. Soc. Au* **3**, 3473-3484 (2023).

54 Wu, X. *et al.* Size-controlled nanoparticles embedded in a mesoporous architecture leading to efficient and selective hydrogenolysis of polyolefins. *J. Am. Chem. Soc.* **144**, 5323-5334 (2022).

55 Tennakoon, A. *et al.* Two mesoporous domains are better than one for catalytic deconstruction of polyolefins. *J. Am. Chem. Soc.* **145**, 17936-17944 (2023).

56 Li, L. *et al.* Polyethylene recycling via water activation by ball milling. *Angew Chem. Int. Ed. Engl.*, e202413132 (2024).

57 Yan, J. *et al.* Upcycling polyolefins to methane-free liquid fuel by a Ru(1)-ZrO(2) catalyst. *Nat. Commun.* **16**, 2800 (2025).

58 Xu, Z., Gao, T., Liu, X. & Xu, J. Upcycling of waste plastics: strategies, status-quo, and prospects. *Chin. Sci. Bull.* (2024).

59 Projections of Plastics Use (Projections of Plastics Use) data sets. https://data-explorer.oecd.org/ (2025)

60 Polypropylene (PP) (Eco-profiles set) data sets. (2016)

61 Chang., N. C. Strategies to reduce the global carbon footprint of plastics. *Nat. Clim. Chang.* **9**, 374–378 (2019).

62 Market volume of polypropylene worldwide from 2015 to 2022, with a forecast for 2023 to 2030. (AgileIntel Research Pvt Ltd, 2022).

63 Plastics – the fast Facts 2023. (PlasticEurope, Brussels (Belgium), 2023).

64 Polypropylene Prices, Trend, Chart, Demand, Market Analysis, News, Historical and Forecast Data Report (IMARC, 2024).

65 Polyethylene (PE) Pricing Report 2024: Price Trend, Chart, Market Analysis, News, Demand, Historical and Forecast Data. (IMARC, 2024).

66 Garcia-Gutierrez, P., Amadei, A.M., Klenert, D., Nessi, S., Tonini, D., Tosches, D., Ardente, F., Saveyn, H. Environmental and economic assessment of plastic waste recycling. (Joint Research Center, Luxembourg, 2023).

67 Linear alkylbenzene sulfonic acid price, trend and forecast. (ChemAnalyst 2024).

68 Furrer, J. A comprehensive discussion of HMBC pulse sequences, part 1: the classical HMBC. *Concepts. Magn. Reson. A* **40A**, 101-127 (2012).

69 *The Engineering ToolBox (2017)Hydrocarbons - Physical Data*, <https://www.engineeringtoolbox.com/hydrocarbon-boiling-melting-flash-autoignition-point-density-gravity-molweight-d_1966.html> (2024).

70 Stopping-Power & Range Tables for Electrons, Protons, and Helium Ions (NIST Standard Reference Database 124) data sets. (2024)

71 Chilton, T. H., Drew, T. B. & Jebens, R. H. Heat transfer coefficients in agitated vessels. *Ind. Eng. Chem.* **36**, 510-516 (1944).

72 Mehrotra, A. K. A generalized viscosity equation for pure heavy hydrocarbons. *Ind. Eng. Chem. Res.* **30**, 420-427 (1991).

73 Schowanek, D. *et al.* New and updated life cycle inventories for surfactants used in European detergents: summary of the ERASM surfactant life cycle and ecofootprinting project. *Int. J. Life Cycle Assess.* **23**, 867-886 (2017).
